# Occupational, socioeconomic factors and cancer mortality in participants of the Longitudinal Study of Adult Health (ELSA-Brazil): a multiple correspondence analysis

**DOI:** 10.1590/1980-549720250022

**Published:** 2025-05-02

**Authors:** Débora Cristina de Almeida Mariano Bernardino, Ubirani Barros Otero, Isiyara Taverna Pimenta, Luana Giatti, Rosane Harter Griep, Maria de Jesus Mendes da Fonseca

**Affiliations:** I Fundação Oswaldo Cruz, National School of Public Health – Rio de Janeiro (RJ), Brazil.; II Instituto Nacional de Câncer – Rio de Janeiro (RJ), Brazil.; III Universidade Federal de Minas Gerais – Belo Horizonte (MG), Brazil.; IV Fundação Oswaldo Cruz, Laboratory of Education in Health and the Environment – Rio de Janeiro (RJ), Brazil.

**Keywords:** Occupational cancer, Occupational exposure, Working conditions, Socioeconomic factors, Occupational health, Multivariate analysis

## Abstract

**Objective:**

To investigate the joint relationships between cancer mortality, occupational factors, and socioeconomic characteristics among Brazilian civil servants.

**Methods:**

This is a cross-sectional study with data from 116 active workers at the baseline of the Longitudinal Study of Adult Health (ELSA-Brazil) (2008–2010), who died of malignant neoplasms over a 10-year follow-up period. Multiple Correspondence Analysis was used to graphically interpret the association between occupation, work stress, working hours, work regime, and socioeconomic factors with cancer mortality.

**Results:**

The association between variable categories resulted in four groups and allowed us to identify two broad, distinct profiles of workers. The first was characterized as women, aged between 50 and 72 years, working hours of up to 40 hours a week, no exposure to night work, standard work schedule, low job strain, higher education or graduate degree level of education, active work, noncarcinogenic occupations, and death from non-work-related cancer. The second profile was characterized by men, elementary school and high school levels of education, aged between 35 and 49 years, passive work, high job strain, on-call work regime, exposure to night work, carcinogenic occupations, and death from work-related cancer.

**Conclusion:**

Work-related cancer death was associated with worse socioeconomic conditions and occupational circumstances unfavorable to workers’ health.

## INTRODUCTION

Work-related cancer (WRC) is the second leading cause of death among workers worldwide. In 2019, 2.9 million work-related deaths occurred, 29% of which were caused by malignant neoplasms^1,2^. In Brazil, the WRC mortality rate tended to increase in more than half of the states (1990–2019)^3^. In the Longitudinal Study of Adult Health (from Portuguese, *Estudo Longitudinal de Saúde do Adulto* – ELSA-Brazil), one of the largest epidemiological studies in Latin America, cancer emerged as the main cause of death among participating workers over 10 years of observation (2008–2018)^4^. Despite the multifactorial nature of cancer and the lack of understanding of all the factors involved, work has stood out as an element of relevant contribution to this scenario.

In this sense, the literature points to a set of types of cancer that have already established a relationship with chemical, physical, or biological agents to which workers are exposed in their work environment^5^. Authors of a recent study used data from the monographs of the International Agency for Research on Cancer (IARC) and listed 47 agents in group 1, including asbestos, solar radiation, and benzene, associated with hematologic, lung, bladder, liver, and stomach cancers, among others^6^. There is, therefore, sufficient evidence that occupational exposure to carcinogens predisposes individuals to malignancies and deaths caused by the disease^6,7^.

Conversely, little is known about other circumstances of exposure intrinsic to the work process that can also contribute to illness and death from cancer. Researchers suggest that stress in the occupational context^8^, long working hours^9^, shift work and night work^10^ may represent an increased risk for certain types of cancer, such as lung^8^ and breast^9,10^, contributing to death^11^. However, the literature is still incipient on the subject and sometimes controversial, especially when dealing with the association between such exposures and cancer mortality.

To date, there are no national studies focused on investigating the joint relationships of occupational factors and cancer mortality in the Brazilian context. Therefore, in addition to the already recognized carcinogenic exposures, and seeking to contribute to the elucidation of the relationships between occupational factors and cancer, in this study we aimed to investigate the joint relationships between cancer mortality, occupational factors, and socioeconomic characteristics among Brazilian civil servants who died in a 10-year follow-up period in the ELSA-Brazil workers’ cohort.

## METHODS

### Study design

This is a cross-sectional analysis with data from ELSA-Brazil, which is a multicentric cohort that investigates determinants of chronic noncommunicable diseases and their incidences. The baseline (2008–2009) included 15,105 civil servants, aged between 35 and 74 years, from six higher education institutions in the Northeast, South, and Southeast regions of Brazil. The study methodology involves the monitoring of incident outcomes through visits every four years and annual follow-up interviews, which monitor events via telephone. When an event is identified, an investigation begins, accessing medical records for additional information. Data on deaths are collected in the institutions’ human resources sector and in the Brazilian Mortality Information System. The methodological aspects of ELSA-Brazil are detailed in previous studies^12,13,14^. This study used baseline information from the cohort (2008–2010) and on deaths that occurred until December 31, 2018.

### Participants

Active workers from the baseline (2008–2010) of ELSA-Brazil who died of cancer until 2018 were included. Retired participants were excluded for lack of information on the psychosocial work environment as well as those with missing data on other variables.

### Variables

The used variables were collected through questionnaires applied in the ELSA-Brazil interviews and categorized as follows:

- WRC deaths: cancer deaths in the cohort were classified as work-related or non-work-related, based on the degree of carcinogenicity of occupational agents defined by IARC^5^. For classification as death due to WRC, the type of cancer recorded should be associated with at least two carcinogens (group 1), aiming at bringing greater precision to the classification, making the study population more heterogeneous, and identifying different groups in the multiple correspondence analysis. Typologies that did not meet this criterion were classified as non-work-related cancer death (Chart 1);
- Occupation: in ELSA-Brazil, the occupations of the participants were defined according to the Brazilian Classification of Occupations (*Classificação Brasileira de Ocupações* – CBO), which has four hierarchical levels: large groups (LG), main subgroups (MS), subgroups (SG), and baseline groups (BG), the latter being the most specific and analyzed in this study. The BG level includes job title, description of occupation, main activities, and working conditions such as exposures to chemical, physical, and biological agents^15^. Based on the description of the activities and working conditions extracted from the CBO, the occupations were evaluated as for possible exposure to carcinogens and categorized into two groups: carcinogenic (exposure to carcinogens of group 1 or 2A of IARC)^5^ and noncarcinogenic (without evidence of exposure to such agents in their work environments) (Chart 2);
- Work regime: it was categorized as standard work schedule, covering workers who carry out their work activities from Monday to Friday, at fixed times, exclusively during the day, and who, throughout their professional life, have never performed on-call work; and on-call work, covering workers who follow work schedules that include weekends, holidays, and hours both on day and night shifts, in the current or previous days;
- Working hours: subdivided into up to 40 hours a week and over 40 hours a week;
- Exposure to night work: defined by means of two distinct categories, night work (yes), referring to individuals who perform or have performed at some point in their professional life work activities on the night shift (between 10 pm and 5 am), as determined by Brazilian legislation^16^; and night work (no), encompassing those who have never had the experience of working at night;
- Stress at work: it was measured using the Swedish Job Demand-Control-Support Model scale^17^, validated for use in Brazil^18^, applied by trained researchers. The scale considers two dimensions of the psychosocial work environment: psychological demand, determined by items related to workload, the pace of activities, and the difficulty in performing them; and work control, determined by items related to the use of intellectual skills and the degree of autonomy for decision-making. The obtained scores, ranging from five to 20 points for each dimension, were dichotomized based on their medians (15 points for psychological demands and 17 for work control). This culminated in the four quadrants proposed by Karasek^19^: low job strain (low demand/high control); active work (high demand/high control); passive work (low demand/low control); and high job strain (high demand/low control);
- Age: categorized into two groups, 35–49 and 50–72 years, considering the increase in cancer mortality from 50 years onward^20^;
- Sex: categorized into men and women;
- Level of education: classified into three categories: up to complete elementary school; complete high school; and higher education or more.

**Chart 1:** Classification of causes of cancer death in the population of the Longitudinal Study of Adult Health (ELSA-Brazil).

Typology of cancer deaths (ELSA-Brazil)	Agents provided for in group 1 of the International Agency for Research on Cancer (IARC) that may be present in the work environment	Classification of deaths
**Lung**	Acheson process; aluminum production; arsenic; asbestos; beryllium; bis(chloromethyl) ether, chloromethyl methyl ether; cadmium; chromium (VI); coal gasification; coal tar pitch; coke production; diesel engine exhaust; underground hematite mining; iron and steel smelting; combined chemotherapy including alkylating agents; nickel compounds; outdoor pollution; occupational exposure as a painter; plutonium; radon-222 and its decomposition products; rubber manufacturing industry; silica dust, crystalline silica in the form of quartz or cristobalite; soot; passive smoking (tobacco); welding fumes; X and gamma radiation	D:WRC
**Breast**	X and gamma radiation	D:NWRC
**Colorectal**	X and gamma radiation	D:NWRC
**Liver**	Aflatoxins; hepatitis B and C viruses (chronic infection); plutonium; thorium-232; vinyl chloride	D:WRC
**Stomach**	X and gamma radiation; rubber manufacturing industry	D:WRC
**Bladder**	Aluminum production; 4-aminobiphenyl; arsenic; auramine; benzidine; cyclophosphamide; firefighter; magenta production; 2-naphthylamine; painter; rubber manufacture; ortho-toluidine; ionizing radiation	D:WRC
**Leukemia**	Benzene; formaldehyde; combined chemotherapies; phosphorus-32; thorium-232; X and gamma radiation; 1,3-butadiene; rubber manufacturing industry	D:WRC
**Kidney**	Trichloroethylene; X and gamma radiation	D:WRC
**Brain and central nervous system**	X and gamma radiation	D:NWRC
**Cervical and ovarian cancer**	Asbestos; infection [acquired immunodeficiency virus (HIV) type 1]; human papillomavirus types 6, 18, 31, 33, 35, 39, 45, 51, 52, 56, 58 and 59	D:WRC
**Myeloma**	1,3-Butadiene; pentachlorophenol	D:WRC
**Lymphoma**	Epstein-Barr virus; HIV type 1 and hepatitis C; azathioprine; cyclosporine; lindane; pentachlorophenol	D:WRC
**Upper aerodigestive tract/prostate/pancreas other cancers**	–	D:NWRC

D:WRC: work-related cancer death, based on cancer typologies associated with at least two agents listed in IARC Group 1; D:NWRC: non-work-related cancer death.

Source: Adapted from the International Agency for Research on Cancer (IARC)^5^.

**Chart 2:** Classification of the occupation variable in the population of the Longitudinal Study of Adult Health (ELSA-Brazil)*.

Occupation	n	Description of work activities according to the Brazilian Classification of Occupations	Agent or exposure circumstances pertinent to the exercise of the occupation	Classification
Administrators	1	Plan, organize, control, and advise organizations	–	OC:NC
Accountants and the like	2	Manage costs; manage personnel department	–	OC:NC
Clerks in general	13	Provide support services in the areas of human resources, administration, and finance	–	OC:NC
Pharmacists	1	Work in the development, production, dispensation, control, storage, distribution, and transportation of medicines, immunobiologicals, household cleaning products	Cyclophosphamide(1); chlorambucil (1); MOPP (chemotherapeutics) (1); hydrazine (2A); night work (2A)	OC:C
Telephone operators	1	Operate equipment, answer, transfer, register, and complete phone calls	–	OC:NC
Higher Education professors	18	Teach, carry out scientific research, advise students	–	OC:NC
Information workers	6	Provide information, manage units such as libraries, documentation centers	–	OC:NC
Professionals of economic and financial administration	1	Prepare and consolidate management and economic-financial information	–	OC:NC
Statisticians	1	Draw samples; analyze and process data; build data collection instruments; create databases	–	OC:NC
Receptionists	5	Provide reception and support services	–	OC:NC
Administrative supervisors	24	Supervise administrative routines, leading clerks, administrative assistants, office secretaries, office machine operators	–	OC:NC
Graphic arts supervisors	1	Manage the graphic production process concerning costs, feasibility of execution, task flow	–	OC:NC
Workers in the services of waste collection, cleaning and conservation of public areas	34	Collect solid waste from healthcare services, sweep, pack the garbage, preserve public areas by washing and painting them. Activities carried out indoors or outdoors. Shift and night work, exposure to noise and vehicle pollution	Asbestos (1); crystalline silica dusts (1); solar radiation (1); benzene (1); diesel fumes (2A);	OC:C
Nursing technicians and nursing assistants	2	Nursing technical activities in hospitals and clinics. Administer medicines, work with surgical instrumentation, can be exposed to biological contamination, toxic material, and radiation. Work under pressure, elevating the stress situation	MOPP (chemotherapeutics) (1); viruses (hepatitis B and C; Epstein-Barr; human immunodeficiency type 1; Human T-lymphotropic virus type 1 (1); ethylene oxide (1); ionizing radiation (1); night work (2A);	OC:C
Library technicians	1	Work in libraries, schools, research, and statistical institutes	–	OC:NC
Environmental control, utilities, and effluent treatment technicians	1	Preserve environmental quality. Work in laboratories, in field activities, indoors and outdoors. Day and night hours. Exposed to noise, toxic material, radiation.	Benzene (1); cadmium (1); solar radiation (1); night work; lead (2A)	OC:C
Medical and dental equipment technicians	3	Manufacture and repair human, animal, and artistic dental prostheses. May be subject to toxic material exposures, radiation, working pressure that can lead to stress	Ionizing radiation (1); nickel and its compounds (1)	OC:C
Chemical technicians	1	Operate chemical processes and unit laboratory and production operations. May be subject to noise, dust, gases, vapors, and toxic material	Vinyl chloride (1); cadmium and its compounds (1); benzene (1); sulfuric acid (1); trichloroethylene (1)	OC:C

*Classification of the occupation variable of active workers, in the ELSA-Brazil study population, according to occupational exposures, to at least one chemical, physical, or biological agent, or exposure circumstance with the potential to cause cancer (group 1 and 2A of the International Agency for Research on Cancer), pertinent to the exercise of the occupation; OC:C: carcinogenic occupation; OC:NC: noncarcinogenic occupation. Description of activities adapted from the Brazilian Classification of Occupations (CBO)^15^.

### Statistical analysis

Multiple correspondence analysis (MCA) was used to investigate the joint relationships between cancer mortality and occupational factors, including the socioeconomic characteristics of the study population. Categorical variables were described by absolute and relative frequencies. The MCA allowed the graphical representation of the behavior profile of the group of qualitative variables analyzed.

Correspondence analysis is a non-inferential technique applied to categorical data intended to compose a pattern of association between the study variables, by measuring the distance χ^2^ between the categories that compose the set of variables. Each category had a spatial position in the correspondence analysis graph, also called a perceptual map, and the proximity of these points indicates the degree of similarity between them. This means that categories that are close together in space have similar patterns of association with each other. In turn, categories that are distant from each other on the map have different association patterns^21^.

This statistical technique presents a measure of inertia, which indicates the percentage of variability explained by dimension. The number of dimensions selected in this study was based on the decline of adjusted inertia, that is, on eigenvalues. The x-axis of the scatter plot represents the variability of the data explained by the first dimension, while the y-axis represents the variability of the data explained by the second dimension. The points represent each of the variable categories, while the lines delimit the groups associated with these categories^21^.

The analyses were carried out in the R software^16^, version 4.3.2, “ca” library.

### Ethical issues

ELSA-Brazil was approved by the Research Ethics Committees (*Comitês de Ética em Pesquisa* – CEP) of each institution involved and by the Brazilian National Council for Research Ethics in 2006. The present study was approved by the CEP of the National School of Public Health (approval number: 66407322.3.0000.5240).

## RESULTS

Of the 15,105 ELSA-Brazil participants, 12,096 were active workers and 3,009, retired. Among the active individuals, 118 died of cancer during the follow-up period. Among the eligible participants, two were excluded due to lack of information on the occupation variable. Thus, the size of the study population was 116.

Of the 116 deaths, 46.6% occurred among women (n=54) and 53.4%, among men (n=62). Age ranged from 35 to 72 years (mean=53.8; standard deviation=7.8; median=54), 39.5% of participants had higher education or more (n=46), and 60.3% of deaths were from non-work-related cancer (n=70) (Table 1).

**Table 1:** Distribution of socioeconomic and occupational characteristics according to work-related or non-work-related cancer mortality in the population of the Longitudinal Study of Adult Health (ELSA-Brazil), 2008–2010, 2018.

Variables and categories	Total cancer deaths (116)			
	Work-related cancer death n=46 (39.7%)		Non-work-related cancer death n=70 (60.3%)	
	n	%	n	%
**Sex**				
Men	28	45.2	34	54.8
Women	18	33.3	36	66.7
**Age group (years)**				
35–49	19	48.8	20	51.2
50–72	27	35.0	50	65.0
**Level of education**				
Elementary school or less	15	48.3	16	51.7
High school	16	41.0	23	59.0
Higher education or more	15	32.6	31	47.4
**Occupation**				
Carcinogenic	24	54.6	20	45.4
Noncarcinogenic	22	30.6	50	69.4
**Work regime**				
Standard schedule	25	39.0	39	61.0
On-call work	21	40.3	31	59.7
**Working hours (weekly hours)**				
Up to 40	31	35.2	57	64.8
Over 40	15	53.6	13	46.4
**Night work**				
Yes	19	43.2	25	56.8
No	27	37.5	45	62.5
**Stress at work**				
High strain	12	42.9	16	57.1
Low strain	8	40.0	12	60.0
Active work	6	26.0	17	74.0
Passive work	20	44.4	25	55.6

Source: Longitudinal Study of Adult Health (ELSA-Brazil). Baseline (2008–2010) and death adjudication base (2018).

**Figure 1:**
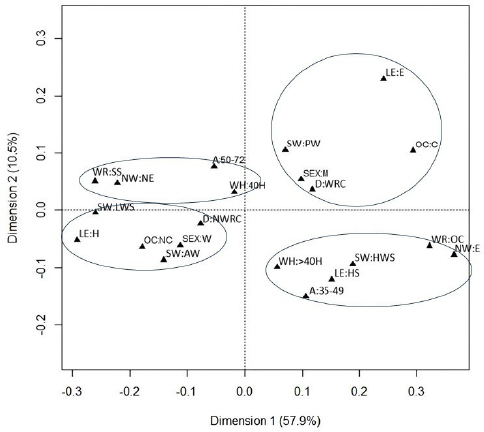
Perceptual map (two-dimensional plot) of the multiple correspondence analysis between participants of the Longitudinal Study of Adult Health (ELSA-Brazil) who died of cancer 2008–2010, 2018.

In MCA, the first two dimensions concentrated the highest proportion of explained variability (68.4%). In the two-dimensional perceptual map, we can visualize two large distinct profiles and four groups within the quadrants (Figure 1).

OC:C: carcinogenic occupation; OC:NC: noncarcinogenic occupation; NW:E: exposure to night work; NW:NE: no exposure to night work; SW:HWS: high work strain; SW:LWS: low work strain; SW:PW: passive work; SW:AW: active work; WR:SS: standard work schedule shift; WR:OC: on-call work shift; WH:≤40H: working hours up to 40 hours a week; WH:>40H: working hours over 40 hours a week; SEX:M: men; SEX:W: women; LE:H: higher education or more; LE:HS: complete high school; LE:E: elementary school or less; A:35-49: age between 35 and 49 years; A:50-72: age between 50 and 72 years; D:WRC: work-related cancer death; D:NWRC: non-work-related cancer death.

The profile located to the left of the graph gathers socioeconomic and occupational characteristics, such as high level of education, advanced age, women, no exposure to night work, standard work schedule, working hours limited to 40 hours a week, low job strain, noncarcinogenic occupations, active work, and death from non-work-related cancer.

In turn, the profile located to the right of the graph has expressly opposite characteristics: low level of education, younger age, men, exposure to night work, on-call work regime, working hours that exceed 40 hours a week, high job strain, carcinogenic occupation, passive work, and death from WRC.

Regarding the groups of variables with similarities, observed in quadrants in the graph, we can state that: the first group formed (upper left quadrant) is characterized by age between 50 and 72 years, working hours of up to 40 hours a week, no exposure to night work, and standard work schedule regime.

The second group (lower left quadrant) is characterized by women, higher education or graduate degree levels of education, active work, occupations classified as noncarcinogenic, low job strain, and death from non-work-related cancer.

In turn, the third group (upper right quadrant) was formed by men, elementary school level of education, passive work, carcinogenic occupations, and death from WRC.

In the last group (lower right quadrant), we can observe age between 35 and 49 years, high school level of education, high job strain, on-call work regime, and exposure to night work.

## DISCUSSION

In this study, through the ACM, we analyzed the relationships between occupational and socioeconomic factors and cancer. The worse working conditions (passive work and carcinogenic occupations), low level of education (elementary school), and men were associated with death from WRC. In contrast, better working conditions, high levels of education, and women were associated with death from non-work-related types of cancer.

Some authors reported the predominance of men with elementary or high school levels of education among cases of WRC^22,23^. According to our results, we found differences between sexes, suggesting that the higher prevalence of men in work activities at higher risk of exposure to carcinogens contributes to the profile of deaths from WRC. In the study by Nogueira et al.^24^, with data from the National Survey of Health (2019), 49% of men were exposed to at least one carcinogen in group 1 and 31.9% to one carcinogen in group 2 of the IARC, while among women these percentages were 16.9 and 16.5%, respectively. According to Silva et al.^25^, men, compared to women, seek healthcare services less often. Therefore, the diagnosis and treatment of diseases, such as cancer, occur late, worsening the prognosis.

Regarding the educational aspect, although ELSA-Brazil consists of federal civil servants, 70% of the baseline cohort members had level of education of up to high school. Of these, 50% performed support functions, requiring only some elementary school, and 50% high-school level functions^13^. According to Silva et al.^26^, less qualified professionals face greater risks of exposure to substances such as benzene, asbestos, heavy metals, and other carcinogens. Such conditions are verified in greater intensity in developing countries^27^, such as Brazil, and could be observed in our study, when analyzing the general conditions of exercise and exposures pertinent to occupations present in the cohort (Chart 2).

In this sense, we highlight the occupations related to the services of waste collection, cleaning, and conservation of public areas carried out by active workers participating in ELSA-Brazil. Due to work activities, these professionals may be exposed to solar radiation, mineral dust, such as silica and asbestos, and chemical substances such as benzene (details available in the Supplementary Material).

Corroborating our findings, authors of a recent study on the prevalence of occupational carcinogens in the Brazilian population, with data from the National Survey of Health, showed that 64.4% of male garbage collectors and similar workers are exposed to at least one carcinogen in group 1, and 29% to at least one carcinogen in group 2, with emphasis on the following exposures: solar radiation (58.2%), mineral dust (14.8%), and chemical agents (16%)^24^. Our findings converge with this research and with that of Bovio et al.^28^; both reinforce the established association between occupation and exposure to carcinogens, leading to excess death from cancer among workers.

According to the joint analysis of the studied variables, not only the influence of exposure to carcinogens was demonstrated, but also that of socioeconomic factors and the psychosocial work environment on deaths from WRC, an aspect still little investigated in Brazil. Researchers have been discussing the role of psychosocial stress on adverse health effects, the occurrence of diseases, and death^29,30^; however, its participation in the development and prognosis of malignant tumors, especially in the context of work, is still controversial.

In this sense, authors of some international studies point to a positive association between psychosocial stress at work and the development of malignant tumors^31^ and metastasis^32^, while others do not identify this association^33,34,35^.

Although there is no consensus among current studies, our interpretation is based on the biological plausibility that psychosocial stress at work favors primary cancer activation and accelerated tumor progression. This process involves the activation of the hypothalamic-pituitary-adrenal axis and the nervous system, releasing hormones, such as cortisol and norepinephrine, which alter the tumor microenvironment, promote inflammation, increase the expression of oncogenes, and suppress the immune response^36^. Thus, we can assume that the persistent stimulation of psychosocial stressors, such as low decision-making power and low demand, pertinent to passive work, represent a condition of chronic work-related stress. Although these stressors are not capable of causing cancer alone, when associated with other aforementioned occupational factors, they can contribute to death from WRC. It should be noted that passive work is characterized by low demand and lack of control of tasks, limiting intellectual skills, generating discouragement, frustration, and disinterest in work.

Another dimension of occupational stress we investigated in this study was high strain, represented by high psychological demands and low control for decision-making at work^18^. In a Swedish cohort study, low control and high work demands were slightly associated with an increased risk of breast cancer among women^37^. According to our results, high job strain was associated with other categories of occupational variables: on-call work regime and exposure to night work. Such occupational factors, *a priori* not classified in group 1 as known to be carcinogenic to humans, according to the current scientific literature, may contribute to a worsening of health status^38,39^, by altering healthy lifestyle habits^40^, affecting the immune system^41^, deregulating the circadian rhythm^42^, providing a favorable physiological environment for the initiation of cancer cells as well as the development of other physical and mental health issues^43^.

The hypothesis of the present study was that the occupational variables high strain, exposure to night work, and on-call work regime, for having a pattern of similarity, can contribute to the construction of a profile of people with a greater propensity to having cancer. We emphasize that the non-association of the aforementioned occupational variables with death from WRC in our investigation is possibly due to the fact that the types of cancer associated with these exposures (breast, prostate, and colorectal) were not classified in this study as deaths from WRC, according to the criteria established for the classification of this variable (for more details, see the Supplementary Material).

A curious aspect in our study concerns the workload. The working hours category above 40 hours a week was associated with women and active work (with high demands and high decision-making power); noncarcinogenic occupations; death from non-work-related cancer; and high level of education, contrasting our initial hypotheses that long working hours were associated with worse working conditions, propensity to developing and death from cancer.

Although it is not possible to completely rule out this possibility, currently, few studies have reported associations between long working hours and cancer. What has been observed in the literature is the association of this variable with other negative health outcomes, such as heart diseases and obesity^44,45^, but there are no conclusive findings on the incidence or mortality from malignant neoplasms^9,46^. Therefore, we suggest that the absence of an association between working hours greater than 40 hours a week and deaths from WRC in our study may partly reflect these findings observed in the literature.

This is the first study to evaluate cancer mortality data in workers from the ELSA-Brazil cohort. Among the advantages of the adopted method, we can mention the possibility of a robust exploratory analysis, with a flexible and visually intuitive approach. It allowed us to clearly describe the profile of the workers participating in the study and the complex relationships between occupational and socioeconomic factors and cancer mortality, overcoming the restrictions of traditional statistical methods with strict assumptions of data normality and linearity of relationships.

Conversely, we recognize that this study has limitations, as it is an investigation using non-inferential tools and a relatively small sample, as the intention was to explore only the data of active participants who died from cancer. In addition, ELSA-Brazil is not a specific study for cancer, and the classification of deaths due to WRC presents challenges. Nevertheless, we adopted a criterion of greater specificity for this classification, in order to make it more accurate. Also in relation to death, as it is a complex event, it cannot be guaranteed that its cause is strictly related to primary cancer. It is noteworthy that the study design does not allow risk assessment.

According to our results, exposures such as passive work, carcinogenic occupations, and low levels of education, together, are associated with mortality from WRC, which may indicate that worse socioeconomic conditions, occupations with a greater possibility of exposure to carcinogenic agents in the work environment, and occupational circumstances unfavorable to health present a similar association pattern, forming a characteristic profile of worse prognosis of the disease. Taking this into consideration, it is necessary to implement occupational health policies and actions capable of contributing to the improvement of working conditions, focusing on the qualification and appreciation of the professional, and the adoption of strategies to effectively reduce the risks of exposure to the previously mentioned occupational factors, seeking to promote a healthier and safer work environment, preventing illness and death from WRC.

## Data Availability

The study results from the doctoral dissertation of the Graduate Program of the National School of Public Health, subarea of Epidemiology in Public Health, approved by the Research Ethics Committee, under number: 66407322.3.0000.5240, carried out with its own funding. ELSA-Brazil was funded by the Brazilian Ministry of Health (Department of Science and Technology) and the Brazilian Ministry of Science, Technology and Innovation (Funding agency of Studies and Projects and the National Council for Scientific and Technological Development, scholarship numbers: 01 06 0010.00 RS, 01 06 0212.00 BA, 01 06 0300.00 ES, 01 06 0278.00 MG, 01 06 0115.00 SP and 01 06 0071.00 RJ)
