# ERRATUM

**DOI:** 10.1590/1980-549720250022erratum

**Published:** 2025-06-27

In the manuscript "*Occupational, socioeconomic factors and cancer mortality in participants of the Longitudinal Study of Adult Health (ELSA-Brasil): a multiple correspondence analysis*", DOI: https://doi.org/10.1590/1980-549720250022, published in the Rev Bras Epidemiol 2025; 28: e250022.

## Title:

**Where it reads:**

Occupational, socioeconomic factors and cancer mortality in participants of the Longitudinal Study of Adult Health (ELSA-Brazil): a multiple correspondence analysis

**It should read:**

Occupational, socioeconomic factors and cancer mortality in participants of the Longitudinal Study of Adult Health (ELSA-Brasil): a multiple correspondence analysis

## Abstract:

**Where it reads:**

This is a cross-sectional study with data from 116 active workers at the baseline of the Longitudinal Study of Adult Health (ELSA-Brazil) (2008–2010), who died of malignant neoplasms over a 10-year follow-up period.

**It should read:**

This is a cross-sectional study with data from 116 active workers at the baseline of the Longitudinal Study of Adult Health (ELSA-Brasil) (2008–2010), who died of malignant neoplasms over a 10-year follow-up period.

**Where it reads:**

HOW TO CITE THIS ARTICLE: Bernardino DCAM, Otero UB, Pimenta IT, Giatti L, Griep RH, Fonseca MJM. Occupational, socioeconomic factors and cancer mortality in participants of the Longitudinal Study of Adult Health (ELSA-Brazil): a multiple correspondence analysis. Rev Bras Epidemiol. 2025; 28: e250022. https://doi.org/10.1590/1980-549720250022

**It should read:**

HOW TO CITE THIS ARTICLE: Bernardino DCAM, Otero UB, Pimenta IT, Giatti L, Griep RH, Fonseca MJM. Occupational, socioeconomic factors and cancer mortality in participants of the Longitudinal Study of Adult Health (ELSA-Brasil): a multiple correspondence analysis. Rev Bras Epidemiol. 2025; 28: e250022. https://doi.org/10.1590/1980-549720250022

## INTRODUCTION

**Where it reads:**

In the Longitudinal Study of Adult Health (from Portuguese, Estudo Longitudinal de Saúde do Adulto – ELSA-Brazil), one of the largest epidemiological studies in Latin America, cancer emerged as the main cause of death among participating workers over 10 years of observation (2008–2018)^4^.

**It should read:**

In the Longitudinal Study of Adult Health (from Portuguese, Estudo Longitudinal de Saúde do Adulto – ELSA-Brasil), one of the largest epidemiological studies in Latin America, cancer emerged as the main cause of death among participating workers over 10 years of observation (2008–2018)^4^.

**Where it reads:**

Therefore, in addition to the already recognized carcinogenic exposures, and seeking to contribute to the elucidation of the relationships between occupational factors and cancer, in this study we aimed to investigate the joint relationships between cancer mortality, occupational factors, and socioeconomic characteristics among Brazilian civil servants who died in a 10-year follow-up period in the ELSA-Brazil workers’ cohort.

**It should read:**

Therefore, in addition to the already recognized carcinogenic exposures, and seeking to contribute to the elucidation of the relationships between occupational factors and cancer, in this study we aimed to investigate the joint relationships between cancer mortality, occupational factors, and socioeconomic characteristics among Brazilian civil servants who died in a 10-year follow-up period in the ELSA-Brasil workers’ cohort.

## METHODS

**Where it reads:**

This is a cross-sectional analysis with data from ELSA-Brazil, which is a multicentric cohort that investigates determinants of chronic noncommunicable diseases and their incidences.

**It should read:**

This is a cross-sectional analysis with data from ELSA-Brasil, which is a multicentric cohort that investigates determinants of chronic noncommunicable diseases and their incidences.

**Where it reads:**

The methodological aspects of ELSA-Brazil are detailed in previous studies^12-14^.

**It should read:**

The methodological aspects of ELSA-Brasil are detailed in previous studies^12-14^.

**Where it reads:**

Active workers from the baseline (2008–2010) of ELSA-Brazil who died of cancer until 2018 were included.

**It should read:**

Active workers from the baseline (2008–2010) of ELSA-Brasil who died of cancer until 2018 were included.

**Where it reads:**

The used variables were collected through questionnaires applied in the ELSA-Brazil interviews and categorized as follows

**It should read:**

The used variables were collected through questionnaires applied in the ELSA-Brasil interviews and categorized as follows

**Where it reads:**

Occupation: in ELSA-Brazil, the occupations of the participants were defined according to the Brazilian Classification of Occupations (Classificação Brasileira de Ocupações – CBO), which has four hierarchical levels: large groups (LG), main subgroups (MS), subgroups (SG), and baseline groups (BG), the latter being the most specific and analyzed in this study.

**It should read:**

Occupation: in ELSA-Brasil, the occupations of the participants were defined according to the Brazilian Classification of Occupations (Classificação Brasileira de Ocupações – CBO), which has four hierarchical levels: large groups (LG), main subgroups (MS), subgroups (SG), and baseline groups (BG), the latter being the most specific and analyzed in this study.

**Where it reads:**

Chart 1. Classification of causes of cancer death in the population of the Longitudinal Study of Adult Health (ELSA-Brazil).

**It should read:**

Chart 1. Classification of causes of cancer death in the population of the Longitudinal Study of Adult Health (ELSA-Brasil).

**Where it reads:**

Typology of cancer deaths (ELSA-Brazil)

**It should read:**

Typology of cancer deaths (ELSA-Brasil)

**Where it reads:**

Chart 2. Classification of the occupation variable in the population of the Longitudinal Study of Adult Health (ELSA-Brazil)*.

**It should read:**

Chart 2. Classification of the occupation variable in the population of the Longitudinal Study of Adult Health (ELSA-Brasil)*.

**Where it reads:**

*Classification of the occupation variable of active workers, in the ELSA-Brazil study population, according to occupational exposures, to at least one chemical, physical, or biological agent, or exposure circumstance with the potential to cause cancer (group 1 and 2A of the International Agency for Research on Cancer), pertinent to the exercise of the occupation; OC:C: carcinogenic occupation; OC:NC: noncarcinogenic occupation.

**It should read:**

*Classification of the occupation variable of active workers, in the ELSA-Brasil study population, according to occupational exposures, to at least one chemical, physical, or biological agent, or exposure circumstance with the potential to cause cancer (group 1 and 2A of the International Agency for Research on Cancer), pertinent to the exercise of the occupation; OC:C: carcinogenic occupation; OC:NC: noncarcinogenic occupation.

**Where it reads:**

ELSA-Brazil was approved by the Research Ethics Committees (Comitês de Ética em Pesquisa – CEP) of each institution involved and by the Brazilian National Council for Research Ethics in 2006. The present study was approved by the CEP of the National School of Public Health (approval number: 66407322.3.0000.5240).

**It should read:**

ELSA-Brasil was approved by the Research Ethics Committees (Comitês de Ética em Pesquisa – CEP) of each institution involved and by the Brazilian National Council for Research Ethics in 2006. The present study was approved by the CEP of the National School of Public Health (approval number: 66407322.3.0000.5240).

## RESULTS

**Where it reads:**

Of the 15,105 ELSA-Brazil participants, 12,096 were active workers and 3,009, retired. Among the active individuals, 118 died of cancer during the follow-up period.

**It should read:**

Of the 15,105 ELSA-Brasil participants, 12,096 were active workers and 3,009, retired. Among the active individuals, 118 died of cancer during the follow-up period.

**Where it reads:**

Table 1. Distribution of socioeconomic and occupational characteristics according to work-related or non-workrelated cancer mortality in the population of the Longitudinal Study of Adult Health (ELSA-Brazil), 2008–2010, 2018.

**It should read:**

Table 1. Distribution of socioeconomic and occupational characteristics according to work-related or non-workrelated cancer mortality in the population of the Longitudinal Study of Adult Health (ELSA-Brasil), 2008–2010, 2018.

**Where it reads:**

Source: Longitudinal Study of Adult Health (ELSA-Brazil). Baseline (2008–2010) and death adjudication base (2018).

**It should read:**

Source: Longitudinal Study of Adult Health (ELSA-Brasil). Baseline (2008–2010) and death adjudication base (2018).

**Where it reads:**

Figure 1. Perceptual map (two-dimensional plot) of the multiple correspondence analysis between participants of the Longitudinal Study of Adult Health (ELSA-Brazil) who died of cancer 2008–2010, 2018.

**It should read:**

Figure 1. Perceptual map (two-dimensional plot) of the multiple correspondence analysis between participants of the Longitudinal Study of Adult Health (ELSA-Brasil) who died of cancer 2008–2010, 2018.

## DISCUSSION

**Where it reads:**

Regarding the educational aspect, although ELSA-Brazil consists of federal civil servants, 70% of the baseline cohort members had level of education of up to high school.

**It should read:**

Regarding the educational aspect, although ELSA-Brasil consists of federal civil servants, 70% of the baseline cohort members had level of education of up to high school.

**Where it reads:**

In this sense, we highlight the occupations related to the services of waste collection, cleaning, and conservation of public areas carried out by active workers participating in ELSA-Brazil.

**It should read:**

In this sense, we highlight the occupations related to the services of waste collection, cleaning, and conservation of public areas carried out by active workers participating in ELSA-Brasil.

**Where it reads:**

This is the first study to evaluate cancer mortality data in workers from the ELSA-Brazil cohort. Among the advantages of the adopted method, we can mention the possibility of a robust exploratory analysis, with a flexible and visually intuitive approach.

**It should read:**

This is the first study to evaluate cancer mortality data in workers from the ELSA-Brasil cohort. Among the advantages of the adopted method, we can mention the possibility of a robust exploratory analysis, with a flexible and visually intuitive approach.

**Where it reads:**

In addition, ELSA-Brazil is not a specific study for cancer, and the classification of deaths due to WRC presents challenges.

**It should read:**

In addition, ELSA-Brasil is not a specific study for cancer, and the classification of deaths due to WRC presents challenges.

**Where it reads:**

ACKNOWLEDGEMENTS: The authors would like to thank the ELSA-Brazil study team and participants for their important contributions.

**It should read:**

ACKNOWLEDGEMENTS: The authors would like to thank the ELSA-Brasil study team and participants for their important contributions.

**Where it reads:**

ETHICS COMMITTEE: The study results from the doctoral dissertation of the Graduate Program of the National School of Public Health, subarea of Epidemiology in Public Health, approved by the Research Ethics Committee, under number: 66407322.3.0000.5240, carried out with its own funding. ELSA-Brazil was funded by the Brazilian Ministry of Health (Department of Science and Technology) and the Brazilian Ministry of Science, Technology and Innovation (Funding agency of Studies and Projects and the National Council for Scientific and Technological Development, scholarship numbers: 01 06 0010.00 RS, 01 06 0212.00 BA, 01 06 0300.00 ES, 01 06 0278.00 MG, 01 06 0115.00 SP and 01 06 0071.00 RJ).

**It should read:**

ETHICS COMMITTEE: The study results from the doctoral dissertation of the Graduate Program of the National School of Public Health, subarea of Epidemiology in Public Health, approved by the Research Ethics Committee, under number: 66407322.3.0000.5240, carried out with its own funding. ELSA-Brasil was funded by the Brazilian Ministry of Health (Department of Science and Technology) and the Brazilian Ministry of Science, Technology and Innovation (Funding agency of Studies and Projects and the National Council for Scientific and Technological Development, scholarship numbers: 01 06 0010.00 RS, 01 06 0212.00 BA, 01 06 03

