# Supplementary material for: Occupational, socioeconomic factors and cancer mortality in participants of the Longitudinal Study of Adult Health (ELSA-Brazil): a multiple correspondence analysis
Source: Rev Bras Epidemiol. 2025 May 2;28:e250022. doi: 10.1590/1980-549720250022 (PMC12054984; doi:10.1590/1980-549720250022)
Supplement: Supplementary file 1 [file 1980-5497-rbepid-28-e250022-suppl.pdf]

Estudo Longitudinal de Saúde do Adulto ELSA BRASIL

MATERIAL SUPLEMENTAR

Criação da Variável Ocupação

Quadro S1- Variáveis disponíveis (banco):

Nome	Definição
Hoca20	Ativo ou Aposentado
Hoca03	Principais atividades no trabalho

QuadroS2 - Variáveis intermediárias:

Nome	Definição
cbo_atual1	Código CBO da ocupação atual
inativo	Código CBO da ocupação dos inativos

QuadroS3 -Variáveis derivadas:

Nome	Definição
A_ocupacao_ativo	Ocupação ativos
A_ocupacao_inativo	Ocupação inativos
A_ocupacao_todos	Ocupação ativos + inativos

Variáveis são códigos da ocupação agrupados em 4 a 6 dígitos segundo a Tabela da Classificação Brasileira de Ocupações CBO 2002 (MTE, 2010)

Quadro S4 – Classificação de Agentes quanto ao potencial de causar câncer (grau de carcinogenicidade) pelas Monografias da IARC, Volumes 1-133

GRUPO	CLASSIFICAÇÃO	Nº DE AGENTES
**1**	Carcinogênico para humanos	129
**2A**	Provavelmente carcinogênico para humanos	96
**2B**	Possivelmente carcinogênico para humanos	321
**3**	Não classificável quanto à sua carcinogenicidade para humanos	499

Fonte: Adaptado de IARC, 2024.

Quadro S5 - Classificação da variável ocupação ativos na população de estudo ELSA-Brasil, segundo exposições ocupacionais, a pelo menos 1 agente químico, físico ou biológico, ou circunstância de exposição com potencial de causar câncer (Grupo 1 e 2A), pertinentes ao exercício da ocupação.

Ocupações (Ativos)	Agente químico, físico, biológico e/ ou circunstâncias de exposição pertinentes a ocupação, classificado como carcinogênico ou provavelmente carcinogências para humanos	Grupo IARC	Categorização da Ocupação no ELSA-Brasil
Administradores	-	-	Não carcinogênica
Administradores de redes, sistemas e banco de dados	-	-	Não carcinogênica
Advogados	-	-	Não carcinogênica
Agentes comunitários de saúde e afins	Radiação solar	1	Carcinogênica
	Poluição ao ar livre	1	Carcinogênica
	Vírus (Hepatite B e C; Epstein Barr ;imunodeficiência humana tipo 1; linfotrópico humano de células T tipo I)	1	Carcinogênica
Agentes da saúde e do meio ambiente	Malathion	2A	Carcinogênica
	Radiação solar	1	Carcinogênica
	Poluição ao ar livre	1	Carcinogênica
Analistas de sistemas/suporte computacionais	-	-	Não carcinogênica
Arquitetos e urbanistas	-	-	Não carcinogênica
Arquivistas e museólogos	Dioxina	1	Carcinogênica
	Formaldeído	1	Carcinogênica
Artistas visuais, desenhistas industriais e conservadores-restauradores de bens culturais	Chumbo(tintas e pigmentos)	2A	Carcinogênica
	Pó de sílica	1	Carcinogênica
	Benzeno (tintas e vernizes)	1	Carcinogênica
Assistentes sociais e economistas domésticos	-	-	Não carcinogênica
Atletas profissionais	Radiação solar	1	Carcinogênica
Auxiliares de contabilidade	-	-	Não carcinogênica
Auxiliares de serviços de documentação, informação e pesquisa	Trabalho noturno	2A	Carcinogênica
Biólogos e afins<br>Biólogos e afins(continuação)	Radiação ionizante	1	Carcinogênica
	Bisclorometil éter	1	Carcinogênica
	Benzidina(substância utilizada em laboratórios)	1	Carcinogênica

	orto-toluidina (corante de tecidos biológicos usado em laboratórios)	1	Carcinogênica
Caixas e bilheteiros (exceto caixa de banco)	-	-	Não carcinogênica
Captadores de imagens em movimento	-	-	Não carcinogênica
Cirurgiões-dentistas	Formaldeído	1	Carcinogênica
	Radiação ionizante	1	Carcinogênica
	Benzidina	1	Carcinogênica
Contadores e afins	-	-	Não carcinogênica
Desenhistas técnicos e modelistas	-	-	Não carcinogênica
Diretores de espetáculos e afins	-	-	Não carcinogênica
Dirigentes das áreas de apoio da administração pública	-	-	Não carcinogênica
Economistas	-	-	Não carcinogênica
Encadernadores e recuperadores de livros (pequenos lotes ou a unidade)	Formaldeído	1	Carcinogênica
Enfermeiros	Trabalho noturno	2A	Carcinogênica
	Vírus (Hepatite B e C; Epstein Barr ;imunodeficiência humana tipo 1; linfotrópico humano de células T tipo I)	1	Carcinogênica
	MOPP e outros quimioterápicos	1	Carcinogênica
	Óxido de etileno	1	Carcinogênica
Engenheiros ambientais e afins	Radiação solar	1	Carcinogênica
	Poluição ao ar livre	1	Carcinogênica
Engenheiros civis e afins	Amianto	1	Carcinogênica
	Pó de sílica	1	Carcinogênica
	Poeira de madeira	1	Carcinogênica
Engenheiros de minas	Radônio	1	Carcinogênica
	Amianto	1	Carcinogênica
	Pó de sílica	1	Carcinogênica
Engenheiros eletricistas, eletrônicos e afins	Radiações ionizantes	1	Carcinogênica
Engenheiros mecânicos	Óxido de etileno	1	Carcinogênica

	Benzeno	1	Carcinogênica
Escriturários em geral, agentes, assistentes e auxiliares administrativos	-	-	Não carcinogênica
Farmacêuticos	Trabalho noturno	2A	Carcinogênica
	Ciclofosfamida	1	Carcinogênica
	Clorambucil	1	Carcinogênica
	MOPP e outros quimioterápicos	1	Carcinogênica
	Hidrazina	2A	Carcinogênica
Filólogos,tradutores ,intérpretes e afins	-	-	Não carcinogênica
Fisioterapeutas	Trabalho noturno	1	Carcinogênica
	Vírus (Hepatite B e C)	1	Carcinogênica
Físicos	Radiações ionizantes	1	Carcinogênica
Garçons, barmen, copeiros e sommeliers	Trabalho noturno	2A	Carcinogênica
Gerentes de produção e operações	-	-	Não carcinogênica
Gerentes de áreas de apoio	-	-	Não carcinogênica
Inspetores de alunos	-	-	Não carcinogênica
Instrutores e professores de cursos livres	-	-	Não carcinogênica
Leiloeiros e avaliadores	Amianto	1	Carcinogênica
	Pó de sílica	1	Carcinogênica
Locutores, comentaristas e repórteres de rádio e televisão	-	-	Não carcinogênica
Mecânicos de manutenção de máquinas industriais	chumbo (fumaça de solda)	2A	Carcinogênica
	cádmio (fumaça de solda)	1	Carcinogênica
Médicos	Trabalho noturno	2A	Carcinogênica
	MOPP e outros quimioterápicos	1	Carcinogênica
	Benzidina	1	Carcinogênica
	Vírus (Hepatite B e C; Epstein Barr ;imunodeficiência humana tipo 1; linfotrópico humano de células T tipo I)	1	Carcinogênica
Nutricionistas	-	-	Não carcinogênica
Operadores de equipamentos de entrada e	-	-	Não carcinogênica

transmissão de dados			
Operadores de rede de teleprocessamento e afins	Trabalho noturno	2A	Carcinogênica
Operadores de telefonia	-	-	Não carcinogênica
Operadores do comércio em lojas e mercados	-	-	Não carcinogênica
Porteiros e vigias	Trabalho noturno	2A	Carcinogênica
Produtores de espetáculos	-	-	Não carcinogênica
Professores de educação física	Radiação solar	1	Carcinogênica
Professores de nível médio na educação infantil	-	-	Não carcinogênica
Professores de nível superior do Ensino Fundamental (primeira a quarta séries)	-	-	Não carcinogênica
Professores de nível superior na educação infantil	-	-	Não carcinogênica
Professores do ensino médio	-	-	Não carcinogênica
Professores do ensino superior	-	-	Não carcinogênica
Professores e instrutores (com formação de nível superior) do ensino profissional	-	-	Não carcinogênica
Profissionais da informação	-	-	Não carcinogênica
Profissionais das ciências atmosféricas e espaciais e de astronomia	-	-	Não carcinogênica
Profissionais de administração ecônomico-financeira	-	-	Não carcinogênica
Profissionais de estatística	-	-	Não carcinogênica
Profissionais de recursos humanos	-	-	Não carcinogênica
Profissionais de relações públicas, publicidade, mercado e negócios	-	-	Não carcinogênica
Profissionais do jornalismo	-	-	Não carcinogênica
Profissionais em pesquisa e análise geográfica	-	-	Não carcinogênica
Programadores, avaliadores e orientadores de ensino	-	-	Não carcinogênica
Psicólogos e psicanalistas	-	-	Não carcinogênica
Químicos	Cloreto de vinila	1	Carcinogênica
	Cádmio e seus compostos	1	Carcinogênica

	Ácido sulfúrico	1	Carcinogênica
Recepcionistas	-	-	Não carcinogênica
Supervisores administrativos	-	-	Não carcinogênica
Supervisores da construção civil	Asbestos (todas as formas de Asbestos/Amianto)	1	Carcinogênica
	Benzeno	1	Carcinogênica
	Poeira de sílica, sílica cristalina na forma de quartzo ou cristobolita	1	Carcinogênica
	Radiação solar	1	Carcinogênica
Supervisores das artes gráficas	-	-	Não carcinogênica
Supervisores de serviços financeiros, de câmbio e de controle	-	-	Não carcinogênica
Trabalhadores da pré-impressão gráfica	Formaldeído	1	Carcinogênica
	Hidrazina	2A	Carcinogênica
Trabalhadores de instalações elétricas	Radiação ultravioleta (processo de soldagem)	1	Carcinogênica
Trabalhadores do acabamento gráfico	Formaldeído	1	Carcinogênica
Trabalhadores nos serviços de administração de edifícios	Trabalho noturno	1	Carcinogênica
Trabalhadores nos serviços de coleta de resíduos, de limpeza e conservação de áreas públicas	Poeiras de madeira (geradas durante o processo de lixamento e corte de madeira)	1	Carcinogênica
	Asbesto (Amianto)	1	Carcinogênica
	Benzeno	1	Carcinogênica
	Poeiras de sílica cristalina (geradas durante a limpeza e conservação de áreas com pisos de concreto ou pedra)	1	Carcinogênica
	Fumos de diesel (gerados pelos veículos usados no serviço de coleta de resíduos)	2A	Carcinogênica
	Radiação solar	1	Carcinogênica
Trabalhadores nos serviços de embelezamento e higiene	Formaldeído	1	Carcinogênica
	Aminobifenil	1	Carcinogênica
Técnico em farmácia e em manipulação farmacêutica	Trabalho noturno	2A	Carcinogênica
	MOPP e outros quimioterápicos	1	Carcinogênica
	Hidrazina	2A	Carcinogênica

Técnicos agropecuários	Agrotóxicos (glifosato, malathion)	2A	Carcinogênica
	Agrotóxico (lindano)	1	Carcinogênica
	Radiação solar	1	Carcinogênica
Técnicos de controle da produção	-	-	Não carcinogênica
Técnicos de desenvolvimento de sistemas e aplicações	-	-	Não carcinogênica
Técnicos de odontologia	Benzina	1	Carcinogênica
	Óxido de etileno	1	Carcinogênica
Técnicos e auxiliares de enfermagem	Trabalho noturno	2A	Carcinogênica
	MOPP e outros quimioterápicos; Radiação Ionizante	1	Carcinogênica
	Vírus (Hepatite B e C; Epstein Barr ;imunodeficiência humana tipo 1; linfotrópico humano de células T tipo I)	1	Carcinogênica
	Óxido de etileno	1	Carcinogênica
Técnicos e auxiliares técnicos em patologia clínica	Trabalho noturno	2A	Carcinogênica
	Vírus (Hepatite B e C; Epstein Barr ;imunodeficiência humana tipo 1; linfotrópico humano de células T tipo I)	1	Carcinogênica
Técnicos em biblioteconomia	-	-	Não Carcinogênica
Técnicos em biologia	Benzidina	1	Carcinogênica
	Bisclorometil éter	1	Carcinogênica
	Vírus (Hepatite B e C; Epstein Barr ;imunodeficiência humana tipo 1; linfotrópico humano de células T tipo I)	2A	Carcinogênica
	MOPP e outros quimioterápicos	1	Carcinogênica
Técnicos em calibração e instrumentação		-	Não carcinogênica
Técnicos em cenografia	Poeiras de madeira (geradas durante o processo de lixamento e corte de madeira)	1	Carcinogênica
	Benzeno	1	Carcinogênica
	Formaldeído	1	Carcinogênica
Técnicos em construção civil (edificações)	Asbestos (todas as formas de Asbestos/Amianto)	1	Carcinogênica
	Benzeno	1	Carcinogênica
	Radiação ultravioleta (processo de soldagem)	1	Carcinogênica

	Poeira de sílica, sílica cristalina na forma de quartzo ou cristobolita	1	Carcinogênica
Técnicos em construção civil (obras de infraestrutura)	Asbestos (todas as formas de Asbestos/Amianto)	1	Carcinogênica
	Benzeno	1	Carcinogênica
	Radiação ultravioleta (processo de soldagem)	1	Carcinogênica
	Poeira de sílica, sílica cristalina na forma de quartzo ou cristobolita	1	Carcinogênica
Técnicos em contabilidade	-	-	Não carcinogênica
Técnicos em controle ambiental, utilidades e tratamento de efluentes	Benzeno	1	Carcinogênica
	Radiação solar	1	
	Trabalho Noturno	2A	
	Chumbo	2A	Carcinogênica
	Cádmio	1	Carcinogênica
Técnicos em eletricidade e eletrotécnica	Amianto (isolamentos elétricos e outros materiais de construção.)	1	Carcinogênica
Técnicos em eletrônica	Benzeno	1	Carcinogênica
	Chumbo	2A	Carcinogênica
	Cádmio	1	Carcinogênica
Técnicos em equipamentos médicos e odontológicos	Radiação ionizante	1	Carcinogênica
	Níquel e seus compostos	1	Carcinogênica
Técnicos em fotônica	-	-	Não carcinogênica
Técnicos em geomática	-	-	Não carcinogênica
Técnicos em mecânica veicular	Benzeno	1	Carcinogênica
	Chumbo (fumaça de solda)	2A	Carcinogênica
	Cádmio (fumaça de solda)	1	Carcinogênica
Técnicos em operação de sistemas de televisão e de produtoras de vídeo	-	-	Não carcinogênica
Técnicos em produção, conservação e de qualidade de alimentos	Glifosato	2A	Carcinogênica
	Radiação ionizante	1	Carcinogênica
	Aflatoxina	1	Carcinogênica

Técnicos em próteses ortopédicas	-	-	Não carcinogênica
Técnicos em segurança no trabalho	-	-	Não carcinogênica
Técnicos florestais	-	-	Não carcinogênica
Técnicos mecânicos (ferramentas)	Benzeno	1	Carcinogênica
Técnicos mecânicos na manutenção de máquinas, sistemas e instrumentos	Benzeno	1	Carcinogênica
Técnicos químicos	Cloreto de vinila	1	Carcinogênica
	Cádmio e seus compostos	1	Carcinogênica
	Benzeno	1	Carcinogênica
	Ácido sulfúrico	1	Carcinogênica
	Tricloroeetileno	1	Carcinogênica
Veterinários e zootecnistas	Agrotóxico (DDT)	2A	Carcinogênica
	Vírus da imunodeficiência humana tipo 1	1	Carcinogênica
	Benzidina	1	Carcinogênica
Vigilantes e guardas de segurança	Trabalho noturno	2A	Carcinogênica

Quadro S6 - Classificação das causas de morte por câncer na população de estudo ELSA- Brasil, em "Óbitos por Câncer Relacionados ao Trabalho" a partir das tipologias de câncer associadas a pelo menos dois agentes ou circunstâncias de exposição, listados nos Grupos 1 da IARC.

**Tipologia dos óbitos por câncer que ocorreram no ELSA- Brasil**	**Agentes previstos no Grupo1 da IARC que podem estar presentes no ambiente de trabalho**	**Classificação**
**Pulmão**	exposição ocupacional associada a processo de Acheson	Óbito por câncer relacionado ao trabalho
	produção de alumínio	
	arsênico e seus compostos inorgânico	
	todas as formas de asbestos	
	berílio e seus compostos	
	biclorometil éter, clorometil-metil-éter	
	cádmio e seus compostos	
	compostos de cromo (VI)	
	gaseificação de carvão	
	piche de alcatrão de carvão	
	produção de coque	
	escape de motor a diesel	
	mineração subterrânea de hematita	
	exposição ocupacional durante o processo de fundição de ferro e aço	
	MOPP e outras quimioterapias combinadas, incluindo agentes alquilantes	
	compostos de níquel	
	poluição ao ar livre	
	exposição ocupacional como pintor	
	plutônio	
	radônio-222 e seus produtos de decomposição	
	indústria de fabricação de borracha	
	poeira de sílica, sílica cristalina na forma de quartzo ou cristobolita	
	fuligem	
	tabagismo passivo (tabaco)	
	fumos de soldagem	
	radiação x e gama	
Mama	radiação x gama	Óbito por câncer Não relacionado ao trabalho
Cólon e reto	radiação x gama	Óbito por câncer Não relacionado ao trabalho
**Fígado**	aflatoxinas	Óbito por câncer relacionado ao trabalho
	vírus da hepatite B (infecção crônica)	
	vírus da hepatite C (infecção crônica)	
	plutônio	

	tório-232 e seus produtos de decomposição	
	cloreto de vinila	
**Estômago**	radiação x e gama	Óbito por câncer relacionado ao trabalho
	indústria de fabricação de borracha	
**Bexiga**	produção de alumínio	Óbito por câncer relacionado ao trabalho
	4-aminobifenila	
	arsênico e seus compostos inorgânicos	
	produção de auramina	
	benzidina	
	ciclofosfamida	
	exposição ocupacional como bombeiro	
	produção magenta	
	2-naftilamina	
	exposição ocupacional como pintor	
	indústria de fabricação de borracha	
	orto-toluidina	
	radiação x e gama	
**Leucemia**	benzeno	Óbito por câncer relacionado ao trabalho
	formaldeído	
	MOPP e outras quimioterapias combinadas, incluindo agentes alquilantes	
	fósforo-32	
	tório-232	
	radiação X e Gama	
	1,3-butadieno	
	indústria de fabricação de borracha	
**Rim**	tricloroetileno	Óbito por câncer relacionado ao trabalho
	radiação x gama	
Cérebro e SNC	radiação x gama	Óbito por câncer Não relacionado ao trabalho
**Colo uterino e Ovário**	todas as formas de asbestos	Óbito por câncer Não relacionado ao trabalho
	Infecção pelo HIV tipo 1	Óbito por câncer relacionado ao trabalho
	Papilomavírus humano tipos 16,18,31,33,35,39,45,51,52,56,58 e 59	
**Mieloma**	1,3-Butadieno	Óbito por câncer relacionado ao trabalho
	pentaclorofenol	
**Linfoma**	vírus de *Epstein Barr*	Óbito por câncer relacionado ao trabalho
	infecção pelo HIV tipo 1	
	azatioprina	
	ciclosporina	
	vírus da hepatite C (infecção crônica)	

	lindano<br>pentaclorofenol	
**Aerodigestivo alto:**	-	Óbito por câncer Não relacionado ao trabalho
**Próstata**	-	Óbito por câncer Não relacionado ao trabalho
**Pâncreas**	-	Óbito por câncer Não relacionado ao trabalho
**Outros Cânceres**	-	Óbito por câncer Não relacionado ao trabalho

Descrição detalhada das Ocupações dos trabalhadores ativos segundo a codificação da CBO 2002, utilizando o nível máximo de desagregação de 6 dígitos.

- ## CBO 252105 - Administrador

Descrição Sumária:

Planejam, organizam, controlam e assessoram as organizações nas áreas de recursos humanos, patrimônio, materiais, informações, financeira, tecnológica, entre outras; implementam programas e projetos; elaboram planejamento organizacional; promovem estudos de racionalização e controlam o desempenho organizacional. Prestam consultoria administrativa a organizações e pessoas.

Formação e Experiência:

Para o exercício dessa ocupação requer-se curso superior completo em Administração de empresas ou Administração pública, com registro no Conselho Regional de Administração (CRA).

Condições Gerais de Exercício:

Trabalham em qualquer ramo de atividade econômica, serviços, comércio e indústria, incluindo-se a administração pública. São assalariados celetistas estatutários ou autônomos. Geralmente, trabalham em equipe, em ambiente fechado e em horário diurno. Estão sujeitos a pressão por cumprimento de prazos e metas.

- ## CBO 212310 - Administrador de redes
- ### Descrição Sumária:

Administram ambientes computacionais, implantando e documentando rotinas e projetos e controlando os níveis de serviço de sistemas operacionais, banco de dados e redes. Fornecem suporte técnico no uso de equipamentos e programas computacionais e no apoio a usuários, configuram e instalam recursos e sistemas computacionais, controlam a segurança do ambiente computacional.

- ### Formação e Experiência:

Para o exercício profissional dessas ocupações, requer-se curso superior completo, em nível de bacharelado ou tecnologia. Podem, também, obter formação específica por meio de cursos de qualificação, com carga horária entre duzentas e quatrocentas horas. A experiência profissional prévia requerida dos titulares para o exercício pleno das atividades é de um a dois anos, exceto para

o Administrador de Bancos de Dados, que é de aproximadamente quatro anos, em todos os casos incluindo o tempo de estágio.

- ## Condições Gerais de Exercício:

Ao projetar e desenvolver sistemas computacionais, trabalham tanto na área de software quanto na área de hardware, incluindo robótica. Podem ser encontrados, por exemplo, em indústrias químicas, de material de transporte, de máquinas para escritórios e equipamentos de informática, em instituições financeiras e nas telecomunicações. Costumam desenvolver suas atividades em universidades, institutos de pesquisa, grandes empresas, tanto no setor público como no privado, em ambientes, nos quais se pesquisa tecnologia avançada para ser absorvida pelo mercado. Podem trabalhar como empregados, funcionários públicos ou como autônomos sob supervisão ocasional, desenvolvendo seus trabalhos em equipe multidisciplinar, parceria ou cooperação, de forma presencial e também a distância.

- ## CBO 212315 - Administrador de sistemas operacionais
- ## Descrição Sumária:

Administram ambientes computacionais, implantando e documentando rotinas e projetos e controlando os níveis de serviço de sistemas operacionais, banco de dados e redes. Fornecem suporte técnico no uso de equipamentos e programas computacionais e no apoio a usuários, configuram e instalam recursos e sistemas computacionais, controlam a segurança do ambiente computacional.

- ## Formação e Experiência:

Para o exercício profissional dessas ocupações, requer-se curso superior completo, em nível de bacharelado ou tecnologia. Podem, também, obter formação específica por meio de cursos de qualificação, com carga horária entre duzentas e quatrocentas horas. A experiência profissional prévia requerida dos titulares para o exercício pleno das atividades é de um a dois anos, exceto para o Administrador de Bancos de Dados, que é de aproximadamente quatro anos, em todos os casos incluindo o tempo de estágio.

- ## Condições Gerais de Exercício:

Ao projetar e desenvolver sistemas computacionais, trabalham tanto na área de software quanto na área de hardware, incluindo robótica. Podem ser encontrados, por exemplo, em indústrias químicas, de material de transporte, de máquinas para escritórios e equipamentos de informática, em instituições financeiras e nas telecomunicações. Costumam desenvolver suas atividades em universidades, institutos de pesquisa, grandes empresas, tanto no setor público como no privado, em ambientes, nos quais se pesquisa tecnologia avançada para ser absorvida pelo mercado. Podem trabalhar como empregados, funcionários públicos ou como autônomos sob supervisão ocasional, desenvolvendo seus trabalhos em equipe multidisciplinar, parceria ou cooperação, de forma presencial e também a distância.

- ## CBO 212305 - Administrador de banco de dados
- ## Descrição Sumária:

Administram ambientes computacionais, implantando e documentando rotinas e projetos e controlando os níveis de serviço de sistemas operacionais, banco de dados e redes. Fornecem suporte técnico no uso de equipamentos e programas computacionais e no apoio a usuários, configuram e instalam recursos e sistemas computacionais, controlam a segurança do ambiente computacional.

- ## Formação e Experiência:

Para o exercício profissional dessas ocupações, requer-se curso superior completo, em nível de bacharelado ou tecnologia. Podem, também, obter formação específica por meio de cursos de qualificação, com carga horária entre duzentas e quatrocentas horas. A experiência profissional prévia requerida dos titulares para o exercício pleno das atividades é de um a dois anos, exceto para o Administrador de Bancos de Dados, que é de aproximadamente quatro anos, em todos os casos incluindo o tempo de estágio.

- ## Condições Gerais de Exercício:

Ao projetar e desenvolver sistemas computacionais, trabalham tanto na área de software quanto na área de hardware, incluindo robótica. Podem ser encontrados, por exemplo, em indústrias químicas, de material de transporte, de máquinas para escritórios e equipamentos de informática, em instituições financeiras e nas telecomunicações. Costumam desenvolver suas atividades em universidades, institutos de pesquisa, grandes empresas, tanto no setor público como no privado, em ambientes, nos quais se pesquisa tecnologia avançada para ser absorvida pelo mercado. Podem trabalhar como empregados, funcionários públicos ou como autônomos sob supervisão ocasional, desenvolvendo seus trabalhos em equipe multidisciplinar, parceria ou cooperação, de forma presencial e também a distância.

- # CBO 241005 - Advogado
- ## Descrição Sumária:

Postulam, em nome do cliente, em juízo, propondo ou contestando ações, solicitando providências ao magistrado ou ministério público, avaliando provas documentais e orais, realizando audiências trabalhistas, penais comuns e cíveis, instruindo a parte e atuando no tribunal de júri, e extrajudicialmente, mediando questões, contribuindo na elaboração de projetos de lei, analisando legislação para atualização e implementação, assistindo empresas, pessoas e entidades, assessorando negociações internacionais e nacionais; zelam pelos interesses do cliente na manutenção e integridade dos seus bens, facilitando negócios, preservando interesses individuais e coletivos, dentros dos princípios éticos e de forma a fortalecer o estado democrático de direito.

- ## Formação e Experiência:

O exercício dessas ocupações requer ensino superior completo e o exame da OAB do estado de domicílio civil do bacharel em direito.

- ## Condições Gerais de Exercício:

Trabalham em escritórios de advogacia, em empresas agrícolas, comerciais, industriais, serviços e na administração pública, como estatutários, assalariados ou autônomos. Executam suas funções

sem supervisão, em ambiente fechado e em horário diurno. Eventualmente, trabalham sob pressão, levando à situação de estresse.

CBO 515105 - Agente comunitário de saúde

Descrição Sumária

Visitam domicílios periodicamente; assistem pacientes, dispensando-lhes cuidados sim ples de saúde, sob orientação e supervisão de profissionais da saúde; orientam a comunidade para promoção da saúde; rastreiam focos de doenças específicas; realizam partos; promovem educação sanitária e ambiental; participam de campanhas preventivas; incentivam atividades comunitárias; promovem comunicação entre unidade de saúde, autoridades e comunidade; realizam manutenção dos sistemas de abastecimento de água e executam tarefas administrativas.

Formação e Experiência

O exercício profissional requer ensino fundamental, além de curso profissionalizante com duração de duzentas a quatrocentas horas/aula. o ensino fundamental também é desejável para o agente indígena de saúde e agente indígena de saneamento, que muitas vezes, dependendo da região ou da distância de centros urbanos, não possuem nenhuma escolaridade formal. os profissionais da saúde indígena são preparados concursos profissionalizantes com carga horária acima de quatrocentas horas/aula. a principal característica do agente comunitário de saúde, do visitador sanitário, do agente indígena de saúde e do agente indígena de saneamento é a capacidade de relações interpessoais, mobilizada no trabalho de orientação junto à comunidade, no que se refere à saúde e prevenção de doenças. a(s) ocupação(ões) elencada(s) nesta família ocupacional demanda formação profissional para efeitos do cálculo do número de aprendizes a serem contratados pelos estabelecimentos, nos termos do artigo 429da consolidação das leis do trabalho - clt, exceto os casos previstos no art. 10 do decreto 5. 598/2005.

Condições Gerais de Exercício

Em sua maioria, são empregados formais com carteira assinados, ou autônomos que atuam no ramo da saúde e serviço social. Trabalham em equipe, sob supervisão permanente em horários **diurnos e em rodízio de turnos. Trabalham em local fechado ou a céu aberto,** dependendo da necessidade. Frequentemente são expostos às variações de temperatura, **materiais tóxicos**, **doenças contagiosas e risco de acidentes com materiais perfuro cortantes.**

CBO 3522 - Agentes da saúde e do meio ambiente

Descrição Sumária

Orientam e fiscalizam as atividades e obras para prevenção/preservação ambiental da saúde, por meio de vistorias, inspeções e análises técnicas de locais, atividades, obras, projetos e processos, visando o cumprimento da legislação ambiental e sanitária; promovem educação sanitária e ambiental.

Formação e Experiência

A escolaridade para ocupar esses empregos/ocupações varia do ensino médio ao ensino superior, incompleto ou completo, de várias áreas do conhecimento como: biologia, engenharia, etc., além de curso básico de qualificação de até duzentas horas/aula, não sendo exigida experiência profissional. a(s) ocupação(ões) elencada(s)nesta família ocupacional demanda formação profissional para efeitos do cálculo do número de aprendizes a serem contratados pelos estabelecimentos, nos termos do artigo 429 da consolidação das leis do trabalho - clt, exceto os casos previstos no art. 10 do decreto 5.598/2005.

Condições Gerais de Exercício

Esses profissionais atuam, predominantemente, nas áreas ligadas à agricultura, pecuária, silvicultura, exploração florestal, pesca, aquicultura e serviços relacionados. São empregados com carteira e trabalham em equipe, como agente ambiental ou agente de saúde pública, com supervisão permanente. Realizam seus trabalhos em ambientes fechados**, a céu aberto ou em veículos**, em horário diurno, podendo, eventualmente, trabalhar em horários irregulares. Podem trabalhar sob pressão, levando à situação de estresse e em posições desconfortáveis durante longos períodos, no caso do agente ambiental; às vezes são expostos a ruídos intensos, temperaturas extremas e riscos de integridade física.

- **CBO 212420 - Analista de suporte computacional**
- **Descrição Sumária:**

Desenvolvem e implantam sistemas informatizados dimensionando requisitos e funcionalidade dos sistemas, especificando sua arquitetura, escolhendo ferramentas de desenvolvimento, especificando programas, codificando aplicativos. Administram ambiente informatizado, prestam suporte técnico ao cliente, elaboram documentação técnica. Estabelecem padrões, coordenam projetos, oferecem soluções para ambientes informatizados e pesquisam tecnologias em informática.

- **Formação e Experiência:**

Para o exercício profissional dessas ocupações, requer-se curso superior completo, em nível de bacharelado ou tecnologia. Podem, também, obter formação específica por meio de cursos de qualificação, com carga horária entre duzentas e quatrocentas horas. A experiência profissional prévia requerida dos titulares para o exercício pleno das atividades é de um a dois anos, incluindo o tempo de estágio. Em função da inovação tecnológica, a permanência no mercado de trabalho requer atualização contínua dos profissionais.

- **Condições Gerais de Exercício:**

Exercem suas atividades em qualquer setor da atividade econômica, tais como a indústria, o comércio, os serviços, a agropecuária ou a administração pública. Podem trabalhar em empresas públicas ou privadas, em geral de médio e grande porte. Seu trabalho se desenvolve majoritariamente em equipe, de forma cooperativa, com supervisão ocasional. Não há predominância de um tipo de vínculo de trabalho: os profissionais podem ser assalariados ou trabalhador por conta própria e trabalham em período diurno.

- **CBO 214125 - Arquiteto urbanista**

- ## Descrição Sumária:

Elaboram planos e projetos associados à arquitetura em todas as suas etapas, definindo materiais, acabamentos, técnicas, metodologias, analisando dados e informações. Fiscalizam e executam obras e serviços, desenvolvem estudos de viabilidade financeira, econômica, ambiental. Podem prestar serviços de consultoria e assessoramento, bem como estabelecer políticas de gestão.

- ## Formação e Experiência:

Para o exercício das ocupações exige-se o curso superior completo em arquitetura e urbanismo, com ocorrência de profissionais com cursos de especialização e/ou pós-graduação.

- ## Condições Gerais de Exercício:

O trabalho é exercido em atividades econômicas como a da construção civil, de empresas imobiliárias, industriais e de serviços, na condição de empregado ou autônomo, prestando serviços. São mais frequentemente encontrados em empresas e escritórios de médio e grande porte do setor privado, em empresas e órgãos administrativos do setor público, em institutos de pesquisa e planejamento urbano, em instituições ligadas ao patrimônio histórico e na área ambiental. Seu trabalho se desenvolve tanto de forma individual como integrando equipe de trabalho especializada ou multidisciplinar.

- # CBO 261305 - Arquivista
- ## Descrição Sumária:

Organizam documentação de arquivos institucionais e pessoais, criam projetos de museus e exposições, organizam acervos museológicos públicos e privados. Dão acesso à informação, conservam acervos. Preparam ações educativas ou culturais, planejam e realizam atividades técnico-administrativas, orientam implantação das atividades técnicas. Participam da política de criação e implantação de museus e instituições arquivísticas.

- ## Formação e Experiência:

As ocupações da família requerem curso superior completo na área. Não é incomum, contudo a presença de profissionais com cursos de especialização ou mesmo pós-graduação.

- ## Condições Gerais de Exercício:

Os profissionais podem trabalhar em museus públicos ou particulares, em arquivos oficiais dos estados, municípios ou universidades, em centros de documentação vinculados a empresas ou instituições públicas ou privadas, no ensino, etc. Desenvolvem suas atividades em equipes com supervisão ocasional, como empregados registrados ou como autônomos. Em algumas atividades, alguns profissionais podem estar sujeitos aos efeitos da **exposição a materiais tóxicos e a micro-organismos.**

-
- # CBO 261310 - Museólogo

- **Descrição Sumária:**

Organizam documentação de arquivos institucionais e pessoais, criam projetos de museus e exposições, organizam acervos museológicos públicos e privados. Dão acesso à informação, conservam acervos. Preparam ações educativas ou culturais, planejam e realizam atividades técnico-administrativas, orientam implantação das atividades técnicas. Participam da política de criação e implantação de museus e instituições arquivísticas.

- **Formação e Experiência:**

As ocupações da família requerem curso superior completo na área. Não é incomum, contudo a presença de profissionais com cursos de especialização ou mesmo pós-graduação.

- **Condições Gerais de Exercício:**

Os profissionais podem trabalhar em museus públicos ou particulares, em arquivos oficiais dos estados, municípios ou universidades, em centros de documentação vinculados a empresas ou instituições públicas ou privadas, no ensino, etc. Desenvolvem suas atividades em equipes com supervisão ocasional, como empregados registrados ou como autônomos. Em algumas atividades, alguns profissionais podem estar sujeitos aos efeitos da exposição a **materiais tóxicos e a micro-organismos.**

- **CBO 262405 - Artista (artes visuais)**
- **Descrição Sumária:**

Concebem e desenvolvem obras de arte e projetos de design, elaboram e executam projetos de restauração e conservação preventiva de bens culturais móveis e integrados. Para tanto realizam pesquisas, elaboram propostas e divulgam suas obras de arte, produtos e serviços.

- **Formação e Experiência:**

A formação requerida para os desenhistas industriais de produto, gráficos e de produto de moda, também conhecidos como designers, é o curso superior de Tecnologia na área ou bacharelado. No caso dos artistas visuais e dos conservadores-restauradores, a escolaridade não é requisito imprescindível. Há também profissionais de notório saber. Registra-se tendência de profissionalização na área das artes, sendo desejável qualificação formal ou informal. O desempenho pleno das atividades, para os artistas visuais, conservadores-restauradores, desenhistas industriais de produto e desenhistas industriais gráficos, ocorre com cinco anos de experiência profissional, enquanto que para os desenhistas industriais de produto de moda ocorre entre três e quatro anos.

- **Condições Gerais de Exercício:**

Os artistas visuais e os desenhistas industriais de produto, gráficos e de produto de moda têm em comum o processo de criação e a utilização de conjuntos de técnicas específicas às respectivas áreas de atuação. Diferem, basicamente, quanto à finalidade do trabalho: enquanto os artistas visuais criam e produzem peças únicas ou com tiragem limitada, em resposta a uma encomenda ou não, os

desenhistas industriais respondem sempre a uma encomenda e estão voltados para a concepção de peças que serão produzidas em série. Já os conservadores-restauradores utilizam um conjunto de técnicas específicas para restauração de bens culturais. A forma de trabalhar também apresenta semelhanças e diferenças, a saber: os desenhistas industriais de produto, gráficos e de produto de moda e os conservadores-restauradores trabalham com supervisão ocasional enquanto os artistas visuais trabalham sem supervisão e em horários irregulares. Em todas as ocupações predominam os autônomos. Os artistas visuais trabalham de forma individual enquanto os desenhistas industriais em equipe multidisciplinar e os conservadores-restauradores podem trabalhar tanto individualmente como em equipe. No exercício de algumas atividades, os artistas visuais e os conservadores-restauradores podem trabalhar em posições desconfortáveis. Todas as ocupações estão sujeitas à exposição a **materiais tóxicos**.

- ## CBO 262410 - Desenhista industrial gráfico (designer gráfico)
- ## Descrição Sumária:

Concebem e desenvolvem obras de arte e projetos de design, elaboram e executam projetos de restauração e conservação preventiva de bens culturais móveis e integrados. Para tanto realizam pesquisas, elaboram propostas e divulgam suas obras de arte, produtos e serviços.

- ## Formação e Experiência:

A formação requerida para os desenhistas industriais de produto, gráficos e de produto de moda, também conhecidos como designers, é o curso superior de Tecnologia na área ou bacharelado. No caso dos artistas visuais e dos conservadores-restauradores, a escolaridade não é requisito imprescindível. Há também profissionais de notório saber. Registra-se tendência de profissionalização na área das artes, sendo desejável qualificação formal ou informal. O desempenho pleno das atividades, para os artistas visuais, conservadores-restauradores, desenhistas industriais de produto e desenhistas industriais gráficos, ocorre com cinco anos de experiência profissional, enquanto que para os desenhistas industriais de produto de moda ocorre entre três e quatro anos.

- ## Condições Gerais de Exercício:

Os artistas visuais e os desenhistas industriais de produto, gráficos e de produto de moda têm em comum o processo de criação e a utilização de conjuntos de técnicas específicas às respectivas áreas de atuação. Diferem, basicamente, quanto à finalidade do trabalho: enquanto os artistas visuais criam e produzem peças únicas ou com tiragem limitada, em resposta a uma encomenda ou não, os desenhistas industriais respondem sempre a uma encomenda e estão voltados para a concepção de peças que serão produzidas em série. Já os conservadores-restauradores utilizam um conjunto de técnicas específicas para restauração de bens culturais. A forma de trabalhar também apresenta semelhanças e diferenças, a saber: os desenhistas industriais de produto, gráficos e de produto de moda e os conservadores-restauradores trabalham com supervisão ocasional enquanto os artistas visuais trabalham sem supervisão e em horários irregulares. Em todas as ocupações predominam os autônomos. Os artistas visuais trabalham de forma individual enquanto os desenhistas industriais em equipe multidisciplinar e os conservadores-restauradores podem trabalhar tanto individualmente como em equipe. No exercício de algumas atividades, os artistas visuais e os conservadores-restauradores podem trabalhar em posições desconfortáveis. Todas as ocupações estão sujeitas à **exposição a materiais tóxicos.**

-

- **CBO 262415 - Conservador-restaurador de bens culturais**
- **Descrição Sumária:**

Concebem e desenvolvem obras de arte e projetos de design, elaboram e executam projetos de restauração e conservação preventiva de bens culturais móveis e integrados. Para tanto realizam pesquisas, elaboram propostas e divulgam suas obras de arte, produtos e serviços.

- **Formação e Experiência:**

A formação requerida para os desenhistas industriais de produto, gráficos e de produto de moda, também conhecidos como designers, é o curso superior de Tecnologia na área ou bacharelado. No caso dos artistas visuais e dos conservadores-restauradores, a escolaridade não é requisito imprescindível. Há também profissionais de notório saber. Registra-se tendência de profissionalização na área das artes, sendo desejável qualificação formal ou informal. O desempenho pleno das atividades, para os artistas visuais, conservadores-restauradores, desenhistas industriais de produto e desenhistas industriais gráficos, ocorre com cinco anos de experiência profissional, enquanto que para os desenhistas industriais de produto de moda ocorre entre três e quatro anos.

- **Condições Gerais de Exercício:**

Os artistas visuais e os desenhistas industriais de produto, gráficos e de produto de moda têm em comum o processo de criação e a utilização de conjuntos de técnicas específicas às respectivas áreas de atuação. Diferem, basicamente, quanto à finalidade do trabalho: enquanto os artistas visuais criam e produzem peças únicas ou com tiragem limitada, em resposta a uma encomenda ou não, os desenhistas industriais respondem sempre a uma encomenda e estão voltados para a concepção de peças que serão produzidas em série. Já os conservadores-restauradores utilizam um conjunto de técnicas específicas para restauração de bens culturais. A forma de trabalhar também apresenta semelhanças e diferenças, a saber: os desenhistas industriais de produto, gráficos e de produto de moda e os conservadores-restauradores trabalham com supervisão ocasional enquanto os artistas visuais trabalham sem supervisão e em horários irregulares. Em todas as ocupações predominam os autônomos. Os artistas visuais trabalham de forma individual enquanto os desenhistas industriais em equipe multidisciplinar e os conservadores-restauradores podem trabalhar tanto individualmente como em equipe. No exercício de algumas atividades, os artistas visuais e os conservadores-restauradores podem trabalhar em posições desconfortáveis. Todas as ocupações estão sujeitas à **exposição a materiais tóxicos**.

-
- **CBO 251605 - Assistente social**
- **Descrição Sumária:**

Prestam serviços sociais orientando indivíduos, famílias, comunidade e instituições sobre direitos e deveres (normas, códigos e legislação), serviços e recursos sociais e programas de educação; planejam, coordenam e avaliam planos, programas e projetos sociais em diferentes áreas de atuação profissional (seguridade, educação, trabalho, jurídica, habitação e outras), atuando nas esferas pública e privada; orientam e monitoram ações em desenvolvimento relacionadas à economia doméstica, nas áreas de habitação, vestuário e têxteis, desenvolvimento humano, economia familiar, educação do consumidor, alimentação e saúde; desempenham tarefas administrativas e articulam recursos financeiros disponíveis.

- **Formação e Experiência:**

O exercicio dessas ocupações requer curso superior em serviço social para a ocupação de assistente social e formação em Economia Doméstica para a ocupação de economista doméstico.

- **Condições Gerais de Exercício:**

Trabalham em instituições das esferas pública e privada, bem como em ONG. Podem atuar em empresas ou instituições do setor agropecuário, comercial, industrial e de serviços. O foco de atuação é a família (ou indíviduo). São estatutários ou empregados com carteira assinada. Trabalham em equipe, sob supervisão ocasional, em ambientes fechados e em horário diurno, podendo, o assistente social trabalhar em horários irregulares durante plantões e em casos emergenciais. Eventualmente, trabalham sob pressão, levando à situação de estresse.

- **CBO 251610 - Economista doméstico**
- **Descrição Sumária:**

Prestam serviços sociais orientando indivíduos, famílias, comunidade e instituições sobre direitos e deveres (normas, códigos e legislação), serviços e recursos sociais e programas de educação; planejam, coordenam e avaliam planos, programas e projetos sociais em diferentes áreas de atuação profissional (seguridade, educação, trabalho, jurídica, habitação e outras), atuando nas esferas pública e privada; orientam e monitoram ações em desenvolvimento relacionados à economia doméstica, nas áreas de habitação, vestuário e têxteis, desenvolvimento humano, economia familiar, educação do consumidor, alimentação e saúde; desempenham tarefas administrativas e articulam recursos financeiros disponíveis.

- **Formação e Experiência:**

O exercicio dessas ocupações requer curso superior em serviço social para a ocupação de assistente social e formação em Economia Doméstica para a ocupação de economista doméstico.

- **Condições Gerais de Exercício:**

Trabalham em instituições das esferas pública e privada, bem como em ONG. Podem atuar em empresas ou instituições do setor agropecuário, comercial, industrial e de serviços. O foco de atuação é a família (ou indíviduo). São estatutários ou empregados com carteira assinada. Trabalham em equipe, sob supervisão ocasional, em ambientes fechados e em horário diurno, podendo, o assistente social trabalhar em horários irregulares durante plantões e em casos emergenciais. Eventualmente, trabalham sob pressão, levando à situação de estresse.

- **CBO 377105 - Atleta profissional (outras modalidades)**
- **Descrição Sumária:**

Tomam parte como profissionais em competições e provas esportivas. Participam, individualmente ou coletivamente, de competições esportivas, em caráter profissional.

- **Formação e Experiência:**

A escolaridade formal não é pré-condição para o exercício das ocupações desta família. A formação prática dos atletas profissionais pode se dar tanto por meio de treinos e exercícios realizados individual e/ou coletivamente, em geral, com a supervisão de treinadores ou técnicos, como por meio de participação em provas, competições, jogos e certames. A(s) ocupação(ões) elencada(s) nesta família ocupacional demanda formação profissional para efeitos do cálculo do número de aprendizes a serem contratados pelos estabelecimentos, nos termos do artigo 429 da Consolidação das Leis do Trabalho - CLT, exceto os casos previstos no art. 10 do Decreto 5.598/2005.

- **Condições Gerais de Exercício:**

Os profissionais trabalham em clubes, agremiações esportivas, academias, órgãos da administração pública afetos aos esportes, no ensino, etc. Não há regras comuns para todas as modalidades de esporte. Para obterem a profissionalização seguem, regras específicas das agremiações esportivas a que se vinculam, construindo, portanto, trajetórias diferenciadas, baseadas em diferentes combinações entre tempo de exercício do esporte, participação em jogos e eventos, premiações, etc. A maioria trabalha como autônomo, em horários irregulares. Em algumas atividades, alguns profissionais podem estar submetidos a condições especiais de trabalho, como pressão psicológica, ruído intenso e altas temperaturas, bem como permanecer por longos períodos em posições desconfortáveis.

- **CBO 413110 - Auxiliar de contabilidade**
- **Descrição Sumária:**

Organizam documentos e efetuam sua classificação contábil; geram lançamentos contábeis, auxiliam na apuração dos impostos, conciliam contas e preenchimento de guias de recolhimento e de solicitações, junto a órgãos do governo. Emitem notas de venda e de transferência entre outras; realizam o arquivo de documentos.

- **Formação e Experiência:**

Embora não exista exigência legal, requer-se escolaridade de nível médio, preferencialmente com curso técnico ou superior incompleto. Não há exigência de experiência anterior. A(s) ocupação(ões) elencada(s) nesta família ocupacional demanda formação profissional para efeitos do cálculo do número de aprendizes a serem contratados pelos estabelecimentos, nos termos do artigo 429 da Consolidação das Leis do Trabalho - CLT, exceto os casos previstos no art. 10 do Decreto 5.598/2005.

- **Condições Gerais de Exercício:**

Exercem suas funções em atividades empresariais como empregados formais. Trabalham com supervisão permanente em ambientes fechados e em horário diurno. Podem trabalhar sob pressão, levando à situação de estresse.

- ## CBO 4151 - Auxiliares de serviços de documentação, informação e pesquisa

Descrição Sumária

Organizam documentos e informações. orientam usuários e os auxiliam na recuperação de dados e informações. disponibilizam fonte de dados para usuários. providenciam aquisição de material e incorporam material ao acervo. arquivam documentos, classificando-os segundo critérios apropriados para armazená-los e conservá-los. prestam serviço de comutação, alimentam base de dados e elaboram estatísticas. executam tarefas relacionadas com a elaboração e manutenção de arquivos, podendo, ainda, operar equipamentos reprográficos, recuperar e preservar as informações por meio digital, magnético ou papel.

Formação e Experiência

O exercício dessas ocupações requer escolaridade de nível médio e curso básico profissionalizante de até duzentas horas. o pleno desempenho das atividades ocorre após um a dois anos de experiência. a(s) ocupação(ões) elencada(s) nesta família ocupacional demanda formação profissional para efeitos do cálculo do número de aprendizes a serem contratados pelos estabelecimentos, nos termos do artigo 429da consolidação das leis do trabalho - clt, exceto os casos previstos no art. 10 do decreto 5.598/2005.

Condições Gerais de Exercício

Trabalham nas mais variadas atividades econômicas onde haja documentos, fitas, vídeos e outros objetos de acervo documental. Predominantemente atuam em bibliotecas e centros de documentação nas áreas de ensino e pesquisa, saúde, serviços sociais, redes de rádio e televisão, bancos, empresas de processamento de dados, em instituições públicas, privadas e ong. organizam-se em equipes e podem atuar em mutirão, sem horário fixo. em algumas atividades, podem atuar à distância (codificador de dados). Trabalham em ambiente fechado, em diferentes horários - diurno, **noturno, em rodízio de turno e em horários irregulares**. podem permanecer por longos períodos em posições desconfortáveis. Em algumas atividades estão sujeitos à poeira e a materiais tóxicos.

- ## CBO 221105 - Biólogo
- ## Descrição Sumária:

Estudam seres vivos, desenvolvem pesquisas na área de biologia, biologia molecular, biotecnologia, biologia ambiental e epidemiologia e inventariam biodiversidade. Organizam coleções biológicas, manejam recursos naturais, desenvolvem atividades de educação ambiental. Realizam diagnósticos biológicos, moleculares e ambientais, além de realizar análises clínicas, citológicas, citogênicas e patológicas. Podem prestar consultorias e assessorias.

- ## Formação e Experiência:

As ocupações da família exigem no mínimo o nível superior completo na área.

- ## Condições Gerais de Exercício:

Trabalham em hospitais, laboratórios, jardins zoológicos e botânicos, parques e reservas naturais, estações bioecológicas e áreas de proteção ambiental, herbários, biotérios, criadouros, estações de cultivo. São encontrados em órgãos governamentais e não-governamentais, empresas públicas e privadas, universidades, como empregados ou prestadores de serviços, consultores, peritos. Quando trabalham em institutos de pesquisa e universidades são geralmente classificados como pesquisadores e professores. O trabalho é exercido em escritórios e laboratórios e também a céu aberto, tanto em período diurno como em rodízio de turnos. Em algumas atividades podem estar **expostos aos efeitos de materiais tóxicos e da radiação**.

- # CBO 4211
- # Caixas e bilheteiros (exceto caixa de banco)
- ## Descrição Sumária:
- Recebem valores de vendas de produtos e serviços; controlam numerários e valores; aten dem o público em agência postal na recepção e entregam objetos postais; recebem contas e tributos e processam remessa e pagamento de numerários por meio postal; vendem bilhetes e ingressos em locais de diversão; processam a arrecadação de prestação de ser viço nas estradas de rodagem; vendem bilhetes no transporte urbano e interurbano; fa zem reserva e emissão de passagens aéreas e terrestres; prestam informações ao público,tais como itinerários, horários, preços, locais, duração de espetáculos, viagens, promoçõese eventos, etc. preenchem formulários e relatórios administrativos.
- ## Formação e Experiência:
- As ocupações dessa família requerem formação inicial equivalente ao ensino funda mental completo para o operador de caixa, ensino médio incompleto para o bilheteiro no serviço de diversão e ensino médio completo para os demais. É na prática, exercitando o trabalho, que o trabalhador completará sua formação. em algumas ocupações é difícil encontrar um profissional com mais de cinco anos de experiência, como, por exemplo, os bilheteiros no serviço de diversão, onde a mão-de-obra empregada é pre dominantemente de jovens em seu primeiro emprego, o que implica em altas taxas de rotatividade. a(s) ocupação(ões) elencada(s) nesta família ocupacional demanda formação profissional para efeitos do cálculo do número de aprendizes a serem contratados pelos estabelecimentos, nos termos do artigo 429 da consolidação das leis dotrabalho - clt, exceto os casos previstos no art. 10 do decreto 5.598/2005
- ## Condições Gerais de Exercício:

Atuam em diversas áreas, tais como correio e telecomunicações, comércio varejista, transportes terrestres e aéreo e atividades recreativas, culturais e desportivas. são em pregados com carteira assinada, trabalham de forma individual com supervisão permanente, ou ocasional como é o caso do emissor de passagem, e em ambientes fechados seus horários são diurno para o atendente comercial e com revezamento de turnos para o restante. Podem trabalhar em locais subterrâneos, como é o caso do bilheteiro.

- # CBO 3721- Captadores de imagens em movimento

- ## Descrição Sumária

Captam imagens pelas câmeras de cinema e vídeo para a realização de produções cinematográficas, televisivas e multimídia, com teor artístico, jornalístico, documental e publicitário. captam imagens

em movimento; interpretam visualmente o roteiro; executam conceito fotográfico e organizam produção de imagens, dialogando constantemente com a equipe de trabalho.

Formação e Experiência

Para o exercício dessas ocupações requer-se, no mínimo, o ensino médio e curso profissionalizante de até duzentas horas/aula (iluminador e operador de câmera de tve vídeo) e curso técnico de nível médio (diretor de fotografia). o desempenho pleno das atividades ocorre após três ou quatro anos de experiência. a(s) ocupação(ões)elencada(s) nesta família ocupacional demanda formação profissional para efeitos do cálculo do número de aprendizes a serem contratados pelos estabelecimentos, nos termos do artigo 429 da consolidação das leis do trabalho - clt, exceto os casos previstos no art. 10 do decreto 5.598/2005.

- ## Condições Gerais de Exercício

Trabalham em atividades culturais, desportivas e de entretenimento, em empresas privadas ou públicas, fundações e órgãos da administração pública, como assalariados (iluminador e operador de câmera) ou por conta própria (diretor de fotografia). o trabalho é desenvolvido em equipe, sob supervisão permanente, nos mais diversos ambientes, em horários irregulares. no exercício de algumas atividades, podem permanecerem posições desconfortáveis por períodos prolongados, bem como estar expostos aos efeitos de ruído intenso, altas temperaturas, grandes alturas e sujeitos a pressões por cumprimento de prazos.

- # CBO 223208 - Cirurgião dentista - clínico geral
- ## Descrição Sumária:

Os cirurgiões dentistas atendem e orientam pacientes e executam procedimentos odontológicos, aplicam medidas de promoção e prevenção de saúde, ações de saúde coletiva, estabelecendo diagnóstico e prognóstico, interagindo com profissionais de outras áreas. Podem desenvolver pesquisas na área odontológica. Desenvolvem atividades profissionais com crianças, adultos e idosos, com ou sem necessidades especiais, em diferentes níveis de complexidade. Podem atuar em consultórios particulares, instituições públicas ou privadas, ongs. Exercem atividade de ensino e pesquisa.

- ## Formação e Experiência:

As ocupações requerem, para seu exercício, formação em odontologia e registro no CRO e atualização constante.

- ## Condições Gerais de Exercício:

Atuam nas áreas de odontologia legal e saúde coletiva, dentística, prótese e prótese maxilofacial, odontopediatria e ortodontia, radiologia, patologia, estomatologia, periodontia, traumatologia bucomaxilofacial e implantologia. Trabalham por conta própria ou como assalariados em clínicas particulares, cooperativas e empresas de atendimento odontológico e na administração pública. Exercem suas atividades individualmente e em equipe. Podem permanecer em posições

desconfortáveis por longos períodos, **estar expostos a radiações, materiais tóxicos,** ruído intenso contaminações e aos riscos de lesões por esforços repetitivos e de distúrbios osteomusculares relacionados ao trabalho (ler-dort).

- **CBO 252205 - Auditor (contadores e afins)**
- **Descrição Sumária:**

Legalizam empresas, elaborando contrato social/estatuto e notificando encerramento junto aos órgãos competentes; administram os tributos da empresa; registram atos e fatos contábeis; controlam o ativo permanente; gerenciam custos; administram o departamento pessoal; preparam obrigações acessórias, tais como declarações acessórias ao fisco, órgãos competentes e contribuintes e administra o registro dos livros nos órgãos apropriados; elaboram demonstrações contábeis; prestam consultoria e informações gerenciais; realizam auditoria interna e externa; atendem solicitações de órgãos fiscalizadores e realizam perícia.

- **Formação e Experiência:**

O exercício dessas ocupações requer curso superior em Ciências contábeis. O desempenho pleno das atividades ocorre após quatro anos (contador) e mais de cinco anos (auditor geral e perito contábil).

- **Condições Gerais de Exercício:**

Trabalham em escritórios de contabilidade e departamentos de contabilidade de empresas agrícolas, industriais, comerciais e dos serviços, incluindo bancos. São empregados com carteira assinada, exceto o perito contábil que trabalha por conta própria e sem supervisão. Costumam se organizar de forma induvidual, trabalhando sob supervisão. Trabalham em ambiente fechado e em horário diurno. Os peritos contábeis podem trabalhar a distância. Eventualmente, trabalham sob pressão, podendo levar à situação de estresse.

- **CBO 318005 - Desenhista técnico**
- **Descrição Sumária:**

Analisam solicitações de desenhos; interpretam documentos de apoio, tais como plantas, projetos, catálogos, croquis e normas. Observam características técnicas de desenhos; esboçam desenhos; definem formatos e escalas, sistemas de representação e prioridades de desenhos, conforme cronogramas. Desenham detalhes de projetos de desenhos. Enviam desenhos para revisão; realizam cópias de segurança e disponibilizam desenhos finais e/ou revisões para áreas afins. São classificados nessa epígrafe os desenhistas técnicos não especializados.

- **Formação e Experiência:**

Para o exercício dessa ocupação requer-se escolaridade de nível médio mais curso profissionalizante básico de duzentas a quatrocentas horas/aula. O exercício pleno das atividades se dará após um a dois anos de experiência profissional. As atividades dos desenhistas técnicos e projetistas diferem. O desenhista técnico não projeta; ele desempenha atividades junto ao desenhista

projetista. A(s) ocupação(ões) elencada(s) nesta família ocupacional demanda formação profissional para efeitos do cálculo do número de aprendizes a serem contratados pelos estabelecimentos, nos termos do artigo 429 da Consolidação das Leis do Trabalho - CLT, exceto os casos previstos no art. 10 do Decreto 5.598/2005.

- **Condições Gerais de Exercício:**

Trabalham em indústrias de construção, de fabricação de máquinas e equipamentos, de eletricidade, gás e água quente, de captação, purificação e distribuição de água e outras atividades empresariais. São empregados com carteira assinada ou por conta própria, que trabalham individualmente com supervisão ocasional. Atuam em ambientes fechados nos horários diurnos. Estão sujeitos ao trabalho em posições desconfortáveis e, eventualmente, pressões. Há a tendência de a ocupação de desenhista projetista incorporar as atividades dos desenhistas copista e detalhista.

- ## CBO 2622 - Diretores de espetáculos e afins

Descrição Sumária

Os diretores de cinema, teatro, televisão e rádio dirigem, criando, coordenando, super visionando e avaliando aspectos artísticos, técnicos e financeiros referentes a realiza ção de filmes, peças de teatro, espetáculos de dança, ópera e musicais, programas detelevisão e rádio, vídeos, multimídia e peças publicitárias.

Formação e Experiência

O exercício das ocupações da família requer formação inicial equivalente ao superior completo. O exercício pleno das atividades demanda pelo menos cinco anos de experiência, uma vez que a expertise esperada advém da prática repetida.

Condições Gerais de Exercício

Trabalham em atividades culturais e recreativas e em outras atividades empresariais. Há intensa mobilidade entre as funções diretor de cinema, tv, vídeo e teatro, sendo que muitos profissionais ora atuam em um veículo ora em outro e também atuam eventualmente como produtores ou atores, de forma concomitante ou isoladamente De forma geral, predomina o vínculo como empregado, entre diretores de tv e rádio e, como autônomo, para as demais ocupações. suas atividades se desenvolvem em equipes, em horários não regulares e alguns profissionais podem estar sujeitos aos efeitos do trabalho sob ruído intenso, altas temperaturas e grandes alturas.

- ## CBO 1114 - Dirigentes do serviço público

- ### Descrição Sumária

Garantem suporte na gestão de pessoas, na administração de material, patrimônio, informática e serviços para as áreas meios e finalísticas da administração pública federal, estadual, distrital e municipal. definem diretrizes, planejam, coordenam e supervisionam ações, monitorando resultados e fomentando políticas de mudança.

- **Formação e Experiência**

Para o exercício desses cargos, geralmente é exigida escolaridade de nível superior eseu provimento é por indicação superior. é vedado o ingresso nesses cargos ao cidadãoque estiver respondendo a processos criminais ou administrativos. a(s) ocupação(ões) elencada(s) nesta família ocupacional demanda formação profissional para efeitos do cálculo do número de aprendizes a serem contratados pelos estabelecimentos, nos termos do artigo 429 da consolidação das leis do trabalho - clt, exceto os casos previstos no art. 10 do decreto 5.598/2005.

- **Condições Gerais de Exercício**

Os ocupantes dos cargos dessa família ocupacional exercem suas atividades nas mais diversas áreas da administração pública federal, estadual, distrital e municipal, como área financeira e contábil, administrativa, de recursos humanos, jurídica, de tecnologia da informação e outras. realizam suas atividades em equipe, sob supervisão ocasional, em ambiente fechado. estão expostos ao assédio de grupos de pressão.

- **CBO 251205 – Economista**
- **Descrição Sumária:**

Analisam o ambiente econômico; elaboram e executam projetos de pesquisa econômica, de mercado e de viabilidade econômica, entre outros. Participam do planejamento estratégico e de curto prazo e avaliam políticas de impacto coletivo para o governo, ONG e outras organizações. Gerem programação econômico-financeira; atuam nos mercados internos e externos; examinam finanças empresariais. Podem exercer mediação, perícia e arbitragem.

- **Formação e Experiência:**

O exercício dessas ocupações requer curso superior em ciências econômicas ou pós-graduação em economia e registro no Conselho Regional de Economia. O desempenho pleno das atividades ocorre após um ou dois anos de experiência na área.

- **Condições Gerais de Exercício:**

Atuam em empresas das diversas atividades econômicas como intermediação financeira, seguros e previdência privada; administração pública, seguridade social; empresas de consultoria econômica; na agricultura, pecuária, indústria e serviços relacionados com essas atividades; no comércio por atacado e intermediários do comércio. São majoritariamente estatutários ou assalariados com carteira assinada; trabalham em equipe, com supervisão permanente, em ambiente fechado e em horário diurno.

- **CBO 7687- Encadernadores e recuperadores de livros (pequenos lotes ou a unidade) Descrição Sumária**

Encadernam, douram e gravam manualmente e por meio de diversos processos gráficos e artísticos, folhas, documentos e livros. Realizam impressão hot stamp.

Formação e Experiência

O exercício dessas ocupações requer ensino fundamental seguido de curso de qualificação profissional de duzentas a quatrocentas horas/aula. o exercício pleno das atividades demanda um ano de prática. a(s) ocupação(ões) elencada(s) nesta família ocupacional demanda formação profissional para efeitos do cálculo do número de aprendizes a serem contratados pelos estabelecimentos nos termos do artigo 429da consolidação das leis do trabalho - clt, exceto os casos previstos no art. 10 do decreto 5.598/2005.

- ## Condições Gerais de Exercício

- Podem trabalhar como assalariados, registrados em carteira ou como autônomos. Trabalham em empresas de edição, impressão e reprodução de gravações. atuam, também, na restauração e recuperação gráfica e artística para deixar a peça em seu estado original. Trabalham de forma individual e sob supervisão ocasional. em algumas atividades, o trabalhador permanece em posições desconfortáveis durante longos períodos e em contato com **materiais tóxicos**.

-

- # CBO 2140 - Engenheiros ambientais e afins

- ## Descrição Sumária

Elaboram e implantam projetos ambientais; gerenciam a implementação do sistema de gestão ambiental (sga) nas empresas, implementam ações de controle de emissão de poluentes, administram resíduos e procedimentos de remediação. podem prestar consultoria, assistência e assessoria.

- ## Formação e Experiência

Para o exercício profissional requer-se formação em engenharia ambiental ou cursos de tecnologia na área ambiental e registro no respectivo conselho de classe, quando exigido o exercício pleno da atividade ocorre, em média, após um a dois anos de experiência.

- ## Condições Gerais de Exercício

- Atuam na maioria das atividades econômicas, com foco na reciclagem, eletricidade, gás, captação, purificação e distribuição de água, além da silvicultura, exploração florestal. Costumam trabalhar em equipe multidisciplinar, em laboratórios e escritórios e também a céu aberto, ou no campo. Os vínculos de trabalho mais comum são como trabalhador assalariado, ou por conta própria, na condição de prestador de serviços.

- # CBO 2142 - Engenheiros civis e afins

Descrição Sumária

Elaboram projetos de engenharia civil, gerenciam obras, controlam a qualidade de empreendimentos. Coordenam a operação e manutenção do empreendimento. Podem prestar consultoria, assistência e assessoria e elaborar pesquisas tecnológicas.

Formação e Experiência

Para o exercício profissional requer-se formação em nível superior em tecnologia em construção civil ou engenharia civil e registro no CREA, sendo frequente os profissionais porta dores de títulos de especialização e pós-graduação lato sensu. o exercício pleno da atividade ocorre, em média, após cinco anos de experiência, para engenheiros civis e em média até dois anos no caso dos tecnólogos.

Condições Gerais de Exercício

Atuam na maioria das atividades econômicas com concentração na construção civil. Trabalham na área de planejamento e gerenciamento de projetos construtivos para as mais diversas finalidades. Costumam trabalhar em equipe multidisciplinar, em laboratórios e escritórios e também a céu aberto, ou no campo. os vínculos de trabalho mais comuns são como trabalhador assalariado ou por conta própria, na condição de prestador de serviços. Eventualmente, em certas atividades, alguns profissionais trabalham em condições especiais, por exemplo, em ambientes subterrâneos ou confinados, **expostos à poeira**, mau cheiro, ruído intenso e **materiais tóxicos.**

- # CBO 214705 - Engenheiro de minas
- ## Descrição Sumária:

Projetam, planejam, implantam e supervisionam atividades e/ou empreendimentos de prospecção, extração e beneficiamento de minérios, petróleo e gás, tais como perfuração, desmonte, escavação, carregamento, transporte, classificação, lavagem, concentração, secagem e embalagem de embarque. Inspecionam áreas de interesse, avaliando riscos da atividade e gerenciando recursos humanos, financeiros e materiais. Podem prestar consultoria e assistência técnica.

- ## Formação e Experiência:

O exercício profissional requer formação superior em Engenharia de Minas, Tecnologia em Rochas Ornamentais ou Tecnologia em Petróleo e Gás e registro no Crea. É cada vez mais crescente, no mercado de trabalho, a presença de profissionais com pósgraduação e cursos de especialização.

- ## Condições Gerais de Exercício:

Os profissionais trabalham, principalmente, na extração de carvão mineral, minerais metálicos e outros minerais, além da cadeia produtiva de petróleo e gás. Predominantemente, são encontrados em grandes empresas mineradoras, como empregados registrados e empresas relacionadas à cadeia de petróleo e gás. Podem também ser encontrados em universidades e institutos de pesquisa públicos ou privados. Nesses casos, são classificados como professores e pesquisadores. Trabalham costumeiramente em equipe multidisciplinar com supervisão ocasional. Em algumas atividades,

podem estar sujeitos a condições especiais de trabalho, como **exposição a materiais tóxicos**, ruído, altas temperaturas, **poeira** e umidade, bem como ao **trabalhos subterrâneos, em grandes alturas ou confinados**.

CBO 2143 - Engenheiros eletricistas, eletrônicos e afins

- ## Descrição Sumária

Executam serviços elétricos, eletrônicos e de telecomunicações, analisando propostas técnicas, instalando, configurando e inspecionando sistemas e equipamentos, executando testes e ensaios. projetam, planejam e especificam sistemas e equipamentos elétricos, eletrônicos e de telecomunicações e elaboram sua documentação técnica; coordenam empreendimentos e estudam processos elétricos, eletrônicos e de telecomunicações.

- ## Formação e Experiência

O exercício profissional requer formação em uma das áreas de engenharia: elétrica, eletrônica ou telecomunicações ou curso de tecnólogo em uma das áreas, com registro no crea. o exercício pleno das atividades ocorre, em média, com quatro anos de exercício profissional para os engenheiros e de 1 a 2 anos para os tecnólogos, incluindo tempo de estágio. a manutenção do emprego neste domínio requer de seus profissionais atualização constante.

- ## Condições Gerais de Exercício

Trabalham em ramos de atividade econômica variados. na área industrial, encontram-se na fabricação de máquinas, aparelhos e materiais elétricos e eletrônicos e de equipamentos de telecomunicação. É expressiva a presença desses profissionais na atividade de serviços de apoio à indústria, tais como empresas de manutenção, na geração, distribuição e transmissão de energia elétrica, água e gás, e na construção civil. Trabalham em empresas pequenas, médias e grandes nas áreas pública e privada, como empregados ou prestadores de serviços. Suas atividades costumam se desenvolver em equipe multidisciplinar com supervisão ocasional em algumas atividades, podem estar submetidos a condições especiais de trabalho, por exemplo, grandes alturas, altas temperaturas, ruído intenso, exposição a **material tóxico**, alta tensão e **radiação**.

CBO 214405 - Engenheiro mecânico

- ## Descrição Sumária:

Projetam sistemas e conjuntos mecânicos, componentes, ferramentas e materiais, especificando limites de referência para cálculo, calculando e desenhando. Implementam atividades de manutenção, testam sistemas, conjuntos mecânicos, componentes e ferramentas, desenvolvem atividades de fabricação de produtos e elaboram documentação técnica. Podem coordenar e assessorar atividades técnicas.

- **Formação e Experiência:**

O exercício das ocupações requer formação em curso superior de Engenharia Mecânica e afins ou Tecnologia em Fabricação Mecânica (ou outra formação correlacionada), com registro no Crea. A tendência do mercado atualmente é valorizar profissionais com pós-graduação e cursos de especialização. Em média, para o exercício pleno das atividades, demanda-se uma experiência superior a cinco anos para os engenheiros e, no caso dos tecnólogos, de 1 a 2 anos.

- **Condições Gerais de Exercício:**

Trabalham nos setores industrial e de serviços. Na indústria, são empregados principalmente na metalurgia básica, fabricação de máquinas, equipamentos e veículos automotores, mas podem atuar em outros ramos industriais, tais como alimentos, têxtil e confecções, entre outros. Trabalham em equipe multidisciplinar, sob supervisão ocasional. Seu vínculo mais comum é como assalariado de carteira assinada nas esferas pública e privada. Também são encontrados em universidades e institutos de pesquisa e, nesses casos, são classificados como pesquisadores e professores.

- **CBO 411 - Escriturários em geral, agentes, assistentes e auxiliares administrativos**

- **Descrição Sumária**

Executam serviços de apoio nas áreas de recursos humanos, administração, finanças e logística; atendem fornecedores e clientes, fornecendo e recebendo informações sobre produtos e serviços; tratam de documentos variados, cumprindo todo o procedimento necessário referente aos mesmos. atuam na concessão de microcrédito a microempresários, atendendo clientes em campo e nas agências, prospectando clientes nas comunidades.

- Formação e Experiência

Para o acesso às ocupações dessa família ocupacional requer-se o ensino médio completo, curso básico de qualificação de até duzentas horas/aula e de um a dois anos de experiência profissional. a(s) ocupação(ões) elencada(s) nesta família ocupacional demanda formação profissional para efeitos do cálculo do número de aprendizes a serem contratados pelos estabelecimentos, nos termos do artigo 429da consolidação das leis do trabalho - clt, exceto os casos previstos no art. 10 do decreto 5. 598/2005.

- Condições Gerais de Exercício

- Trabalham nos mais variados ramos de atividades públicas ou privadas. São empregados com carteira e se organizam em equipe, tendo supervisão ocasional. o ambiente de trabalho é fechado e o horário é diurno. A categoria de "auxiliares" foi extinta nos orgãos públicos e suas

funções são realizadas pelos assistentes administrativos. Já no caso da área privada, a categoria de "auxiliares e ajudantes" parece estar em processo de extinção e suas funções sendo incorporadas pelos assistentes ou pelos estagiários. Os agentes de microcrédito atuam junto às comunidades em ambientes abertos.

- **CBO 223405 – Farmacêutico**
- **Descrição Sumária:**

Realizam tarefas específicas de desenvolvimento, produção, dispensação, controle, armazenamento, distribuição e transporte de produtos da área farmacêutica tais como medicamentos, alimentos especiais, cosméticos, imunobiológicos, domissanitários e insumos correlatos. Realizam análises clínicas, toxicológicas, fisioquímicas, biológicas, microbiológicas e bromatológicas; participam da elaboração, coordenação e implementação de políticas de medicamentos; exercem fiscalização sobre estabelecimentos, produtos, serviços e exercício profissional; orientam sobre uso de produtos e prestam serviços farmacêuticos. Podem realizar pesquisa sobre os efeitos de medicamentos e outras substâncias sobre órgãos, tecidos e funções vitais dos seres humanos e dos animais.

- **Formação e Experiência:**

O acesso a essas ocupações requer curso superior em Farmácia bioquímica. Atualmente a formação é única, substituindo as duas formações anteriores: a de farmácia e a de bioquímica.

- **Condições Gerais de Exercício:**

Trabalham em órgãos públicos, principalmente os de vigilância sanitária, em farmácias e redes de distribuição de remédios, nas indústrias de produtos farmacêuticos e correlatos, nas indústrias de produtos alimentares, de cosméticos, dentre outras. Trabalham como assalariados, com carteira assinada, por conta própria ou como empregadora.

- **CBO 2614 - Filólogos, tradutores ,intérpretes e afins**
- Descrição Sumária

Traduzem, na forma escrita, textos de qualquer natureza, de um idioma para outro,considerando as variáveis culturais, bem como os aspectos terminológicos e estilísticos,tendo em vista um público-alvo específico. interpretam oralmente e/ou na língua desinais, de forma simultânea ou consecutiva, de um idioma para outro, discursos, de bates, textos, formas de comunicação eletrônica, respeitando o respectivo contexto eas características culturais das partes. tratam das características e do desenvolvimentode uma cultura, representados por sua linguagem; fazem a crítica dos textos. prestamassessoria a clientes.

- Formação e Experiência

As ocupações da família requerem formações diferenciadas: o superior completo para filó logos e linguistas e o ensino médio ou o diploma de técnico para tradutores e intérpretes. O desenvolvimento pleno das atividades demanda experiência superior a cinco anos.

- ## Condições Gerais de Exercício

Trabalham em serviços especializados de eventos, congressos e seminários, de atividades empresariais variadas, da administração pública, em empresas, universidades, fundações e outras instituições, de caráter público ou privado. a maioria dos tradutores e intérpretes trabalha como autônomos, seja de forma individual ou em grupos, por projetos, podendo desenvolver suas atividades também à distância. Os filólogos trabalham de forma individual, predominantemente como empregado. os profissionais podem trabalhar em horários irregulares e, em algumas atividades, estar sujeitos a permanências prolongadas em posições desconfortáveis, a ruídos intensos, bem como a trabalhos sob pressão de prazos. esta família não compreende2346 - professores nas áreas de língua e literatura do ensino superior.

- # CBO 223605 - Fisioterapeuta geral
- ## Descrição Sumária:

Aplicam técnicas fisioterapêuticas para prevenção, readaptação e recuperação de pacientes e clientes. Atendem e avaliam as condições funcionais de pacientes e clientes utilizando protocolos e procedimentos específicos da fisioterapia e suas especialidades. Atuam na área de educação em saúde por meio de palestras, distribuição de materiais educativos e orientações para melhor qualidade de vida. Desenvolvem e implementam programas de prevenção em saúde geral e do trabalho. Gerenciam serviços de saúde orientando e supervisionando recursos humanos. Exercem atividades técnico-científicas através da realização de pesquisas, trabalhos específicos, organização e participação em eventos científicos.

- ## Formação e Experiência:

Para o exercício dessas ocupações é exigido curso superior na área de fisioterapia, com registro no conselho profissional pertinente.

- ## Condições Gerais de Exercício:

Trabalham nas áreas de saúde, de educação e de serviços sociais, em caráter liberal e/ ou com vínculo empregatício ou ainda na prestação de serviços terceirizados, de forma individual ou em equipes multiprofissionais. Atuam em consultórios, hospitais, ambulatórios clínicas, escolas, domicílios, clubes, comunidades, escolas e indústrias, em ambientes fechados ou abertos, em horários diurnos e **noturnos**. Podem permanecer em posições desconfortáveis por longos períodos ou ser expostos a elementos **biopatogênicos**.

- # CBO 213105 – Físico
- ## Descrição Sumária:

Aplicam princípios, conceitos e métodos da física em atividades específicas, aplicam técnicas de radiação ionizante e não ionizante em ciências da vida, radiação na agricultura e conservação de alimentos e podem operar reatores nucleares e equipamentos emissores de radiação. Desenvolvem fontes alternativas de energia, projetam sistemas eletrônicos, ópticos, de telecomunicações e outros sistemas físicos. Realizam medidas de grandezas físicas, desenvolvem programas e rotinas computacionais e elaboram documentação técnica e científica.

Formação e Experiência:

As ocupações exigem para seu exercício, pelo menos, o curso superior completo, sendo frequente a presença de profissionais com titulações de pós-graduação e cursos de especialização. O exercício pleno das atividades das ocupações geralmente ocorre após quatro anos de experiência na área.

Condições Gerais de Exercício:

Os profissionais podem trabalhar em várias atividades econômicas como, por exemplo, saúde, fabricação de coque, refino de petróleo, produção de combustíveis, inclusive nucleares, fabricação de material eletrônico e aparelhos de comunicação. É mais comum encontrar esses profissionais em institutos de pesquisa e universidades públicas. Nestes casos, são classificados como pesquisadores ou professores. Costumam trabalhar em equipe multidisciplinar com supervisão ocasional. Pode ocorrer que, no exercício de algumas atividades, alguns profissionais estejam expostos aos **efeitos da radiação e de materiais tóxicos.**

CBO 513405 – Garçom

Descrição Sumária:

Atendem os clientes, recepcionando-os e servindo refeições e bebidas em restaurantes, bares, clubes, cantinas, hotéis, eventos e hospitais; montam e desmontam praças, carrinhos, mesas, balcões e bares; organizam, conferem e controlam materiais de trabalho, bebidas e alimentos, listas de espera, a limpeza e higiene e a segurança do local de trabalho; preparam alimentos e bebidas, realizando também serviços de vinhos.

Formação e Experiência:

Para o acesso ao trabalho nessa família ocupacional é exigido, no mínimo, o ensino fundamental incompleto e até um ano de experiência. A formação profissional ocorre com a prática no local de trabalho ou em cursos profissionalizantes. O trabalhador tem a possibilidade de ascender na carreira, iniciando como ajudante ou auxiliar, podendo atingir a ocupação de maître (família ocupacional 5101). O sommelier, para o qual é requerido o ensino médio, necessita de especialização e experiência de um a dois anos. O mercado de trabalho tende a aumentar a exigência do nível de qualificação e profissionalização desses trabalhadores. A(s) ocupação(ões) elencada(s) nesta família ocupacional demanda formação profissional para efeitos do cálculo do número de aprendizes a serem contratados pelos estabelecimentos, nos termos do artigo 429 da Consolidação das Leis do Trabalho - CLT, exceto os casos previstos no art. 10 do Decreto 5.598/2005.

Condições Gerais de Exercício:

Os trabalhadores dessas ocupações, normalmente, têm vínculo de trabalho assalariado, com carteira assinada ou, eventualmente, trabalham como autônomos com comissão. Atuam, predominantemente, no ramo de atividades ligadas a hotelaria, alimentação, saúde, serviços sociais e atividades recreativas, culturais e desportivas. Trabalham em equipe, em ambientes fechados, com revezamento de turnos e supervisão permanente. Algumas das atividades podem ser exercidas sob pressão, em posições desconfortáveis durante longos períodos e com exposição a ruído intenso, podendo levar à situação de estresse. Eventualmente, em hospitais, podem ser expostos à radiação.

- **CBO 513420 - Barman**
- **Descrição Sumária:**

Atendem os clientes, recepcionando-os e servindo refeições e bebidas em restaurantes, bares, clubes, cantinas, hotéis, eventos e hospitais; montam e desmontam praças, carrinhos, mesas, balcões e bares; organizam, conferem e controlam materiais de trabalho, bebidas e alimentos, listas de espera, a limpeza e higiene e a segurança do local de trabalho; preparam alimentos e bebidas, realizando também serviços de vinhos.

- **Formação e Experiência:**

Para o acesso ao trabalho nessa família ocupacional é exigido, no mínimo, o ensino fundamental incompleto e até um ano de experiência. A formação profissional ocorre com a prática no local de trabalho ou em cursos profissionalizantes. O trabalhador tem a possibilidade de ascender na carreira, iniciando como ajudante ou auxiliar, podendo atingir a ocupação de maître (família ocupacional 5101). O sommelier, para o qual é requerido o ensino médio, necessita de especialização e experiência de um a dois anos. O mercado de trabalho tende a aumentar a exigência do nível de qualificação e profissionalização desses trabalhadores. A(s) ocupação(ões) elencada(s) nesta família ocupacional demanda formação profissional para efeitos do cálculo do número de aprendizes a serem contratados pelos estabelecimentos, nos termos do artigo 429 da Consolidação das Leis do Trabalho - CLT, exceto os casos previstos no art. 10 do Decreto 5.598/2005.

- **Condições Gerais de Exercício:**

Os trabalhadores dessas ocupações, normalmente, têm vínculo de trabalho assalariado, com carteira assinada ou, eventualmente, trabalham como autônomos com comissão. Atuam, predominantemente, no ramo de atividades ligadas a hotelaria, alimentação, saúde, serviços sociais e atividades recreativas, culturais e desportivas. Trabalham em equipe, em ambientes fechados, com revezamento de turnos e supervisão permanente. Algumas das atividades podem ser exercidas sob pressão, em posições desconfortáveis durante longos períodos e com exposição a ruído intenso, podendo levar à situação de estresse. **Eventualmente, em hospitais, podem ser expostos à radiação.**

- **CBO 513425 - Copeiro**
- **Descrição Sumária:**

Atendem os clientes, recepcionando-os e servindo refeições e bebidas em restaurantes, bares, clubes, cantinas, hotéis, eventos e hospitais; montam e desmontam praças, carrinhos, mesas, balcões e bares; organizam, conferem e controlam materiais de trabalho, bebidas e alimentos, listas de espera, a limpeza e higiene e a segurança do local de trabalho; preparam alimentos e bebidas, realizando também serviços de vinhos.

- **Formação e Experiência:**

Para o acesso ao trabalho nessa família ocupacional é exigido, no mínimo, o ensino fundamental incompleto e até um ano de experiência. A formação profissional ocorre com a prática no local de trabalho ou em cursos profissionalizantes. O trabalhador tem a possibilidade de ascender na

carreira, iniciando como ajudante ou auxiliar, podendo atingir a ocupação de maître (família ocupacional 5101). O sommelier, para o qual é requerido o ensino médio, necessita de especialização e experiência de um a dois anos. O mercado de trabalho tende a aumentar a exigência do nível de qualificação e profissionalização desses trabalhadores. A(s) ocupação(ões) elencada(s) nesta família ocupacional demanda formação profissional para efeitos do cálculo do número de aprendizes a serem contratados pelos estabelecimentos, nos termos do artigo 429 da Consolidação das Leis do Trabalho - CLT, exceto os casos previstos no art. 10 do Decreto 5.598/2005.

- ## Condições Gerais de Exercício:

Os trabalhadores dessas ocupações, normalmente, têm vínculo de trabalho assalariado, com carteira assinada ou, eventualmente, trabalham como autônomos com comissão. Atuam, predominantemente, no ramo de atividades ligadas a hotelaria, alimentação, saúde, serviços sociais e atividades recreativas, culturais e desportivas. Trabalham em equipe, em ambientes fechados, com revezamento de turnos e supervisão permanente. Algumas das atividades podem ser exercidas sob pressão, em posições desconfortáveis durante longos períodos e com exposição a ruído intenso, podendo levar à situação de estresse. **Eventualmente, em hospitais, podem ser expostos à radiação.**

- # CBO 513410 - Sommelier
- ## Descrição Sumária:

Atendem os clientes, recepcionando-os e servindo refeições e bebidas em restaurantes, bares, clubes, cantinas, hotéis, eventos e hospitais; montam e desmontam praças, carrinhos, mesas, balcões e bares; organizam, conferem e controlam materiais de trabalho, bebidas e alimentos, listas de espera, a limpeza e higiene e a segurança do local de trabalho; preparam alimentos e bebidas, realizando também serviços de vinhos.

- ## Formação e Experiência:

Para o acesso ao trabalho nessa família ocupacional é exigido, no mínimo, o ensino fundamental incompleto e até um ano de experiência. A formação profissional ocorre com a prática no local de trabalho ou em cursos profissionalizantes. O trabalhador tem a possibilidade de ascender na carreira, iniciando como ajudante ou auxiliar, podendo atingir a ocupação de maître (família ocupacional 5101). O sommelier, para o qual é requerido o ensino médio, necessita de especialização e experiência de um a dois anos. O mercado de trabalho tende a aumentar a exigência do nível de qualificação e profissionalização desses trabalhadores. A(s) ocupação(ões) elencada(s) nesta família ocupacional demanda formação profissional para efeitos do cálculo do número de aprendizes a serem contratados pelos estabelecimentos, nos termos do artigo 429 da Consolidação das Leis do Trabalho - CLT, exceto os casos previstos no art. 10 do Decreto 5.598/2005.

- ## Condições Gerais de Exercício:

Os trabalhadores dessas ocupações, normalmente, têm vínculo de trabalho assalariado, com carteira assinada ou, eventualmente, trabalham como autônomos com comissão. Atuam, predominantemente, no ramo de atividades ligadas a hotelaria, alimentação, saúde, serviços sociais

e atividades recreativas, culturais e desportivas. Trabalham em equipe, em ambientes fechados, com revezamento de turnos e supervisão permanente. Algumas das atividades podem ser exercidas sob pressão, em posições desconfortáveis durante longos períodos e com exposição a ruído intenso, podendo levar à situação de estresse. **Eventualmente, em hospitais, podem ser expostos à radiação.**

- ## CBO 1412-05 - Gerente de produção e operações

- ### Descrição Sumária

Exercem a gerência de produção nas indústrias de transformação e extração mineral; definem e implementam plano operacional, analisando a demanda de produtos, a capacidade produtiva e recursos auxiliares, elaborando plano de racionalização e redução de custos, plano de investimentos, orçamento de despesas e necessidades de matérias primas; planejam a produção, programando mão-de-obra e paradas ou intervenções em máquinas, equipamentos e instrumentos industriais; gerenciam equipes de trabalho, ad ministrando salários, admissões, demissões, promoções e promovendo o desenvolvimento das equipes por meio de cursos e treinamentos; asseguram e promovem o cumprimento das ações de proteção ao meio ambiente e também pelas normas de higiene e segurança no trabalho, por meio de orientações às suas equipes; desenvolvem e implantam métodos e técnicas que visam melhorar e otimizar o processo de produção; gerenciam áreas de manutenção, engenharia de processos e logística.

- ### Formação e Experiência

Para ingressar nessa família ocupacional, o trabalhador necessita do curso superior completo. Normalmente são formados em engenharia plena ou de curta duração e afins. É recomendável mais de cinco anos de experiência profissional. O gerente de produção e operações está sempre em busca de novas tecnologias e em constante reciclagem. a(s)ocupação(ões) elencada(s) nesta família ocupacional demanda formação profissional para efeitos do cálculo do número de aprendizes a serem contratados pelos estabelecimentos, nos termos do artigo 429 da consolidação das leis do trabalho - clt, exceto os casos previstos no art. 10 do decreto 5.598/2005.

- ### Condições Gerais de Exercício

- Esses profissionais atuam em áreas ligadas à extração de minerais, comércio por atacado e intermediários do comércio, comércio varejista, exceto comércio de veículo, fabricação de produtos alimentares e bebidas e de produtos químicos. São empregados com carteira assinada e trabalham em equipes, com supervisão ocasional, em ambientes fechados e em horário diurno. Eventualmente, trabalham sob pressão, levando à situação de estresse constante.

- ## CBO 142105 - Gerente administrativo
- ## Qual o CBO para Gerente administrativo ?

1 - MEMBROS SUPERIORES DO PODER PÚBLICO, DIRIGENTES DE ORGANIZAÇÕES DE INTERESSE PÚBLICO E DE EMPRESAS, GERENTES
14 - GERENTES
142 - GERENTES DE ÁREAS DE APOIO
1421 - GERENTES ADMINISTRATIVOS, FINANCEIROS, DE RISCOS E AFINS
142105 - Gerente administrativo

- **Descrição Sumária:**

Exercem a gerência dos serviços administrativos, das operações financeiras e dos riscos em empresas industriais, comerciais, agrícolas, públicas, de educação e de serviços, incluindo as do setor bancário. Gerenciam recursos humanos, administram recursos materiais e serviços terceirizados de sua área de competência. Planejam, dirigem e controlam os recursos e as atividades de uma organização, com o objetivo de minimizar o impacto financeiro da materialização dos riscos.

- **Formação e Experiência:**

Para o exercício das ocupações de gerentes administrativos e financeiros, a escolaridade varia em função do porte da instituição empregadora: curso superior incompleto e cursos profissionalizantes de até quatrocentas horas ou graduação tecnológica, bacharelado e de pós-graduação. Os requisitos para os gerentes de riscos são mais elevados – curso superior mais pós-graduação na área e conhecimento do negócio em que atua. Neste caso, o pleno desempenho das atividades ocorre em torno de cinco anos gerenciando riscos em uma área específica. A(s) ocupação(ões) elencada(s) nesta família ocupacional demanda formação profissional para efeitos do cálculo do número de aprendizes a serem contratados pelos estabelecimentos, nos termos do artigo 429 da Consolidação das Leis do Trabalho - CLT, exceto os casos previstos no art. 10 do Decreto 5. 598/2005.

- **Condições Gerais de Exercício:**

Atuam em empresas industriais, comerciais, agrícolas, públicas, de educação e de serviços, incluindo as de intermediação financeira, em atividades gerenciais de apoio à atividade fim, predominantemente como assalariados, com carteira assinada. Trabalham em equipe, sob supervisão ocasional, atuando em ambientes fechados e em horário diurno.

- **CBO 334110 - Inspetor de alunos de escola pública Descrição Sumária:**

Cuidam da segurança do aluno nas dependências e proximidades da escola; inspecionam o comportamento dos alunos no ambiente escolar. Orientam alunos sobre regras e procedimentos, regimento escolar, cumprimento de horários; ouvem reclamações e analisam fatos. Prestam apoio às atividades acadêmicas; controlam as atividades livres dos alunos, orientando entrada e saída de alunos, fiscalizando espaços de recreação, definindo limites nas atividades livres. Organizam ambiente escolar e providenciam manutenção predial.

- **Formação e Experiência:**

O exercício dessas ocupações requer ensino fundamental (inspetor de alunos de escola pública) e ensino médio (inspetor de alunos de escola privada). O inspetor de alunos de escola pública é recrutado por meio de concurso público.

- ## Condições Gerais de Exercício:

Trabalham em estabelecimento de ensino público, privado ou em escolas livres. São estatutários ou celetistas. Atuam em equipe, em locais abertos ou fechados, em período diurno ou noturno, sob supervisão ocasional de diretores ou secretários de escola. Podem permanecer em pé por períodos longos, em locais ruidosos. Os profissionais que atuam em escola pública assumem, também, funções pertinentes a outros profissionais administrativos e pedagógicos como, por exemplo, ouvir reclamações de professores sobre ameaças de alunos em áreas de violência, orientar alunos e fazer pequenos reparos nas escolas.

- # CBO 3331 - Instrutores e professores de cursos livres

- ## Descrição Sumária

Os profissionais dessa família ocupacional devem ser capazes de criar e planejar cursos livres, elaborar programas para empresas e clientes, definir materiais didáticos, ministrar aulas, avaliar alunos e sugerir mudanças estruturais em cursos.

- ## Formação e Experiência

O exercício dessas ocupações é livre. Requer-se escolaridade e qualificação profissional variadas, dependendo da área de atuação. os cursos livres não estão sujeitos à regulamentação do mec.

- ## Condições Gerais de Exercício

Exercem suas funções em instituições de ensino, basicamente em escolas que oferecem cursos livres. atuam de forma individual e também em equipe; trabalham com supervisão ocasional e, dependendo da característica do curso, podem atuar em ambiente fechado, a céu aberto e em veículos. na grande maioria, trabalham na condição de profissionais autônomos, atuando nos períodos diurno e noturno.

- # CBO 3544 - Leiloeiros e avaliadores

- ## Descrição Sumária

Captam, avaliam, oficializam, divulgam, administram e organizam leilões de bens móveis e imóveis, novos ou usados e semoventes. Emitem pareceres técnicos e comerciais sobre os bens a serem leiloados ou comercializados.

- ## Formação e Experiência

O exercício profissional é aberto a brasileiros, maiores de vinte e cinco anos que cum pram exigências de legislação específica. os trabalhadores que exercem essas ocupações possuem escolaridade variada. em geral, complementam sua formação com cursos cuja duração variam entre duzentas e quatrocentas horas. a(s) ocupação(ões)elencada(s) nesta família ocupacional demanda formação profissional para efeitos do cálculo do número de aprendizes a serem contratados pelos estabelecimentos, nos termos do artigo 429 da consolidação das leis do trabalho - clt, exceto os casos previstos no art. 10 do decreto 5.598/2005.

- ## Condições Gerais de Exercício

Prestam serviços a pessoas, instituições públicas e privadas. Atuam em atividades imobiliárias, de extração de minerais, empresariais e artísticas, podendo, os leilões, serem feitos para indústrias, comércio ou serviços. Trabalham em empresas ou por conta própria, em equipe e, ocasionalmente, com supervisão. o local de trabalho varia de ambientes fechados a céu aberto ou em veículos e os horários costumam ser irregulares. podem trabalhar em posições desconfortáveis por longos períodos e sob pressão. Em algumas situações, o avaliador de bens móveis **é exposto a materiais tóxicos e à radiação** e o leiloeiro à agressão de pessoas durante leilão.

- # CBO 2617- Locutores, comentaristas e repórteres de rádio e televisão

- ## Descrição Sumária

Apresentam programas de rádio e televisão, ancorando programas, nos quais interpretam o conteúdo da apresentação, noticiam fatos, leem textos no ar, redigem a notícia, narram eventos esportivos e culturais, tecem comentários sobre os mesmos e fazem a locução de anúncios publicitários; entrevistam pessoas; anunciam programação; preparam conteúdo para apresentação, pautando o texto, checando as informações, adaptando-se aos padrões da emissora e do público alvo; atuam em rádio, televisão e eventos, bem como em mídias alternativas como cinema e internet.

- ## Formação e Experiência

O exercício dessas ocupações requer curso superior, sendo atualmente, a locução de rádio e tv, principalmente de programas jornalísticos, uma atividade desempenhada por profissionais com formação em jornalismo. no caso específico de locutores, apenas os profissionais com formação em jornalismo podem redigir e comentar a notícia, em função da legislação e regulamentação da profissão. portanto, os locutores sem formação em jornalismo foram sendo gradualmente substituídos nos noticiários de rádio e tv. No caso dos comentaristas esportivos, não é obrigatória a formação em jornalismo, sendo frequente ex-jogadores e técnicos comentarem os eventos. quando necessário também se habilitam como radialistas, fazendo cursos de locução de até duzentas horas/aula ou de radialista em nível técnico e superior. varia de um a dois anos a experiência

profissional para os locutores e narradores de rádio e televisão, de quatro a cinco anos para o locutor publicitário de rádio e tv e mais de cinco para o comentarista e âncora de rádio e tv para o pleno desempenho das atividades.

- ## Condições Gerais de Exercício

- Atuam na área de atividades recreativas, culturais e desportivas. Executam suas funções como empregados com carteira assinada e, no caso do locutor publicitário de rádio e TV, como autônomo. São profissionais multifuncionais, que atualmente participam integralmente do processo, desde a produção até a apresentação da notícia, que trabalham em equipe, com supervisão ocasional, em ambiente fechado ou a céu aberto, no caso do comentarista de rádio e TV. A maioria dos profissionais trabalha em várias emissoras de rádio e TV, apresentando diversos programas, cumprindo horários flexíveis (manhã, noite, tarde, diariamente, etc.), podendo trabalhar também a distância. Eventualmente, trabalham sob pressão, levando à situação de estresse constante e expostos a ruído intenso, **a radiação** (locutor de rádio e TV) e a altas temperaturas (narrador em programas de rádio e TV e repórter de rádio e TV).

- # CBO 9113 - Mecânicos de manutenção de máquinas industriais

- ## Descrição Sumária

Realizam manutenção em componentes, equipamentos e máquinas industriais; planejam atividades de manutenção; avaliam condições de funcionamento e desempenho de componentes de máquinas e equipamentos; lubrificam máquinas, componentes e ferramentas. Documentam informações técnicas; realizam ações de qualidade e preservação ambiental e trabalham de acordo com normas de segurança.

- ## Formação e Experiência

O exercício dessas ocupações requer escolaridade de ensino médio, acrescida de cursos básicos de qualificação, com mais de quatrocentas horas/aula. o exercício pleno das atividades ocorre após um período de quatro a cinco anos de experiência profissional.a(s) ocupação(ões) elencada(s) nesta família ocupacional demanda formação profissional para efeitos do cálculo do número de aprendizes a serem contratados pelos

- ## Condições Gerais de Exercício

- Podem exercer suas funções em empresas do ramo de fabricação de máquinas e equipamentos, de fabricação de produtos têxteis, de fabricação de máquinas, aparelhos e materiais elétricos, entre outras. são contratados na condição de empregados com registro em carteira. Trabalham de forma individual, com supervisão ocasional, em ambientes fechados, nos períodos diurno e noturno, podendo atender ocorrências fora do horário de expediente. Podem permanecer

em posições desconfortáveis e estar expostos à ação de **materiais tóxicos,** ruído intenso e altas temperaturas. estão sujeitos a trabalhos sob pressão, levando-os à situação de estresse.

-

- ## CBO 225125 - Médicos clínicos

- ### Descrição Sumária

- Realizam consultas e atendimentos médicos, tratam pacientes e clientes, implementam ações de prevenção de doenças e promoção da saúde tanto individuais quanto coletivas, coordenam programas e serviços em saúde, efetuam perícias, auditorias e sindicâncias médicas, elaboram documentos e difundem conhecimentos da área médica.

- ### Condições Gerais de Exercício

- Médicos clínicos os cargos dessa família CBO exercem suas funções em setores cujas atividades referem-se a saúde e serviços sociais, ensino, pesquisa e desenvolvimento. De modo geral atuam por conta própria, na condição de autônomos, sem supervisão permanente. Organizam-se individualmente e em equipe de trabalho, desenvolvendo as atividades em ambientes fechados, em horários de trabalho irregulares. Exceção feita profissionais que atuam no Programa de Estratégia de Saúde da Família, onde exercem suas funções como empregados, com supervisão ocasional e cumprem carga horária semanal prevista em Portaria específica. Podem trabalhar em posições desconfortáveis durante longos períodos e, devido à natureza e nível de responsabilidade próprio da função, podem estar sujeitos a estresse constante. Em algumas ocupações os profissionais podem estar sujeitos a ação de **materiais tóxicos, químicos, radioativos e biológicos.**

- ## CBO 223710 – Nutricionista
- ### Descrição Sumária:

Prestam assistência nutricional a indivíduos e coletividades (sadios e enfermos); planejam, organizam, administram e avaliam unidades de alimentação e nutrição; efetuam controle higiênico-sanitário; participam de programas de educação nutricional; podem estruturar e gerenciar serviços de atendimento ao consumidor de indústrias de alimentos e ministrar cursos. Atuam em conformidade ao manual de boas práticas.

- ### Formação e Experiência:

Para ingressar nessas ocupações é necessário que o trabalhador tenha curso superior em nutrição. Dietistas são os profissionais formados pela Faculdade de Higiene dos Alimentos.

- ### Condições Gerais de Exercício:

Atuam nas seguintes áreas: alimentação coletiva (creches, escolas, cesta básica, hotelaria, convênio-refeição, restaurante comercial, restaurante industrial e concessionárias, gastronomia, catering);

clínica (hospitais, spa, lactário, ambulatório, consultório, banco de leite, atendimento domiciliar, estética); esporte; saúde coletiva (programas institucionais, unidades primárias de saúde); saúde pública (vigilância sanitária e vigilância institucional); marketing (atendimento ao consumidor e cozinha experimental); indústria de alimentos; consultoria/assessoria. São assalariados, organizam-se em equipe multi e interdisciplinar e trabalham sem supervisão. Executam seu trabalho em ambiente fechado e em horário diurno, podendo, em alguns casos, trabalhar em horário irregular. Estão sujeitos a trabalho sob pressão, levando à situação de estresse, a posições desconfortáveis durante longos períodos e podem ser expostos a ruído intenso, altas temperaturas, risco ambiental e insalubridade. Existe uma nova tendência em nutrição, que diz respeito à nutrição animal. Trata-se de um trabalho multiprofissional, ligado à gestão ambiental, onde o nutricionista trabalha com questões do desperdício e de rastreabilidade de doenças animais, principalmente de animais silvestres.

• CBO 4121- Operadores de equipamentos de entrada e transmissão de dados

• Descrição Sumária

Organizam a rotina de serviços e realizam entrada e transmissão de dados, operando teleimpressoras e microcomputadores; registram e transcrevem informações, operando máquinas de escrever; atendem necessidades do cliente interno e externo. Supervisionam trabalho e equipe e negociam serviço com cliente.

• Formação e Experiência

O exercício dessas ocupações requer escolaridade de ensino médio e curso básico de profissionalização de até duzentas horas/aula. o pleno desempenho das atividades ocorre com menos de um ano de experiência e no caso de supervisor, após dois anos.a(s) ocupação(ões) elencada(s) nesta família ocupacional demanda formação profissional para efeitos do cálculo do número de aprendizes a serem contratados pelos estabelecimentos, nos termos do artigo 429 da consolidação das leis do trabalho - clt, exceto os casos previstos no art. 10 do decreto 5.598/2005.

• Condições Gerais de Exercício

• Trabalham em serviços de apoio administrativo nos mais variados ramos de atividade, em serviços de informática e de correio. São assalariados, com carteira assinada, exceto o digitador que pode trabalhar por conta própria. Atuam de forma individual e em equipe, sob supervisão, em ambiente fechado e em sistema de rodízio de turnos.

• CBO 3722-05 - Operador de rede de teleprocessamento

• Descrição Sumária

Operam e monitoram sistemas de comunicação em rede, preparam equipamentos emeios de comunicação, cuidam da segurança operacional por meio de procedimentosespecíficos e realizam atendimento ao usuário.

- ## Formação e Experiência

Para o exercício dessas ocupações requer-se formação de nível médio. para as ocupa ções de operador de teleprocessamento requer-se, adicionalmente, cursos de especialização de aproximadamente quatrocentas horas/aula. a(s) ocupação(ões) elencada(s)nesta família ocupacional demanda formação profissional para efeitos do cálculo do número de aprendizes a serem contratados pelos estabelecimentos, nos termos do artigo 429 da consolidação das leis do trabalho - clt, exceto os casos previstos no art.10 do decreto 5.598/2005.

- ## Condições Gerais de Exercício

- Trabalham em empresas de informática e departamentos de informática de empresas de diversos ramos de atividade e em entidades que atuam com populações residentes em áreas remotas. São assalariados, com carteira assinada, pela empresa onde atuam ou empresa terceirizada. trabalham em equipe, sob supervisão, em ambiente fechado, em diferentes **regimes de horário de trabalho diurno, noturno, rodízio de turnos e horários irregulares.**

- # CBO 4222 - Operadores de telefonia

- ## Descrição Sumária

Operam equipamentos, atendem, transferem, cadastram e completam chamadas telefônicas locais, nacionais e internacionais, comunicando-se formalmente em português e/ou línguas estrangeiras. Auxiliam o cliente, fornecendo informações e prestando serviços gerais. Podem treinar funcionários e avaliar a qualidade de atendimento do operador, identificando pontos de melhoria.

- ## Formação e Experiência

Essas ocupações são exercidas por trabalhadores com escolaridade de nível médio, exceto a de telefonista para a qual é requerido, no mínimo, o ensino fundamental. A formação profissional ocorre com a prática de um a dois anos, no local de trabalho. pode-se demandar aprendizagem profissional para a(s) ocupação(ões) elencada(s) nesta família ocupacional, exceto os casos previstos no art. 10 do decreto 5.598/2005.

- ## Condições Gerais de Exercício

- Os trabalhadores dessas ocupações têm vínculo de trabalho assalariado, com carteira assinada. atuam, predominantemente, em empresas de saúde, serviços sociais, correios e telecomunicações. Trabalham com supervisão, em ambientes fechados, revezamento de turnos, com compromisso de manter sigilo. o trabalho é exercido sob pressão e com exposição a ruído intenso, levando à situação de estresse. Os trabalhadores estão sujeitos a lesões buco-maxilar-auditivas e por

esforços repetitivos. A transferência de funções de telefonistas para sistemas automatizados de mensagens, bem como a migração para outras ocupações, como telemarketing, tende a diminuir o emprego nessa ocupação.

- ## CBO 5211- Operadores do comércio em lojas e mercados

- ### Descrição Sumária

Vendem mercadorias em estabelecimentos do comércio varejista ou atacadista, auxi liando os clientes na escolha. registram entrada e saída de mercadorias. promovem avenda de mercadorias, demonstrando seu funcionamento, oferecendo-as para degus tação ou distribuindo amostras das mesmas. informam sobre suas qualidades e van tagens de aquisição. expõem mercadorias de forma atrativa, em pontos estratégicosde vendas, com etiquetas de preço. prestam serviços aos clientes, tais como troca demercadorias; abastecimento de veículos; aplicação de injeção e outros serviços corre latos. fazem inventário de mercadorias para reposição. elaboram relatórios de vendas,de promoções, de demonstrações e de pesquisa de preços.

- ### Formação e Experiência

Em geral, para o exercício das ocupações de atendente de farmácia, demonstrador de mercadorias, promotor de vendas, repositor de mercadorias, vendedor de comércio varejista e vendedor atacadista, requer-se do ensino fundamental ao ensino médio, podendo o mesmo variar de acordo com a ocupação, e quarta série do ensino fundamental para frentista. o tempo médio para o desempenho profissional é heterogêneo: três a quatro anos para vendedores, um a dois anos para atendente de farmácia e menos de um ano para as demais ocupações. a(s) ocupação(ões) elencada(s)nesta família ocupacional demanda formação profissional para efeitos do cálculo do número de aprendizes a serem contratados pelos estabelecimentos, nos termos do artigo 429 da consolidação das leis do trabalho - clt, exceto os casos previstos no art. 10 do decreto 5.598/2005.

- ### Condições Gerais de Exercício

- Trabalham como assalariados, com carteira assinada ou como autônoma, em empresas comerciais. o ambiente de trabalho é fechado, exceto para o frentista que atua, geral mente, a céu aberto. Trabalham individualmente, com supervisão permanente ou ocasional, **em horários diurnos, noturnos e em rodízio de turnos.** Permanecem em pé, por longos períodos. Podem estar expostos a ruídos, temperaturas variadas e **material tóxico.**

-

- ## CBO 5174 - Porteiros, vigias e afins

- ### Descrição Sumária

Zelam pela guarda do patrimônio e exercem a vigilância de fábricas, armazéns, residências, estacionamentos, edifícios públicos, privados e outros estabelecimentos, percorrendo-os sistematicamente e inspecionando suas dependências, para evitar incêndios, roubos, entrada de pessoas estranhas e outras anormalidades; controlam fluxo de pessoas, identificando, orientando e encaminhando-as para os lugares desejados; recebem hóspedes em hotéis; escoltam pessoas e mercadorias; fazem manutenções simples nos locais de trabalho.

- ## Formação e Experiência

O acesso a essas ocupações requer ensino fundamental. os hotéis e as empresas de vigilância oferecem treinamentos ou recrutam os trabalhadores no mercado de trabalho e em instituições de formação profissional. a(s) ocupação(ões) elencada(s) nesta família ocupacional demanda formação profissional para efeitos do cálculo do número de aprendizes a serem contratados pelos estabelecimentos, nos termos do artigo 429da consolidação das leis do trabalho - clt, exceto os casos previstos no art. 10 do decreto 5.598/2005.

- ## Condições Gerais de Exercício

Trabalham em edifícios residenciais, comerciais e industriais, hotéis, locais de diversão. Podem ser empregados de locadoras de mão-de-obra, e fazer rodízio nas ocupações de porteiro de edifício, de locais de diversão e vigia.

- # CBO 2621-05 - Produtor cultural (Produtores de espetáculos)

- ## Descrição Sumária

Implementam projetos de produção de espetáculos artísticos e culturais (teatro, dança, ópera, exposições e outros), audiovisuais (cinema, vídeo, televisão, rádio e produção musical) e multimídia. Para tanto criam propostas, realizam a pré-produção e finalização dos projetos, gerindo os recursos financeiros disponíveis para o mesmo.

- ## Formação e Experiência

Essas ocupações não demandam nível de escolaridade determinado para seu desempenho, sendo possível que sua aprendizagem ocorra na prática. Seguindo a tendência de profissionalização que vem ocorrendo na área das artes, contudo, pode-se afirmar que, cada vez mais será desejável que os profissionais apresentem escolaridade de nível superior.

- ## Condições Gerais de Exercício

- Trabalham principalmente em atividades culturais, recreativas, desportivas, em empresas públicas ou privadas, como empregados ou prestadores de serviços. As habilidades de pesquisa,

organização, supervisão e de relacionamento interpessoal são importantes para o exercício das suas atividades, as quais se desenvolvem predominantemente em equipes e em horários irregulares.

- **CBO 2241- Profissionais da educação física**

- **Descrição Sumária**

Desenvolvem, com crianças, jovens e adultos, atividades físicas; ensinam técnicas despor tivas realizam treinamentos especializados com atletas de diferentes esportes; instruem lhes acerca dos princípios e regras inerentes a cada um deles; avaliam e supervisionam o preparo físico dos atletas; acompanham e supervisionam as práticas desportivas; elaboram informes técnicos e científicos na área de atividades físicas e do desporto.

- **Formação e Experiência**

O exercício das ocupações da família requer formação superior em educação física, com registro no conselho regional de educação física. no mercado de trabalho, cresce o número de profissionais portadores de cursos de especialização e pós-graduação. O exercício pleno das atividades varia conforme a ocupação, entre um e quatro anos.

- **Condições Gerais de Exercício**

- Os profissionais prestam serviços no campo dos exercícios físicos com objetivos educacionais, de saúde e de desempenho esportivo. podem trabalhar em academias e escolas de esporte, clubes e hotéis, clínicas médicas e fisioterápicas, em atendimentos domiciliares, em órgãos da administração pública direta, etc, como empregados com carteira ou como autônomos. Desenvolvem seu trabalho de forma individual, nos mais variados ambientes, em horários irregulares. em algumas atividades, alguns profissionais podem trabalhar sob condições especiais, por exemplo, em posições desconfortáveis por período prolongado, sob pressão, sujeitos a mudanças climáticas e intempéries.

- **CBO 331105 - Professor de nível médio na educação infantil**
- **Descrição Sumária:**

Ensinam e cuidam de alunos na faixa de zero a seis anos; orientam a construção do conhecimento; elaboram projetos pedagógicos; planejam ações didáticas e avaliam o desempenho dos alunos. Preparam material pedagógico; organizam o trabalho. No desenvolvimento das atividades, mobilizam um conjunto de capacidades comunicativas.

- **Formação e Experiência:**

Para professores de nível médio na educação infantil requer-se escolaridade de ensino médio, acrescida de curso técnico de formação para o magistério. Para a ocupação de auxiliar de desenvolvimento infantil, é desejável escolaridade de ensino médio completo com aprendizado no local de trabalho, sob orientação da equipe escolar.

- **Condições Gerais de Exercício:**

Trabalham em instituições de ensino das esferas pública e privada. Atuam de forma individual, com supervisão permanente, em ambientes fechados e a céu aberto, no período diurno.

- **CBO 231210 - Professor de nível superior do ensino fundamental (primeira a quarta série)**
- **Descrição Sumária:**

Ministram aulas (comunicação e expressão, integração social e iniciação às ciências) nas quatro primeiras séries do ensino fundamental. Preparam aulas; efetuam registros burocráticos e pedagógicos; participam na elaboração do projeto pedagógico; planejam o curso de acordo com as diretrizes educacionais. Atuam em reuniões administrativas e pedagógicas; organizam eventos e atividades sociais, culturais e pedagógicas. Para o desenvolvimento das atividades utilizam constantemente capacidades de comunicação.

- **Formação e Experiência:**

O exercício dessas ocupações requer escolaridade de nível superior na área específica de educação e concurso público, no caso da rede pública.

- **Condições Gerais de Exercício:**

Desenvolvem trabalho com crianças, adolescentes e adultos, em comunidades com contextos culturais e sociais diversificados, em escolas e instituições de ensino das redes federal, estadual, municipal e privada,ONGs, etc., como estatutários ou empregados com carteira assinada. Trabalham de forma individual e em equipes, sob supervisão, predominantemente em zonas urbanas, tanto em espaços especialmente destinados ao ensino, como em ambientes improvisados, em horários regulares e variáveis. Em algumas atividades, alguns profissionais podem estar sujeitos a condições especiais de trabalho, como permanência em posições desconfortáveis por períodos prolongados, exposição a ruídos e ao desgaste proveniente do uso intensivo da voz.

- **CBO 231110 - Professor de nível superior na educação infantil (zero a três anos)**
- **Descrição Sumária:**

Promovem educação e a relação ensino-aprendizagem de crianças de até seis anos; cuidam de alunos; planejam a prática educacional e avaliam as práticas pedagógicas. Organizam atividades; pesquisam; interagem com a família e a comunidade e realizam tarefas administrativas.

- **Formação e Experiência:**

O exercício dessas ocupações requer formação de nível superior na área de educação e concurso público, no caso da rede pública.

- **Condições Gerais de Exercício:**

Exercem suas funções em instituições de ensino, públicas ou privadas. São estatutários ou contratados na condição de trabalhadores assalariados, com carteira assinada; trabalham de forma individual, com supervisão ocasional, em ambientes fechados, geralmente no período diurno. Podem estar expostos à ação de ruído intenso, no desenvolvimento de algumas atividades.

- **Professor de nível superior na educação infantil (quatro a seis anos)**
- **Descrição Sumária:**

Promovem educação e a relação ensino-aprendizagem de crianças de até seis anos; cuidam de alunos; planejam a prática educacional e avaliam as práticas pedagógicas. Organizam atividades; pesquisam; interagem com a família e a comunidade e realizam tarefas administrativas.

- **Formação e Experiência:**

O exercício dessas ocupações requer formação de nível superior na área de educação e concurso público, no caso da rede pública.

- **Condições Gerais de Exercício:**

Exercem suas funções em instituições de ensino, públicas ou privadas. São estatutários ou contratados na condição de trabalhadores assalariados, com carteira assinada; trabalham de forma individual, com supervisão ocasional, em ambientes fechados, geralmente no período diurno. Podem estar expostos à ação de ruído intenso, no desenvolvimento de algumas atividades.

- **CBO 2321 - Professores do ensino médio**

- **Descrição Sumária**

Ministram aulas teóricas e práticas no ensino médio, em escolas da rede pública e privada; acompanham a produção da área educacional e cultural; planejam o curso, a disciplina e o projeto pedagógico; avaliam o processo de ensino-aprendizagem; preparam aulas e participam de atividades institucionais. para o desenvolvimento das atividades é mobilizado um conjunto de capacidades comunicativas.

- **Formação e Experiência**

O exercício dessas ocupações requer formação de nível superior específica, confor me a área de atuação. na rede pública de ensino requer-se concurso público.

- ## Condições Gerais de Exercício

Trabalham com clientelas de diferentes faixas etárias, com predomínio de adolescentes, oriundas de comunidades com diferentes contextos culturais e sociais. Atuam em escolas ou instituições de ensino das redes públicas federal, estadual, municipal, da rede privada e ong. Trabalham em zonas urbanas, como estatutários ou empregados registrados em carteira. Desenvolvem suas atividades de forma individual e em equipe, com supervisão ocasional, em locais próprios à atividade de ensino. Em algumas atividades, podem estar expostos aos efeitos do ruído intenso, à fadiga das cordas vocais e ao estresse do trabalho sob pressão.

- # CBO 234520 - Professor de ensino superior na área de prática de ensino (Professor de ensino superior)

- ## Descrição Sumária:

Ensinam, articulando o processo de ensino-aprendizagem na formação de profissionais da educação; planejam atividades relativas a cursos e pesquisas; realizam pesquisas científicas sobre o campo educacional; supervisionam formação pedagógica em estágios; orientam alunos; avaliam o trabalho acadêmico científico; coordenam atividades de ensino, pesquisa e extensão. Produzem material de trabalho; prestam atendimento às demandas da comunidade na área da educação escolar e não-escolar (educação formal e informal); participam de atividades administrativas, atualizam-se na área e comunicam-se oralmente e por escrito.

- ## Formação e Experiência:

Os ocupações requerem, no mínimo, o ensino superior completo, sendo importante a posse de títulos de pós-graduação ou especialização na área. É comum o ingresso e a progressão na carreira por intermédio de concursos, principalmente, na área pública. Dos titulares das ocupações espera-se que tenham experiência anterior de, pelo menos, cinco anos.

- ## Condições Gerais de Exercício:

Os profissionais dessa família ocupacional exercem suas funções em instituições cujas atividades referem-se a ensino superior, cultura e pesquisa. Desenvolvem suas atividades na condição de trabalhadores assalariados, com carteira assinada; organizam-se em equipes de trabalho; atuam com supervisão ocasional, em ambientes fechados e em horários irregulares. Algumas vezes, podem trabalhar em posições desconfortáveis durante períodos de tempo.

- # CBO 2332 - Instrutores de ensino profissional

- ## Descrição Sumária

Planejam e desenvolvem situações de ensino e aprendizagem voltadas para a qualificação profissional de jovens e adultos orientando-os nas técnicas específicas da área em questão. avaliam processo ensino-aprendizagem; elaboram material pedagógico; sistematizam estudos, informações e

experiências sobre a área ensinada; garantem segurança, higiene e proteção ambiental nas situações de ensino aprendizagem; fazem registros de documentação escolar, de oficinas e de labora tórios. Podem prestar serviços à comunidade. No desenvolvimento das atividades mobilizam capacidades comunicativas.

- **Formação e Experiência**

O exercício dessas ocupações requer, no mínimo, curso técnico profissionalizante na área de atuação mais curso de complementação pedagógica de nível superior.

- **Condições Gerais de Exercício**

Trabalham em escolas e instituições de formação profissional de caráter privado e público, bem como em sindicatos, ong, etc., como estatutário ou assalariado com carteira assinada. Atuam no ensino das áreas comercial, industrial, agroflorestal e de serviços, com alunos de diversas faixas etárias, facilitando o aprendizado e fornecendo os meios para o exercício das atividades práticas de uma dada profissão em locais variados, como salas de aulas, oficinas, no campo, etc. desenvolvem suas atividades em equipe, com supervisão permanente, em rodízios de turnos ou em horários irregulares.

-

- **CBO 2612 - Profissionais da informação**

- **Descrição Sumária**

Disponibilizam informação em qualquer suporte; gerenciam unidades como bibliotecas, centros de documentação, centros de informação e correlatos, além de redes e sistemas de informação. Tratam tecnicamente e desenvolvem recursos informacionais; disseminam informação com o objetivo de facilitar o acesso e geração do conhecimento; desenvolvem estudos e pesquisas; realizam difusão cultural; desenvolvem ações educativas. Podem prestar serviços de assessoria e consultoria.

- **Formação e Experiência**

O exercício dessas ocupações requer bacharelado em biblioteconomia e documentação. a formação é complementada com aprendizado tácito no local de trabalho e cursos de extensão.

- **Condições Gerais de Exercício**

- Trabalham em bibliotecas e centros de documentação e informação na administração pública e nas mais variadas atividades do comércio, indústria e serviços, com predominância nas áreas de educação e pesquisa. Trabalham como assalariados, com carteira assinada ou como autônoma, de forma individual ou em equipe por projetos, com supervisão ocasional, em ambientes

fechados e com rodízio de turnos. Podem executar suas funções tanto de forma presencial como a distância. Eventualmente, trabalham em posições desconfortáveis durante longos períodos e sob pressão, levando à situação de estresse. As condições de trabalho são heterogêneas, variando desde locais com pequeno acervo e sem recursos informacionais a locais que trabalham com tecnologia de ponta.consulte3711 - técnicos em biblioteconomia.

- **CBO 2133 - Profissionais das ciências atmosféricas e espaciais e de astronomia**

- **Descrição Sumária**

Realizam pesquisas científicas para prognosticar fenômenos meteorológicos, astronômicos e de geofísica espacial; obtêm e tratam dados; disseminam informações por meio de trabalhos, teses, publicações, eventos, etc.; desenvolvem sistemas computacionais, instrumentação científica e gerenciam projetos nas suas áreas.

- **Formação e Experiência**

Para o exercício dessas ocupações requer-se curso superior completo e experiência profissional que varia de um a dois anos para o meteorologista, de três a quatro anos para o geofísico espacial e mais de cinco anos para o astrônomo. a formação desses profissionais é diversificada. em gastronomia existe apenas uma escola no país, no Rio de janeiro (ufrj), com curso de graduação. Profissionais com formação em física atuam como astrônomos qualificando-se em cursos de pós graduação em astronomia. Na área de meteorologia existe um número maior de universidades que formam profissionais em cursos de graduação e formações afins, como a geografia, que oferece cursos de especialização na área.

- **Condições Gerais de Exercício**

- Devido às suas características de pesquisas, o astrônomo atua apenas em instituições públicas como institutos de pesquisa e universidades. O meteorologista e o geofísico espacial podem atuar tanto na iniciativa privada, em empresas de eletricidade, empresas aéreas, empresas e indústrias que trabalham com alimentos perecíveis, etc., quanto no setor público, em secretarias estaduais e municipais de agricultura, planejamento, urbanismo, educação, saúde, transporte; institutos de pesquisa etc. são empregados assalaria dos, variando a autonomia no trabalho conforme a ocupação. trabalham em equipe, em ambiente fechado e em horário diurno, exceto o meteorologista que faz revezamento de turnos e, eventualmente, trabalha sob pressão, levando a situações de estresse.

- **CBO 2525 - Profissionais de administração econômico-financeira**

- **Descrição Sumária**

Administram fundos e carteiras de investimentos em instituições financeiras. Desenvolvem, implantam e administram produtos e serviços bancários. Analisam operações de crédito e de cobrança e operacionalizam contratos de financiamento e/ou empréstimos. Controlam recursos para crédito obrigatório e gerenciam cobranças. Preparam e consolidam informações gerenciais e econômico-financeiras. Relatam aos setores e clientes do banco, oralmente ou por escrito, a situação dos produtos e serviços bancários.

- **Formação e Experiência**

O exercício dessas ocupações requer nível superior completo e curso de especialização na área com duração de até duzentas horas/aula. O exercício pleno das ocupações se dá após três ou quatro anos de experiência.

- **Condições Gerais de Exercício**

- O trabalho é exercido em instituições financeiras, seguros e previdência privada, ad ministração pública, defesa e seguridade social e outras atividades empresariais. Os profissionais são assalariados com carteira assinada. Trabalham em equipe, sob supervisão permanente. O trabalho é presencial, realizado em ambiente fechado, no período diurno. As atividades são executadas sob pressão, levando à situação de estresse constante.
-
-
-
- **CBO 211205 – Estatístico (Profissionais de Estatística)**
- **Descrição Sumária:**

Desenham amostras; analisam e processam dados; constroem instrumentos de coleta de dados; criam banco de dados; desenvolvem sistemas de codificação de dados; planejam pesquisa; comunicam-se oralmente e por escrito.

- **Formação e Experiência:**

Para o acesso às ocupações requer-se curso superior completo, sendo desejável curso superior de tecnologia (tecnólogo), cursos de especialização ou de pós-graduação. O exercício pleno das atividades, em média, ocorre no período que pode variar de três a quatro anos de experiência profissional.

- **Condições Gerais de Exercício:**

Atuam em três grandes áreas da estatística: a industrial, a aplicada às ciências biológicas e da saúde e a aplicada às ciências humanas e sociais. No exercício das suas atividades se utilizam da estatística como ferramenta de trabalho. Podem trabalhar em institutos de pesquisa, planejamento e estatística, instituições de ensino, empresas e fundações públicas ou privadas. Organizam-se em equipe e também de forma individual (Estatístico teórico). Atuam com supervisão permanente e

também ocasional, dependendo da ocupação. Trabalham em ambientes fechados, no período diurno. Em algumas de suas atividades, podem trabalhar em posições desconfortáveis por longos períodos, bem como estar expostos à radiação dos monitores de computadores.

- **CBO 2524 - Profissionais de recursos humanos**
- **Descrição Sumária**

Administram pessoal e plano de cargos e salários; promovem ações de treinamento e de desenvolvimento de pessoal. Efetuam processo de recrutamento e de seleção, geram plano de benefícios e promovem ações de qualidade de vida e assistência aos empregados. Administram relações de trabalho e coordenam sistemas de avaliação de desempenho. No desenvolvimento das atividades, mobilizam um conjunto de capacidades comunicativas.

- **Formação e Experiência**

O exercício dessa ocupação requer escolaridade de ensino superior. O desempenho pleno das funções ocorre após o período de cinco anos de experiência profissional.

- **Condições Gerais de Exercício**

- Exercem suas funções nos departamentos de recursos humanos de empresas. De modo geral são contratados na condição de empregados com carteira assinada, podendo, na sua minoria, atuar como prestadores de serviços autônomos. Trabalham de forma individual, sob supervisão, em ambiente fechado, no período diurno. podem estar sujeitos estresse, devido a trabalho sob pressão.
-
-
- **CBO 2531- Profissionais de publicidade**
- **Descrição Sumária**

Estruturam estratégias de projeto; pesquisam o quadro econômico, político, social e cultural; analisam mercado; desenvolvem propaganda e promoções; implantam ações de relações públicas e assessoria de imprensa; vendem produtos, serviços e conceitos. No desenvolvimento das atividades é mobilizado um conjunto de capa cidades comunicativas.

- **Formação e Experiência**

Para o exercício dessas ocupações requer-se curso superior ou pós-graduação em relações públicas e áreas correlatas. o pleno exercício das atividades ocorre após um ou dois anos de experiência. É desejável o domínio de línguas estrangeiras.

- **Condições Gerais de Exercício**

- Exercem suas atividades em empresas de qualquer setor de atividade econômica, como a indústria, o comércio, prestação de serviços, agropecuária e a administração pública. Trabalham majoritariamente como autônomos ou associados a agências de publicidade e a institutos de pesquisa de mercado. Trabalham em período diurno, sem supervisão. Em algumas atividades podem trabalhar sob pressão, oque pode ocasionar estresse.

- ## CBO 2513-Profissionais em pesquisa e análise geográfica

- **Descrição Sumária**

Estudam a organização espacial por meio da interpretação e da interação dos aspectos físicos e humanos; regionalizam o território em escalas que variam do local ao global; avaliam os processos de produção do espaço, subsidiando o ordenamento territorial; participam do planejamento regional, urbano, rural, ambiental e da formulação de políticas de gestão do território; procedem estudos necessários ao estabelecimento de bases territoriais; emitem laudos e pareceres técnicos; monitoram uso e ocupação da terra, vistoriam áreas em estudo, estudam a pressão antrópica e diagnosticam impactos e tendências.

- **Formação e Experiência**

Para atuar como geógrafo requer-se bacharelado em geografia e registro no CREA. Há tendência ao aumento de exigência de qualificação, como especialização, mestrado e doutorado.

- **Condições Gerais de Exercício**

Atuam em institutos de estatística, órgãos de planejamento territorial municipal, estaduais e federais, empresas de consultoria que desenvolvem trabalhos aplicados à agricultura, pecuária e indústria. Prestam serviços a organismos internacionais. Trabalham como assalariados ou autônomos, com ou sem supervisão.

- ## CBO 2394 - Programadores, avaliadores e orientadores de ensino

- **Descrição Sumária**

Implementam, avaliam, coordenam e planejam o desenvolvimento de projetos pedagógicos/instrucionais nas modalidades de ensino presencial e/ou a distância, aplicando metodologias e técnicas para facilitar o processo de ensino e aprendizagem. Atuam em cursos acadêmicos e/ou corporativos em todos os níveis de ensino para atender as necessidades dos alunos, acompanhando e avaliando os processos educacionais. Viabilizam o trabalho coletivo, criando e organizando mecanismos de participação em programas e projetos educacionais, facilitando o processo comunicativo entre a comunidade escolar e as associações a ela vinculadas.

- ## Formação e Experiência

O exercício dessas ocupações requer curso superior na área de educação ou áreas correlatas. O desempenho pleno das atividades ocorre após três ou quatro anos de exercício profissional.

- ## Condições Gerais de Exercício

Atuam em atividades de ensino nas esferas públicas e privadas. São estatutários ou empregados com carteira assinada; trabalham tanto individualmente como em equipe interdisciplinar, com supervisão ocasional, em ambientes fechados e em horários diurno e noturno. Em algumas atividades podem trabalhar sob pressão, levando-os à situação de estresse.

- # CBO 2515 - Psicólogos e psicanalistas

- ## Descrição Sumária

Estudam, pesquisam e avaliam o desenvolvimento emocional e os processos mentais e sociais de indivíduos, grupos e instituições, com a finalidade de análise, tratamento, orientação e educação; diagnosticam e avaliam distúrbios emocionais e mentais e de adaptação social, elucidando conflitos e questões e acompanhando o(s) paciente(s) durante o processo de tratamento ou cura; investigam os fatores inconscientes do comportamento individual e grupal, tornando-os conscientes; desenvolvem pesquisas experimentais, teóricas e clínicas e coordenam equipes e atividades de área e afins.

- ## Formação e Experiência

Para os trabalhadores dessa família é exigido o nível superior completo e experiência profissional que varia segundo a formação. Para os psicólogos, de modo geral, pede-se de um a quatro anos, como é o caso do psicólogo clínico. Para o psicanalista é necessário, no mínimo, cinco anos de experiência. Os cursos de qualificação também variam de cursos básicos de duzentas a quatrocentas horas/aula, como no caso do psicólogo hospitalar, mais de quatrocentas horas/aula para os psicólogos jurídicos, psicanalistas e neuropsicólogos, até cursos de especialização para os psicólogos clínicos e sociais. A formação desses profissionais é um conjunto de atividades desenvolvidas por eles, mas os procedimentos são diferentes quanto a aspectos formais relacionados às instituições que os formam.

- ## Condições Gerais de Exercício

Os profissionais dessa família ocupacional atuam, principalmente, em atividades liga das à saúde, serviços sociais e pessoais e educação. Podem trabalhar como autônomos e/ou com carteira assinada, individualmente ou em equipes. É comum os psicólogos clínico, hospitalar, social e neuropsicólogos trabalharem com supervisão. Têm como local de trabalho ambiente fechado ou, no caso dos neuropsicólogos e psicólogos jurídicos, pode ser a céu aberto. Os psicólogos clínicos, sociais e os psicanalistas, eventualmente, trabalham em horários irregulares. Alguns deles trabalham

sob pressão, em posições desconfortáveis durante longos períodos, confinados (psicólogos clínicos e sociais) e expostos à radiação (neuropsicólogos) e ruídos intensos. A ocupação psicanalista não é uma especialização, é uma formação que segue princípios, processos e procedimentos definidos pelas instituições reconhecidas internacionalmente, podendo o psicanalista ter diferentes formações como: psicólogo, psiquiatra, médico, filósofo, etc.

- **CBO 213205 – Químico**
- **Descrição Sumária:**

Realizam ensaios, análises químicas e físico-químicas, selecionando metodologias, materiais, reagentes de análise e critérios de amostragem, homogeneizando, dimensionando e solubilizando amostras. Produzem substâncias, desenvolvem metodologias analíticas, interpretam dados químicos, monitoram impacto ambiental de substâncias, supervisionam procedimentos químicos, coordenam atividades químicas laboratoriais e industriais.

- **Formação e Experiência:**

Para o acesso às ocupações requer-se curso superior em nível de tecnologia ou bacharelado completo na área de Química e afins, sendo que há titulares que são pós-graduados e/ou especializados. Em média, o exercício pleno das atividades nas ocupações demanda quatro anos de experiência.

- **Condições Gerais de Exercício:**

No setor industrial estão presentes na extração de minérios, petróleo e gás natural, na fabricação de alimentos e bebidas, de produtos químicos, na produção de combustíveis diversos. Também são empregados na agropecuária e no setor de serviços como, por exemplo, na captação, purificação e distribuição de água e na proteção ambiental. A maior oferta de vagas encontra-se na esfera privada, na qual o vínculo de trabalho mais frequente é como empregado com carteira assinada. Além das empresas, estão presentes em instituições de pesquisa e nas universidades. Neste caso, são classificados como pesquisadores ou professores. Costumam desenvolver suas atividades em equipes, analisando e manipulando substâncias em escala piloto e em laboratórios. Eventualmente, em algumas atividades pode ocorrer **exposição a materiais tóxico**s, radiação, ruído intenso e altas temperaturas.

- **CBO 422105 - Recepcionista, em geral (Recepcionaistas)**
- **Descrição Sumária:**

Recepcionam e prestam serviços de apoio a clientes, pacientes, hóspedes, visitantes e passageiros; prestam atendimento telefônico e fornecem informações em escritórios, consultórios, hoteis, hospitais, bancos, aeroportos e outros estabelecimentos; marcam entrevistas ou consultas e recebem clientes ou visitantes; averiguam suas necessidades e dirigem ao lugar ou a pessoa procurados;

agendam serviços, reservam (hotéis e passagens) e indicam acomodações em hotéis e estabelecimentos similares; observam normas internas de segurança, conferindo documentos e idoneidade dos clientes e notificando seguranças sobre presenças estranhas; fecham contas e estadas de clientes. Organizam informações e planejam o trabalho do cotidiano.

- **Formação e Experiência:**

Essas ocupações requerem o ensino médio completo, exceto o recepcionista de hotel que tem como pré-requisito o ensino superior incompleto. É desejável curso básico de qualificação de até duzentas horas/aula e de um a dois anos de experiência profissional para o recepcionista, em geral. A(s) ocupação(ões) elencada(s) nesta família ocupacional demanda formação profissional para efeitos do cálculo do número de aprendizes a serem contratados pelos estabelecimentos, nos termos do artigo 429 da Consolidação das Leis do Trabalho - CLT, exceto os casos previstos no art. 10 do Decreto 5.598/2005.

- **Condições Gerais de Exercício:**

Trabalham nas atividades de saúde e serviços sociais, alojamento e alimentação, transporte aéreo e atividades recreativas, culturais e desportivas. São empregados com carteira assinada, trabalham em equipe multidisciplinar, em ambientes fechados e em horários que variam conforme a ocupação diurno para os recepcionistas de seguro saúde, revezamento de turnos para o recepcionista de consultório e o recepcionista de hotel, e horários irregulares para o recepcionista, em geral. Também varia o grau de autonomia, podendo ser com supervisão permanente para o recepcionista de consultório e para o recepcionista de hotel, e com supervisão ocasional para os restantes. Verifica-se o crescimento da importância da hotelaria nos hospitais de ponta. Com isso, tende a crescer o número de empregados no setor de recepção que, além de ainda estar muitas vezes acoplado ao sistema de internação, assume cada vez mais funções idênticas às dos recepcionistas de um hotel cinco estrelas.

- **CBO 410105 - Supervisor administrativo**
- **Descrição Sumária:**

Supervisionam rotinas administrativas em instituições públicas e privadas, chefiando diretamente equipe de escriturários, auxiliares administrativos, secretários de expediente, operadores de máquina de escritório e contínuos. Coordenam serviços gerais de malotes, mensageiros, transporte, cartório, limpeza, terceirizados, manutenção de equipamento, mobiliário, instalações etc; administram recursos humanos, bens patrimoniais e materiais de consumo; organizam documentos e correspondências; gerenciam equipe. Podem manter rotinas financeiras, controlando fundo fixo (pequeno caixa), verbas, contas a pagar, fluxo de caixa e conta bancária, emitindo e conferindo notas fiscais e recibos, prestando contas e recolhendo impostos.

- **Formação e Experiência:**

Para ingressar nessa ocupação é exigido o ensino médio completo e três a quatro anos de experiência profissional em trabalhos administrativos. A(s) ocupação(ões) elencada(s) nesta família ocupacional demanda formação profissional para efeitos do cálculo do número de aprendizes a serem contratados pelos estabelecimentos, nos termos do artigo 429 da Consolidação das Leis do Trabalho - CLT, exceto os casos previstos no art. 10 do Decreto 5.598/2005.

- **Condições Gerais de Exercício:**

Esses trabalhadores atuam nas mais diversas áreas de empresas públicas ou privadas. São assalariados celetistas ou estatutários. Trabalham em equipe, com supervisão ocasional, em ambiente fechado e em horário diurno. Eventualmente, trabalham em posições desconfortáveis durante longos períodos.

- ## CBO 7102 - Supervisores da construção civil

- **Descrição Sumária**

Supervisionam equipes de trabalhadores da construção civil que atuam em usinas de concreto, canteiros de obras civis e ferrovias. Elaboram documentação técnica e controlam recursos produtivos da obra (arranjos físicos, equipamentos, materiais, insumos e equipes de trabalho). controlam padrões produtivos da obra tais como inspeção da qualidade dos materiais e insumos utilizados, orientação sobre especificação, fluxo e movimentação dos materiais e sobre medidas de segurança dos locais e equipamentos da obra ministram o cronograma da obra.

- **Formação e Experiência**

Para o supervisor de pátio de usina de concreto requer-se ensino técnico de nível médio, experiência de três a quatro anos para o pleno desempenho das atividades. Para o fiscal de pátio de usina de concreto requer-se ensino médio mais qualificação profissional de até quatrocentas horas e o pleno desempenho ocorre após três ou quatro anos. para o exercício das demais ocupações requer-se ensino fundamental e qualificação profissional básica entre duzentas e quatrocentas horas/aula e experiência de cinco anos ou mais.a(s) ocupação(ões) elencada(s) nesta família ocupacional demanda formação profissional para efeitos do cálculo do número de aprendizes a serem contratados pelos estabelecimentos nos termos do artigo 429 da consolidação das leis do trabalho - clt, exceto os casos previstos no art. 10 do decreto 5.598/2005.

Condições Gerais de Exercício

- Atuam na indústria de construção como assalariados com carteira assinada. o trabalho é presencial, realizado em equipe, de terceiros ou próprias, sob supervisão ocasional pode ser realizado a céu aberto, em ambiente fechado - mestre (construção civil) e supervisor de usina de concreto - ou em veículos - inspetor de terraplenagem e mestre de linhas (ferrovias). Trabalham sob pressão, o que pode levá-los à situação de estresse e estão expostos a ruído intenso, **poeira e radiação solar**. O mestre (construção civil) também fica exposto a **materiais tóxicos**, assim como realiza algumas atividades em ambiente subterrâneo.

- ## CBO 760605 - Supervisor das artes gráficas (indústria editorial e gráfica)

- **Descrição Sumária:**

Supervisionam diretamente uma equipe de trabalhadores das artes gráficas, orientando- a, treinando-a em conformidade aos procedimentos técnicos, normas de qualidade, de segurança, meio ambiente e saúde. Administram o processo de produção gráfica com respeito a custos, viabilidade

de execução, fluxo de tarefas, estoque de matéria-prima e material de consumo, programação de máquinas e equipamentos, dentre outros itens. Administram metas e resultados da produção gráfica e elaboram documentos técnicos.

- **Formação e Experiência:**

Essa ocupação é exercida por trabalhadores com formação profissional de nível técnico, na área gráfica. O exercício pleno da atividade ocorre após, no mínimo, cinco anos de experiência profissional no ramo. A(s) ocupação(ões) elencada(s) nesta família ocupacional demanda formação profissional para efeitos do cálculo do número de aprendizes a serem contratados pelos estabelecimentos nos termos do artigo 429 da Consolidação das Leis do Trabalho - CLT, exceto os casos previstos no art. 10 do Decreto 5.598/2005.

- **Condições Gerais de Exercício:**

O trabalho é exercido, em grande parte, em gráficas particulares ou em grandes empresas de comunicação. O profissional é assalariado com carteira assinada e atua supervisionando uma equipe de trabalhadores, sob supervisão ocasional. O trabalho é presencial, em sistema de rodízio de turnos e em ambiente fechado. Os trabalhadores estão sujeitos a ruído intenso e permanência em posições desconfortáveis por longo período.

-

- **CBO 4102 - Supervisores de serviços financeiros, de câmbio e de controle**

- **Descrição Sumária**

Supervisionam e controlam equipe e serviços financeiros, de câmbio, bens- patrimoniais, créditos e bancários; elaboram orçamentos; efetuam e conferem pagamentos; realizam cobranças, planejando e solucionando pendências; administram almoxarifado.

- **Formação e Experiência**

Esse emprego/ocupação exige nível superior completo, curso básico de qualificação profissional de até duzentas horas/aula e de três a quatro anos de experiência.

- **Condições Gerais de Exercício**

- Os profissionais dessa família ocupacional trabalham em vários ramos de atividades, tais como construção, indústrias diversas e atividades empresariais. Normalmente, são empregados com carteira assinada com supervisão permanente, estando diretamente subordinados aos gerentes, e possuem subordinados, formando equipe. No caso dos supervisores de câmbio, existe a possibilidade de trabalharem por conta própria. Executam seu trabalho em horário diurno e em ambientes fechados. Algumas vezes trabalham sob pressão, podendo levar à situação de estresse.

- # CBO 7661 - Trabalhadores da pré-impressão gráfica

- ## Descrição Sumária

Planejam serviços de pré-impressão gráfica. realizam programação visual gráfica e editoram textos e imagens. operam processos de tratamento de imagem, montam fotolitos e imposição eletrônica. Operam sistemas de prova e copiam chapas. Gravam matrizes para fotogravura, flexografia, calcografia e serigrafia. Trabalham seguindo normas de segurança, higiene, qualidade e preservação ambiental.

- ## Formação e Experiência

Para o exercício dessas ocupações requer-se ensino médio concluído e curso de qualificação profissional de duzentas a quatrocentas horas/aula, ministrado por instituições ou escolas especializadas na área. o pleno desempenho das atividades ocorre até um ano de experiência profissional. a(s) ocupação(ões) elencada(s) nesta família ocupacional demanda formação profissional para efeitos do cálculo do número de aprendizes a serem contratados pelos estabelecimentos nos termos do artigo 429da consolidação das leis do trabalho - clt, exceto os casos previstos no art. 10 do decreto 5.598/2005.

- ## Condições Gerais de Exercício

- Atuam no segmento de edição, impressão e reprodução de gravações e em diversas atividades empresariais como empregados assalariados com carteira assinada. Estão organizados de forma individual, sob supervisão permanente, em ambiente fechado e por rodízio de **turnos (diurno/noturno)**. Trabalham sob pressão, em posições desconfortáveis por longo período de tempo e permanecem expostos a **materiais tóxicos**, radiação e ruído intenso.

-

- # CBO 7156-Trabalhadores de instalações elétricas

- ## Descrição Sumária

Planejam serviços elétricos, realizam instalação de distribuição de alta e baixa tensão. montam e reparam instalações elétricas e equipamentos auxiliares em residências, estabelecimentos industriais, comerciais e de serviços. instalam e reparam equipamentos de iluminação de cenários ou palcos.

- ## Formação e Experiência

Para o exercício dessas ocupações requer-se ensino médio e qualificação básica de duzentas horas/aula (eletricista de instalações) e quatrocentas horas/aula (eletricista de instalações de cenários e eletricista de instalação de edifícios). O desempenho pleno das atividades é atingido entre um e dois anos de prática. a(s) ocupação(ões) elencada(s)nesta família ocupacional demanda formação profissional para efeitos do cálculo do número de aprendizes a serem contratados pelos estabelecimentos nos termos do artigo429 da consolidação das leis do trabalho - CLT, exceto os casos previstos no art. 10 do decreto 5.598/2005.

- ## Condições Gerais de Exercício

- Atuam em qualquer ramo de atividade econômica que demande serviços de instalação elétrica, como teatro, construção civil, atividades industriais, comerciais e de serviços. Trabalham como assalariados ou por conta-próprios. Geralmente trabalham em equipe, com ou sem supervisão ocasional, dependendo se é empregado ou autônomo. Podem trabalhar em grandes alturas, temperaturas baixas ou elevadas, sujeitos aos riscos de trabalho com energia elétrica. Esta família não compreende 3131 - técnicos em eletricidade e eletrotécnica.

-

- # CBO 7663-Trabalhadores do acabamento gráfico

- ## Descrição Sumária

Planejam a execução do serviço, ajustam e operam máquinas de acabamento gráfico e editorial. preparam matrizes de corte e vinco, fazem gravações à máquina (hot-stam ping) e realizam manutenção produtiva dos equipamentos. Trabalham em conformidade com as estritas normas e procedimentos técnicos, de qualidade, segurança, meio ambiente e saúde.

- ## Formação e Experiência

O acesso às ocupações requer escolaridade mínima, que varia da quarta à sétima série do ensino fundamental. o operador de guilhotina e o preparador de corte e vinco são qualificados em cursos básicos profissionalizantes de duzentas horas/aula. As demais ocupações são aprendidas na prática. O desempenho pleno das atividades é alcançado com menos de um ano de prática. a(s) ocupação(ões) elencada(s) nesta família ocupacional demanda formação profissional para efeitos do cálculo do número de aprendizes a serem contratados pelos estabelecimentos nos termos do artigo429 da consolidação das leis do trabalho - clt, exceto os casos previstos no art. 10do decreto 5.598/2005.

- ## Condições Gerais de Exercício

- Atuam em indústrias gráficas ou em setores de embalagens e gráficas de empresas industriais, comerciais ou de serviços, inclusive ensino. Organizam-se em equipe, sobsupervisão

permanente. Trabalham em ambiente fechado, geralmente durante o dia, expostos a **materiais tóxicos,** ruído intenso e altas temperaturas.

-

CBO 5141-Trabalhadores nos serviços de administração de edifícios

Descrição Sumária

Zelam pela segurança das pessoas e do patrimônio de edifícios de apartamentos, edifícios comerciais, igrejas e outros. Atendem e controlam a movimentação de pessoas e veículos no estacionamento; recebem objetos, mercadorias, materiais, equipamentos; conduzem o elevador, realizam pequenos reparos. prestam assistência aos religiosos, ornamentam a igreja e preparam vestes litúrgicas.

Formação e Experiência

O exercício dessas ocupações requer ensino fundamental. Os profissionais aprendem a profissão no próprio emprego. a(s) ocupação(ões) elencada(s) nesta família ocupacional demanda formação profissional para efeitos do cálculo do número de aprendizes a serem contratados pelos estabelecimentos, nos termos do artigo 429da consolidação das leis do trabalho - clt, exceto os casos previstos no art. 10 do decreto 5.598/2005.

Condições Gerais de Exercício

- São trabalhadores assalariados, com carteira assinada. Trabalham em ambiente fecha do e a céu aberto. Organizam-se em equipe e são supervisionados permanentemente ou ocasionalmente, dependendo do serviço executado. Trabalham em horários diurnos, **noturnos e em regime de rodízio de turno.**

-

CBO 5142-Trabalhadores nos serviços de coleta de resíduos, de limpeza e conservação de áreas públicas

Descrição Sumária

Os trabalhadores nos serviços de coleta de resíduos, de limpeza e conservação de áreas públicas coletam resíduos domiciliares, resíduos sólidos de serviços de saúde e resíduos coletados nos serviços de limpeza e conservação de áreas públicas. Preservam as vias públicas, varrendo calçadas, sarjetas e calçadões, acondicionando o lixo para que seja coletado e encaminhado para o aterro sanitário. Conservam as áreas públicas lavando-as, pintando guias, postes, viadutos, muretas e etc.

zelam pela segurança das pessoas sinalizando e isolando áreas de risco e de trabalho. Trabalham com segurança, utilizando equipamento de proteção individual e promovendo a segurança individual e da equipe.

• Formação e Experiência

O acesso às ocupações de faxineiro e limpador de vidros é livre. o exercício das ocupações de coletor de lixo e gari requer quarta série do ensino fundamental e a ocupação de trabalhador de serviços de manutenção de edifícios e logradouros tem como requisito o ensino fundamental completo. O exercício pleno das atividades ocorre após um a dois anos de experiência. a(s) ocupação(ões) elencada(s) nesta família ocupacional demanda formação profissional para efeitos do cálculo do número de aprendizes a serem contratados pelos estabelecimentos, nos termos do artigo429 da consolidação das leis do trabalho - clt, exceto os casos previstos no art. 10do decreto 5.598/2005.

• Condições Gerais de Exercício

• Trabalham em companhias e órgãos de limpeza pública, em condomínios de edifícios, em empresas comerciais e industriais, como assalariados e com carteira assinada; as atividades são realizadas em recintos fechados ou a céu aberto. Trabalham individualmente ou em equipe, com ou sem supervisão permanente. O horário de trabalho é variado, podem do ser diurno, noturno ou em regime de rodízio de turnos. Algumas das atividades podem ser exercidas em grandes alturas, áreas subterrâneas ou em posições desconfortáveis por longos períodos, com exposição a ruído intenso e a poluição dos veículos.

• CBO 5161-Trabalhadores nos serviços de embelezamento e higiene

• Descrição Sumária

Tratam da estética e saúde e aplicam produtos químicos para ondular, alisar ou colorir os cabelos; cuidam da beleza das mãos e pés; realizam depilação e tratamento de pele; fazem maquiagens sociais e para caracterizações (maquiagem artística); realizam massagens estéticas utilizando produtos e aparelhagem; selecionam, preparam e cuidando local e dos materiais de trabalho. podem administrar os negócios.

• Formação e Experiência

As ocupações dessa família requerem para o seu exercício, no mínimo, o ensino fundamental incompleto, curso de qualificação e até um ano de experiência profissional. Principalmente para as funções de esteticistas, observa-se a tendência de aumento de qualificação e escolaridade, com exigência mínima de ensino médio. A(s) ocupação(ões)elencada(s) nesta família ocupacional

demanda formação profissional para efeitos do cálculo do número de aprendizes a serem contratados pelos estabelecimentos, nos termos do artigo 429 da consolidação das leis do trabalho - clt, exceto os casos previstos no art. 10 do decreto 5.598/2005.

- ## Condições Gerais de Exercício

- O trabalho tende a requerer profissionais polivalentes capazes de executar diversas tarefas. Geralmente trabalham em equipe, exceto esteticistas. Trabalham em horários irregulares e em posições desconfortáveis, durante longos períodos. As atividades, geralmente, são executadas sem supervisão, exceto para os que trabalham em grandes redes de institutos de beleza. Há um grande número de profissionais que, por serem proprietários de salão, acumulam atividades burocráticas.

-

- # CBO 3251-Técnico em farmácia e em manipulação farmacêutica

- ## Descrição Sumária

Realizam operações farmacotécnicas, conferem fórmulas, efetuam manutenção de rotina em equipamentos, utensílios de laboratório e rótulos das matérias-primas. Controlam estoques, fazem testes de qualidade de matérias-primas, equipamentos e ambiente. Documentam atividades e procedimentos da manipulação farmacêutica. As atividades são desenvolvidas de acordo com as boas práticas de manipulação, sob supervisão direta do farmacêutico.

- ## Formação e Experiência

Para o exercício dessas ocupações requer-se ensino médio e curso básico de qualifica ção profissional com mais de quatrocentas horas/aula. o pleno desempenho das atividades ocorre entre quatro e cinco anos de experiência profissional. a(s) ocupação(ões)elencada(s) nesta família ocupacional demanda formação profissional para efeitos do cálculo do número de aprendizes a serem contratados pelos estabelecimentos, nos termos do artigo 429 da consolidação das leis do trabalho - clt, exceto os casos previstos no art. 10 do decreto 5.598/2005.

- ## Condições Gerais de Exercício

- Atuam no comércio varejista - farmácias de manipulação - e na indústria de fabricação de produtos químicos como assalariados com carteira assinada. Trabalham em equipe, com supervisão permanente. o trabalho é presencial, realizado em ambiente fechado, durante o dia. Esta família não compreende2234 - farmacêuticos.8103 - supervisores de produção em indústrias de produtos farmacêuticos, cosméticos e afins.8118 - operadores de máquinas e instalações de produtos farmacêuticos, cosméticos e afins.

-

- ## CBO 321110-Técnico agropecuário

- ### Descrição Sumária

Prestam assistência e consultoria técnicas, orientando diretamente produtores sobre produção agropecuária, comercialização e procedimentos de biosseguridade. Executam projetos agropecuários em suas diversas etapas. Planejam atividades agropecuárias, verificando viabilidade econômica, condições e climáticas e infraestrutura. Promovem organização, extensão e capacitação rural. Fiscalizam produção agropecuária. Desenvolvem tecnologias adaptadas à produção agropecuária. Podem disseminar produção orgânica.

- ### Formação e Experiência

O acesso a essas ocupações requer curso técnico agrícola ou em agropecuária (nível médio). O desempenho pleno como técnico titular ocorre com menos de um ano de experiência na área. A atualização dos técnicos é permanente, por meio de cursos de curta duração.

- ### Condições Gerais de Exercício

- Trabalham em empresas públicas e privadas, em atividades de extensão rural e de pesquisas agropecuárias e em órgãos fiscalizadores ou públicos. Trabalham como assalariados, com carteira assinada, ou como autônomos,prestando consultoria técnica. São supervisionados ocasionalmente e as atividades se desenvolvem a céu aberto, nos horários diurnos. Podem trabalhar sob forte pressão e, em algumas das atividades, podem estar sujeitos à exposição de **material tóxico.**

-

- ## CBO 3912 - Técnicos de controle da produção

-

- ### Descrição Sumária

Inspecionam o recebimento e organizam o armazenamento e movimentação de insumos; verificam conformidade de processos; liberam produtos e serviços; trabalham de acordo com normas e procedimentos técnicos, de qualidade e de segurança e demonstram domínio de conhecimentos técnicos específicos da área.

- ### Formação e Experiência

Para o exercício dessas ocupações, requer-se escolaridade mínima de ensino médio, acrescida de cursos básicos de qualificação, que podem variar de duzentas a quatrocentas horas/aula. o desempenho pleno das atividades ocorre após um ou dois anos de experiência. a(s) ocupação(ões)

elencada(s) nesta família ocupacional demanda formação profissional para efeitos do cálculo do número de aprendizes a serem contratados pelos estabelecimentos, nos termos do artigo 429 da consolidação das leis do trabalho - clt, exceto os casos previstos no art. 10 do decreto 5.598/2005.

- ## Condições Gerais de Exercício

- Exercem suas funções em empresas agropecuárias, industriais, comerciais e de serviços, como assalariados, com carteira assinada. Trabalham de forma individual, com supervisão ocasional, em ambientes fechados, em **rodízio de turnos**, nos períodos diurno e **noturno**. Podem permanecer em posições pouco confortáveis durante longos períodos, trabalhar em grandes alturas e podem estar **expostos à ação de materiais tóxicos, radiação**, ruído intenso e altas temperaturas. no desenvolvimento de algumas atividades, podem estar sujeitos a condições especiais, como trabalho confinado.

- # CBO 3171-Técnicos de desenvolvimento de sistemas e aplicações

- ## Descrição Sumária

Desenvolvem sistemas e aplicações, determinando interface gráfica, critérios ergonômicos de navegação, montagem da estrutura de banco de dados e codificação de programas; projetam, implantam e realizam manutenção de sistemas e aplicações; selecionam recursos de trabalho, tais como metodologias de desenvolvimento de sistemas, linguagem de programação e ferramentas de desenvolvimento. planejam etapas e ações de trabalho.

- ## Formação e Experiência

Para o exercício dessas ocupações requer-se ensino técnico de nível médio de informática ou superior incompleto em áreas como ciências exatas, informática, engenharia. A atualização profissional permanente é condição para o seu exercício. o desempenho pleno das atividades do programador de máquinas-ferramentas com comando numérico requer de três a quatro anos de experiência. as demais ocupações, de um a dois anos.

- ## Condições Gerais de Exercício

- Trabalham em atividades de informática e conexas, presentes em todas as atividades econômicas. O programador de máquinas-ferramentas com controle numérico se faz presente na indústria. O programador de sistema de informação e o programador de maquinas ferramentas com controle numérico são, predominantemente, empregados com carteira assinada, ao passo que o programador de multimídia trabalha também como autônomo. As atividades são realizadas no horário diurno, exceto o programador de sistemas de informação, que realiza suas atividades no horário noturno, e o programador de internet, que trabalha em horários irregulares. todas as atividades se desenvolvem em ambiente fechado. Trabalham individualmente e com supervisão

ocasional, exceto o programador de internet, o programador de multimídia e o programador de sistemas de informação, que podem, eventualmente, trabalhar em equipe. Em algumas ocupações, é possível o trabalho a distância. No exercício das atividades, podem permanecer em posições.

-

- ## CBO 3224-Técnicos de odontologia (Técnicos em equipamentos médicos e odontológicos)

- ### Descrição Sumária

Planejam o trabalho técnico-odontológico em consultórios, clínicas, laboratórios deprótese e em órgãos públicos de saúde. previnem doença bucal participando de pro gramas de promoção à saúde, projetos educativos e de orientação de higiene bucal.confeccionam e reparam próteses dentárias humanas, animais e artísticas. Executam procedimentos odontológicos sob supervisão do cirurgião dentista. Administram pessoais e recursos financeiros e materiais. Mobilizam capacidades de comunicação em palestras, orientações e discussões técnicas. As atividades são exercidas conforme normas e procedimentos técnicos e de biossegurança.

- ### Formação e Experiência

O acesso a essas ocupações requer formação profissional técnica em nível médio específica: técnico em laboratório de prótese dentária e técnico em saúde bucal e registro no conselho regional de odontologia (cro). os cursos são oferecidos por instituições de formação profissional e escolas técnicas. a formação profissional dos técnicos oferece, a depender do período que o aluno cursar, a alternativa de atuar como auxiliar em saúde bucal e/ou auxiliar de prótese dentária. o exercício dessas ocupações também é regulamentado pelo cro. a(s) ocupação(ões) elencada(s) nes ta família ocupacional demanda formação profissional para efeitos do cálculo do número de aprendizes a serem contratados pelos estabelecimentos, nos termos do artigo 429 da consolidação das leis do trabalho - clt, exceto os casos previstos no art. 10 do decreto 5. 598/2005.

- ### Condições Gerais de Exercício

- Os técnicos em prótese dentária atuam em laboratórios privados. Desenvolvem o trabalho individualmente ou em equipe, com auxílio de auxiliares de próteses dentárias. Trabalham em conjunto com o cirurgião dentista para restabelecer a capacidade mastigatória e estética (dentária ou facial) por meio de próteses. Os técnicos em saúde bucal (tsb) atuam em clínicas privadas e, majoritariamente, nos serviços odontológicos municipais, estaduais e federais, sob supervisão de cirurgiões dentistas, em horários irregulares. Orientam a população e os pacientes sobre a prevenção e tratamento das doenças bucais. Os auxiliares em saúde bucal exercem atividades de apoio ao cirurgião dentista. Trabalham em locais fechados, podem permanecer em posições desconfortáveis, durante longos períodos. Podem estar sujeitos a exposições de **fotopolimerizadoras, material tóxico, radiação** e ruídos, bem como à pressão para cumprimento de agenda de trabalho.

-

- **CBO 322205 - Técnico de enfermagem**
- **Descrição Sumária:**

Desempenham atividades técnicas de enfermagem em empresas públicas e privadas como: hospitais, clínicas e outros estabelecimentos de assistência médica, embarcações e domicílios; atuam em cirurgia, terapia, puericultura, pediatria, psiquiatria, obstetrícia, saúde ocupacional e outras áreas. Prestam assistência ao paciente zelando pelo seu conforto e bem-estar, administram medicamentos e desempenham tarefas de instrumentação cirúrgica, posicionando de forma adequada o paciente e o instrumental. Organizam ambiente de trabalho e dão continuidade aos plantões. Trabalham em conformidade às boas práticas, normas e procedimentos de biossegurança. Realizam registros e elaboram relatórios técnicos. Desempenham atividades e realizam ações para promoção da saúde da família.

- **Formação e Experiência:**

O ingresso nas ocupações técnicas requer certificação de competências ou curso técnico em enfermagem (nível médio). Para os auxiliares de enfermagem requerem ensino fundamental e cursos de qualificação profissional com o mínimo de quatrocentas horas/ aula, podendo chegar a mil e quinhentas. A possibilidade de continuar a qualificação dependerá da conclusão do ensino médio. Atualmente, há cursos técnicos em enfermagem, organizados modularmente, com saídas intermediárias para qualificação de auxiliares de enfermagem. O requisito de entrada desses cursos é o ensino médio completo, tendo como filosofia a educação continuada, que possibilita ao auxiliar atingir o nível técnico, ao completar novos módulos de formação profissionalizante. A(s) ocupação(ões) elencada(s) nesta família ocupacional demanda formação profissional para efeitos do cálculo do número de aprendizes a serem contratados pelos estabelecimentos, nos termos do artigo 429 da Consolidação das Leis do Trabalho - CLT, exceto os casos previstos no art. 10 do Decreto 5. 598/2005.

- **Condições Gerais de Exercício:**

Trabalham em hospitais, clínicas, serviços sociais, ou ainda em domicílios. São assalariados, com carteira assinada, ou trabalham por conta própria, prestando serviços temporários em clínicas ou em residências. Organizam-se em equipe, atuando com supervisão permanente de enfermeiro ou outro membro de equipe de saúde, de nível superior. Trabalham em ambientes fechados e com revezamentos de turnos, ou confinados em embarcação, no caso do auxiliar de saúde (navegação marítima). Exceção feita aos profissionais que atuam na saúde da família, que de acordo com portaria específica, cumprem jornada de oito horas diárias. É comum trabalharem sob pressão, levando à situação de estresse. Em algumas atividades, podem ser expostos à contaminação biológica, material tóxico e à radiação.

-

- **CBO 324205 - Técnico em patologia clínica**
- **Descrição Sumária:**

Coletam, recebem e distribuem material biológico de pacientes. Preparam amostras do material biológico e realizam exames conforme protocolo. Operam equipamentos analíticos e de suporte. Executam, checam, calibram e fazem manutenção corretiva dos equipamentos. Administram e

organizam o local de trabalho. Trabalham conforme normas e procedimentos técnicos de boas práticas, qualidade e biossegurança. Mobilizam capacidades de comunicação oral e escrita para efetuar registros, dialogar com a equipe de trabalho e orientar os pacientes quanto à coleta do material biológico.

- **Formação e Experiência:**

Para a ocupação de técnico requer-se curso técnico em patologia clínica, em nível médio, oferecido por instituições de formação profissional e escolas técnicas. Para o auxiliar técnico em patologia clínica, o requisito mínimo é ensino fundamental completo, podendo ser exercida por aqueles que cumpriram parcialmente a habilitação técnica. O pleno desempenho das atividades requer experiência inferior a um ano. A(s) ocupação(ões) elencada(s) nesta família ocupacional demanda formação profissional para efeitos do cálculo do número de aprendizes a serem contratados pelos estabelecimentos, nos termos do artigo 429 da Consolidação das Leis do Trabalho - CLT, exceto os casos previstos no art. 10 do Decreto 5.598/2005.

- **Condições Gerais de Exercício:**

Trabalham em laboratórios clínicos, em hospitais e em serviços de saúde pública. São empregados assalariados, com carteira assinada, que trabalham em ambientes fechados, por rodízio de turnos. Via de regra, trabalham individualmente com supervisão de profissionais de nível superior, tais como bioquímicos. Podem permanecer em posições desconfortáveis, por longos períodos. Em algumas das atividades exercidas sofrem exposição a material tóxico, radiação, altas temperaturas e risco biológico.

- **CBO 371110 - Técnico em biblioteconomia**
- **Descrição Sumária:**

Atuam no tratamento, recuperação e disseminação da informação e executam atividades especializadas e administrativas relacionadas à rotina de unidades ou centros de documentação ou informação, quer no atendimento ao usuário, quer na administração do acervo, ou na manutenção de bancos de dados. Participam da gestão administrativa, elaboração e realização de projetos de extensão cultural. Colaboram no controle e na conservação de equipamentos. Participam de treinamentos e programas de atualização.

- **Formação e Experiência:**

Para o exercício das ocupações requer-se formação técnica em biblioteconomia em nível médio e entre quatro e cinco anos de experiência para o exercício pleno das atividades; os auxiliares de biblioteca são técnicos de nível médio que estão no início de carreira, cujo exercício não requer experiência profissional anterior. Os profissionais sem formação técnica profissionalizante devem ser classificados como 4151 - auxiliares de serviços de documentação, informação e pesquisa.

- **Condições Gerais de Exercício:**

Trabalham em bibliotecas, centros de documentação, arquivos, por exemplo, em escolas de ensino fundamental, médio, superior e profissional, associações profissionais, empresas, órgãos de administração pública direta e indireta, institutos de pesquisa e estatística, organizações não governamentais, etc. Seu vínculo de trabalho predominante é como empregado com carteira e seu trabalho se dá, em geral, em grupos com supervisão ocasional ou permanente. Em algumas atividades, alguns profissionais podem trabalhar em condições especiais, sujeitos aos efeitos de esforços repetitivos e de micro-organismos.

-
-

- ## CBO 3201-Técnicos em biologia

- ### Descrição Sumária

Manejam e cuidam da saúde de animais de biotério, tais como: ratos, camundongos e hamsters; auxiliam em experimentação animal, manipulando produtos químicos, coletando tecidos, transplantando pele, confeccionando lâminas, congelando e transferindo embriões; preparam o ambiente e os materiais aplicados ao bioterismo; monitora mas condições ambientais e físicas do biotério; descartam material biológico; operammáquinas e equipamentos. As atividades são desempenhadas segundo boas práticas, normas e procedimentos técnicos e de biossegurança.

- ### Formação e Experiência

O exercício dessas ocupações requer curso técnico em biologia (nível médio) ou áreas afins.

- ### Condições Gerais de Exercício

- O trabalho é exercido em ambientes fechados, em horário diurno, ou por revezamento de turno. Os profissionais atuam, majoritariamente, na condição de assalariados, com carteira assinada. trabalham sob supervisão ocasional, organizados em equipe multidisciplinar, em centros de pesquisa e desenvolvimento, universidades e na área de saúde. em algumas das atividades que exercem são expostos a ruídos, radiação, altas temperaturas, material tóxico e riscos biológicos e alergênicos.

-

- ## CBO 3134-Técnicos em calibração e instrumentação

- ### Descrição Sumária

Analisam tecnicamente a aquisição de produtos e serviços de medição e de controle. Gerenciam documentação técnica e sistemas de confiabilidade; podem coordenar equipes de trabalho; fazem medição. Calibram padrões, equipamentos, sistemas e instrumentos de medição e de controle. Executam, avaliam e realizam manutenção preventiva e ou corretiva de equipamentos e

instrumentos de medição e de controle. Desenvolvem, testam, calibram, operam e reparam instrumentos, aparelhos e equipamentos de medição e controles elétricos, mecânicos, eletromecânicos, eletro hidráulicos e eletrônicos.

- **Formação e Experiência**

O acesso ao trabalho dessas ocupações ocorre por meio de curso técnico de nível médio nas áreas de instrumentação e calibração. os técnicos de instrumentação e calibração executam tarefas semelhantes. ambos atuam no processo (instrumentação)e em laboratórios executando calibrações. Em empresas prestadoras de serviços, os técnicos em instrumentação acompanham o processo produtivo e encaminham os serviços de calibração para serem executados pelos técnicos em calibração no laboratório a atuação desses trabalhadores como titulares na área ocorre depois de um a dois anos de experiência.

- **Condições Gerais de Exercício**

- Atuam em indústrias automobilísticas, de alimentos, celulose e papel, siderurgia, química, refino e transporte de petróleo, farmacêutica, têxtil, geração de energia e saneamento básico, petroquímica, fertilizantes, cimento, borracha e vidro. São empregados assalariados, com carteira assinada que se organizam em equipes, sob supervisão ocasional. Trabalham em ambiente fechado, em grandes alturas e, algumas atividades exercidas podem estar sujeitas à exposição de material tóxico. Consulte3132 - técnicos em eletrônica.
-

- **CBO 3742-Técnicos em cenografia**

- **Descrição Sumária**

Constroem cenários, adereços e mobiliários, a partir de análise de projeto cenográfico e pesquisa de objetos e materiais; executam técnicas afins, como trabalhos de carpintaria, serralheria, costura, pintura, modelagem e escultura; montam e adaptam peças decenários e efeitos especiais ; operam maquinaria, como varas elétricas e cenográficas, cortinas, gruas, carrinhos sobre trilhos e mecanismos de efeitos especiais; supervisionam atividades relacionadas ao planejamento, orçamento e contratação de serviços e orientam equipes de trabalho.

- **Formação e Experiência**

Para o exercício profissional dos maquinistas de cinema e vídeo e de teatro e espetáculos requer-se ensino fundamental completo, seguido de curso de qualificação de duzentas horas/aula e um ano de experiência para o exercício pleno das atividades do cenotécnico requer-se curso técnico profissionalizante de nível médio. O desempenho pleno das atividades ocorre após cinco anos de experiência. a(s) ocupação(ões)elencada(s) nesta família ocupacional demanda formação

profissional para efeitos do cálculo do número de aprendizes a serem contratados pelos estabelecimentos, nos termos do artigo 429 da consolidação das leis do trabalho - clt, exceto os casos previstos no art. 10 do decreto 5.598/2005.

- ## Condições Gerais de Exercício

- Trabalham em atividades artísticas e culturais, por conta própria. Atuam em equipe, com supervisão ocasional do contratante, em ambientes fechados e em horários irregulares. Em algumas atividades podem trabalhar em grandes alturas, confinados, sujeitos a ruído intenso, a permanecer em posições desconfortáveis durante longos períodos, bem como estar expostos a riscos de acidentes e a materiais tóxicos.

-

- # CBO 3121-Técnicos em construção civil (edificações)

- ## Descrição Sumária

Realizam levantamentos topográficos e planialtimétricos. Desenvolvem e legalizam projetos de edificações sob supervisão de um engenheiro civil; planejam a execução, orçam e providenciam suprimentos e supervisionam a execução de obras e serviços. Treinam mão-de-obra e realizam o controle tecnológico de materiais e do solo.

- ## Formação e Experiência

Para o exercício dessas ocupações requer-se curso técnico em edificações, técnico em construção civil de várias modalidades, em nível médio, oferecidos pelas instituições de formação profissional e escolas técnicas, com registro no CREA. O desempenho pleno das atividades ocorre com menos de um ano de experiência na área.

- ## Condições Gerais de Exercício

- Trabalham na construção civil e indústrias de materiais para construção. Podem, também, trabalhar em laboratórios de pesquisa e desenvolvimento, planejamento, orçamento, projetos, gerenciamento, controle e execução de obras. Trabalham em equipe, sob supervisão ocasional, com carteira assinada ou por conta própria. Atuam em ambientes fechados ou abertos, por rodízio de turnos. Frequentemente estão sujeitos ao trabalho em grandes alturas, expostos a ruídos, material tóxico e condições variáveis de temperatura.

-

- # CBO 3122-Técnicos em construção civil (obras de infraestrutura)

- **Descrição Sumária**

Planejam a execução do trabalho e supervisionam equipes de trabalhadores de construção de obras de infraestrutura. Auxiliam engenheiros no desenvolvimento de projetos, no levantamento e tabulação de dados e na vistoria técnica. Estruturam o serviço de coleta de resíduos sólidos das obras, controlando os procedimentos de preservação do meio ambiente. Realizam trabalhos de laboratório, vendas e compras de materiais e equipamentos. Padronizam procedimentos técnicos.

- **Formação e Experiência**

O acesso a essas ocupações requer curso técnico de nível médio em construção civil edificações, ou cursos afins, e registro no CREA. Esses profissionais estão aptos a atuarem laboratórios, centros de pesquisa e desenvolvimento, departamentos de compra e venda de terrenos, e na fiscalização e execução de obras, realizando levantamentos topográficos e elaborando projetos. Atingem o pleno exercício da profissão depois deum a dois anos de prática profissional na área.

- **Condições Gerais de Exercício**

- Trabalham em empresas de construção, reciclagem, captação, purificação e distribuição de água, coleta de lixo e águas residuais, esgoto doméstico e industrial e outras atividades empresariais. Podem trabalhar em locais fechados ou a céu aberto. Esses profissionais são empregados assalariados, com carteira assinada, que se organizam em equipes, sob supervisão ocasional. Estão sujeitos ao trabalho em locais subterrâneos ou confinados e, muitas vezes, ficam expostos a grandes alturas, ruídos e material tóxico.

-

- **CBO 3511-05-Técnico de contabilidade**

- **Descrição Sumária**

Realizam atividades inerentes à contabilidade em empresas, órgãos governamentais e outras instituições públicas e privadas. Para tanto, constituem e regularizam empresa, identificam documentos e informações, atendem à fiscalização e procedem consultoria empresarial. Executam a contabilidade geral, operacionalizam a contabilidade de custos e efetuam contabilidade gerencial. Administram o departamento pessoal e realizam controle patrimonial.

- **Formação e Experiência**

O exercício dessas ocupações requer curso técnico em contabilidade (nível médio). o exercício pleno das atividades ocorre após quatro anos de experiência. a(s)ocupação(ões) elencada(s) nesta família ocupacional demanda formação profissional para efeitos do cálculo do número de

aprendizes a serem contratados pelos estabelecimentos, nos termos do artigo 429 da consolidação das leis do trabalho - clt, exceto os casos previstos no art. 10 do decreto 5.598/2005.

- ## Condições Gerais de Exercício

- Trabalham em escritórios de contabilidade, em departamentos de contabilidade de empresas agrícolas, comerciais, industriais e de serviços e em órgãos governamentais, como estatutários, empregados assalariados ou como autônomos (consultor contábil).O trabalho é presencial ou a distância; pode ser realizado de forma individual sem supervisão, ou em equipe, sob supervisão. Os profissionais trabalham em ambiente fechado, no período diurno. O chefe de contabilidade e o técnico de contabilidade permanecem, durante longos períodos, em posições desconfortáveis e trabalham sob pressão, o que pode levá-los à situação de estresse constante.

-

- # CBO 3115-Técnicos em controle ambiental, utilidades e tratamento de efluentes

- ## Descrição Sumária

Auxiliam profissionais de nível superior na implementação de projetos, gestão ambiental e coordenação de equipes de trabalho; operam máquinas, equipamentos e instrumentos. Coordenam processos de controle ambiental, utilidades, tratamento de efluentes e levantamentos meteorológicos. Realizam análises físico-químicas e micro biológicas dos efluentes. Monitoram a segurança no trabalho.

- ## Formação e Experiência

Essas ocupações requerem formação técnica de nível médio completa nas áreas do meio ambiente, saneamento e afins. O pleno exercício das atividades requer de um a dois anos de experiência. Geralmente, trabalham sob supervisão de profissionais de nível superior.

- ## Condições Gerais de Exercício

- Atuam na preservação da qualidade ambiental. Trabalham em equipe, em laboratórios em atividades de campo, vinculados à administração pública, indústrias, empresas de consultoria, estações meteorológicas e de tratamento. Trabalham em ambientes fechados, a céu aberto ou em veículos nos horários diurnos e noturnos. Muitas vezes, trabalham sob pressão, em posições desconfortáveis ou expostos a ruídos, **material tóxico**, radiação, altas temperaturas, frio intenso e umidade.consulte3111 - técnicos químicos.

- # CBO 3131-Técnicos em eletricidade e eletrotécnica

- **Descrição Sumária**

Planejam atividades do trabalho, elaboram estudos e projetos, participam no desenvolvimento de processos, realizam projetos, operam sistemas elétricos e executam manutenção. Atuam na área comercial, gerenciam e treinam pessoas, asseguram a qualidade de produtos e serviços e aplicam normas e procedimentos de segurança no trabalho.

- **Formação e Experiência**

Essas ocupações são exercidas por trabalhadores com escolaridade de ensino médio completo, acrescida de curso de formação profissional em nível médio - curso técnico em eletricidade, eletrotécnica ou área correlata. o desempenho pleno das funções ocorre após um ano de experiência profissional.

- **Condições Gerais de Exercício**

- São contratados na condição de trabalhadores assalariados, com carteira assinada. Trabalham em equipe, sob supervisão ocasional, em ambientes fechados e, também, a céu aberto. Atuam de forma presencial, em períodos diurnos e noturnos e em rodízio de turnos. No desenvolvimento de algumas atividades, podem trabalhar em posições desconfortáveis durante longos períodos e atuar sob pressão, levando-os à condição de estresse. Os profissionais das ocupações eletrotécnico, eletrotécnico (produção de energia) e técnico eletricista podem executar algumas atividades em grandes alturas e, também, na condição de trabalho subterrâneo e confinado. Os profissionais das ocupações técnicos de manutenção elétrica de máquina e técnico de manutenção elétrica podem estar expostos à ação de materiais tóxicos.

-

- **CBO 313215 - Técnico eletrônico**
- **Descrição Sumária:**

Consertam e instalam aparelhos eletrônicos, desenvolvem dispositivos de circuitos eletrônicos, fazem manutenções corretivas, preventivas e preditivas, sugerem mudanças no processo de produção, criam e implementam dispositivos de automação. Treinam, orientam e avaliam o desempenho de operadores. Estabelecem comunicação oral e escrita para agilizar o trabalho, redigem documentação técnica e organizam o local de trabalho. Podem ser supervisionados por engenheiros eletrônicos. Consertam e instalam aparelhos eletrônicos, desenvolvem dispositivos de circuitos eletrônicos, fazem manutenções corretivas, preventivas e preditivas, sugerem mudanças no processo de produção, criam e implementam dispositivos de automação. Treinam, orientam e avaliam o desempenho de operadores. Estabelecem comunicação oral e escrita para agilizar o trabalho, redigem documentação técnica e organizam o local de trabalho. Podem ser supervisionados por engenheiros eletrônicos.

- **Formação e Experiência:**

Para ingressar nessas ocupações é necessário que os profissionais tenham registro no crea e formação técnica de nível médio em eletrônica ou em áreas afins, como mecatrônica, eletroeletrônica, eletromecânica ou técnico em manutenção eletrônica e manutenção de equipamentos de informática. É desejável possuir curso de especialização complementar ou de atualização com duração superior a quatrocentas horas/aula. A atuação como técnico titular ocorre normalmente com três a cinco anos de experiência, dependendo da área de atuação.

- **Condições Gerais de Exercício:**

A maioria desses profissionais trabalha com registro em carteira, porém alguns podem atuar como autônomos. Atuam nas indústrias de fabricação de máquinas e equipamentos, componentes elétricos, eletrônicos, microcomputadores e equipamentos de comunicações, laboratórios de controle de qualidade, manutenção e pesquisa e nas empresas de assistência técnico-comercial. Geralmente se organizam em equipe, sob supervisão ocasional de profissionais de nível superior. Trabalham em locais fechados em horários irregulares ou por rodízio de turnos. Em algumas das atividades exercidas são expostos a ruídos, altas temperaturas, radiação e material tóxico.

- **CBO 915305 - Técnico em manutenção de equipamentos e instrumentos médico-hospitalares**
- **Descrição Sumária:**

Realizam manutenção, testes e ensaios e instalam equipamentos e instrumentos médico- odonto-hospitalares. Elaboram documentação técnica. Treinam equipe técnica e usuários e prestam atendimento a clientes. Trabalham em conformidade com normas técnicas, de qualidade, de segurança e higiene.

- **Formação e Experiência:**

O exercício profissional requer formação técnica de nível médio e noções de funcionamento dos órgãos do corpo humano. O pleno exercício das atividades ocorre após três ou quatro anos de experiência. A(s) ocupação(ões) elencada(s) nesta família ocupacional demanda formação profissional para efeitos do cálculo do número de aprendizes a serem contratados pelos estabelecimentos nos termos do artigo 429 da Consolidação das Leis do Trabalho - CLT, exceto os casos previstos no art. 10 do Decreto 5.598/2005.

- **Condições Gerais de Exercício:**

Atuam em empresas de serviços de saúde e de fabricação de equipamentos e instrumentos médico-hospitalares. Trabalham como assalariados, com registro em carteira e se organizam em equipe no trabalho, sob supervisão ocasional de engenheiros. O local de trabalho é fechado e o horário, diurno. Em algumas atividades podem estar sujeitos à exposição de radiação e contaminação e à pressão de trabalho que pode levar ao estresse.

- **CBO 313505 - Técnico em fotônica**
- **Descrição Sumária:**

Aplicam a tecnologia da fotônica utilizando princípios de física, química e matemática e sistemas básicos de óptica, identificando fontes de luz, detectores e ou sensores de luz; projetam sistemas em

fotônica; desenvolvem protótipos; instalam produtos ou sistemas fotônicos; realizam medições e manutenções em equipamentos fotônicos. Trabalham em conformidade com normas e padrões técnicos, de segurança, de gestão da qualidade e do meio ambiente. Documentam atividades e procedimentos.

- **Formação e Experiência:**

Os trabalhadores dessa família ocupacional são técnicos profissionalizantes de várias áreas que se especializam em fotônica. Na fotônica, o conhecimento da aplicação é crítico. Portanto, ela pode ser uma área de especialização cujo exercício pleno das atividades demanda três a quatro anos de experiência.

- **Condições Gerais de Exercício:**

Esses trabalhadores atuam em áreas ligadas à fabricação de material eletrônico e de aparelhos e equipamentos de comunicação, campos especializados, como defesa, segurança pública, indústria aeroespacial, comunicações, medicina, meio ambiente, energia, transportes, manufaturas com fotônica (e.g. Equipamentos a laser, fibras óticas), testes e análises, computadores. São empregados com carteira, trabalham de forma individual, com supervisão ocasional, em ambiente fechado e em horário diurno. Eventualmente, são expostos a materiais tóxicos e à luz.

- **CBO 313505 - Técnico em fotônica**
- **Descrição Sumária:**

Aplicam a tecnologia da fotônica utilizando princípios de física, química e matemática e sistemas básicos de óptica, identificando fontes de luz, detectores e ou sensores de luz; projetam sistemas em fotônica; desenvolvem protótipos; instalam produtos ou sistemas fotônicos; realizam medições e manutenções em equipamentos fotônicos. Trabalham em conformidade com normas e padrões técnicos, de segurança, de gestão da qualidade e do meio ambiente. Documentam atividades e procedimentos.

- **Formação e Experiência:**

Os trabalhadores dessa família ocupacional são técnicos profissionalizantes de várias áreas que se especializam em fotônica. Na fotônica, o conhecimento da aplicação é crítico. Portanto, ela pode ser uma área de especialização cujo exercício pleno das atividades demanda três a quatro anos de experiência.

- **Condições Gerais de Exercício:**

Esses trabalhadores atuam em áreas ligadas à fabricação de material eletrônico e de aparelhos e equipamentos de comunicação, campos especializados, como defesa, segurança pública, indústria aeroespacial, comunicações, medicina, meio ambiente, energia, transportes, manufaturas com fotônica (e.g. Equipamentos a laser, fibras óticas), testes e análises, computadores. São empregados com carteira, trabalham de forma individual, com supervisão ocasional, em ambiente fechado e em horário diurno. Eventualmente, são expostos a materiais tóxicos e à luz.

- ## CBO 3123-Técnicos em geomática

- ### Descrição Sumária

Executam levantamentos geodésicos e topohidrográficos, por meio de levantamentos altimétricos e planimétricos; implantam, no campo, pontos de projeto, locando obras de sistemas de transporte, obras civis, industriais, rurais e delimitando glebas; planejam trabalhos em geomática; analisam documentos e informações cartográficas, interpretando fotos terrestres, fotos aéreas, imagens orbitais, cartas, mapas, plantas, identificando acidentes geométricos e pontos de apoio para georreferenciamento e amarração, coletando dados geométricos. Efetuam cálculos e desenhos e elaboram documentos cartográficos, definindo escalas e cálculos cartográficos, efetuando aero triangulação, restituindo fotografias aéreas.

- ### Formação e Experiência

O exercício dessas ocupações requer curso técnico de nível médio em geomática ou correlatas, como técnico em geodésia e cartografia, técnico em agrimensura, técnico em hidrografia, técnico em topografia, oferecidos por escolas técnicas e instituições de formação profissional. Para as ocupações de técnico em agrimensura, em hidro grafia e topógrafo, o desempenho pleno das atividades ocorre após o período de um a dois anos de experiência.

- ### Condições Gerais de Exercício

- Exercem suas funções na condição de trabalhadores assalariados, com carteira assina da em empresas do ramo de construção, pesquisa e desenvolvimento, administração pública, defesa e seguridade social e empresas de transporte. Atuam em serviços de campo, trabalham, dependendo da ocupação, sob supervisão permanente e ocasional, em ambientes fechados e também a céu aberto, no período diurno. no desempenho de algumas de suas atividades podem permanecer em posições desconfortáveis durante longos períodos, podendo, ainda, trabalhar sob pressão, ocasionando estresse.
-

- ## CBO 3143-Técnicos em mecânica veicular

- ### Descrição Sumária

Realizam ensaios e testes e montam componentes na fabricação e manutenção veicular automobilística, naval e aeronáutica, de acordo com normas de qualidade e de segurança do trabalho. Prestam assessoria a equipes internas e externas.

- ### Formação e Experiência

Para o ingresso nas ocupações requer-se curso de mecânica veicular, em nível médio profissionalizante, ou que estejam cursando o ensino superior na área de engenharia mecânica, naval ou aeronáutica. o pleno exercício das atividades como técnico titular ocorre com menos de um ano de experiência profissional.

- **Condições Gerais de Exercício**

- Trabalham principalmente em empresas de fabricação e manutenção automotiva, aeronáutica e naval, bem como em empresas de transporte sobre rodas, marítimo e aéreo. São empregados assalariados, com carteira assinada, organizados em times (grupos de trabalho), sob supervisão ocasional. Trabalham por rodízio de turnos em locais abertos, fechados ou em veículos. Eventualmente, no trabalho podem ser submetidos a ruídos e a situações estressantes.

- **CBO 3732-Técnicos em operação de sistemas de televisão e de produtoras de vídeo**

- **Descrição Sumária**

Coordenam atividades de operação de sistemas de televisão e produtoras de vídeo, nas fases de planejamento e execução, gerenciando recursos humanos, financeiros e uso dos equipamentos. produzem eventos externos, manipulam áudio e vídeo; dirigem e

- **Formação e Experiência**

O exercício dessas ocupações requer curso técnico de nível médio na área de rádio e televisão. o desempenho pleno das atividades ocorre com mais de cinco anos de experiência (supervisor técnico), após três ou quatro anos (técnico em operação de equipamento de exibição de tv e técnico em equipamentos de transmissão-recepçãode tv), após um ou dois anos (técnico em operação de equipamento de produção para tv e produtora de vídeo). a complementação da formação técnica ocorre na própria emissora ou empresa, visto que há um estreito relacionamento entre a operação e o tipo de tecnologia dos equipamentos. Nas grandes emissoras há um grau de especialização mais acentuado que nas emissoras menores.

- **Condições Gerais de Exercício**

- Trabalham em emissoras de televisão e produtoras de vídeo, em empresas de edição, impressão e reprodução de gravações ou em atividades recreativas, culturais e desportivas. O horário de trabalho é irregular ou, ainda, em rodízio de turnos. Podem trabalhar internamente, nas emissoras, a céu aberto ou em veículos. Em algumas das atividades permanecem em posições desconfortáveis durante longos períodos. Podem estar expostos a grandes alturas, ruídos, materiais tóxicos e radiação. São trabalhadores assalariados, com carteira assinada, trabalhando individualmente ou em equipe, sob supervisão ocasional. consulte3721 - captadores de imagens em movimento.

•

• CBO 3252-Técnicos em produção, conservação e de qualidade de alimentos

• Descrição Sumária

Os técnicos em produção, conservação e de qualidade de alimentos controlam a qualidade dos alimentos nas etapas de produção, supervisionando processos produtivos e de distribuição, verificando condições de ambiente, equipamento e produtos (in natura e preparados). Podem participar de pesquisa para melhoria, adequação e desenvolvimento de produtos e promover a venda de insumos, produtos e equipamentos. Os técnicos em alimentos atuam prioritariamente na indústria alimentícia. os técnicos em nutrição e dietética trabalham sob supervisão de nutricionista, atuando, prioritariamente, em unidades de alimentação e nutrição (coletividade sadia) e unidades de nutrição e dietética (coletividade preferencialmente enfermas) e saúde coletiva.

• Formação e Experiência

Para o exercício profissional requer-se curso técnico em alimentos (nível médio), cursos afins ou especializados como, por exemplo, em laticínios, em leite e derivados, em açúcar e álcool, oferecidos por instituições de formação profissional e escolas técnicas, além do registro profissional no conselho regional competente. O exercício pleno das atividades é obtido durante o primeiro ano de experiência, após estágio. a(s)ocupação(ões) elencada(s) nesta família ocupacional demanda formação profissional para efeitos do cálculo do número de aprendizes a serem contratados pelos estabelecimentos, nos termos do artigo 429 da consolidação das leis do trabalho - clt, exceto os casos previstos no art. 10 do decreto 5.598/2005.

• Condições Gerais de Exercício

• Atuam em indústrias alimentícias diversas, em centros de pesquisa, laboratórios de avaliação da qualidade, vigilância sanitária e empresas de comercialização de alimentos. São empregados assalariados, com carteira assinada, organizados em equipe, sobs upervisão ocasional. Trabalham em locais fechados, em rodízio de turnos. Em algumas das atividades exercidas, trabalham em posições desconfortáveis por longos períodos, expostos a altas temperaturas, odores intensos, ruídos e **material tóxico.**

•

• CBO 3225 - Técnicos em próteses ortopédicas

• Descrição Sumária

Interpretam as especificações médicas e efetuam as medidas do paciente para desenvolver, projetar, confeccionar, adaptar e reparar órteses e próteses (o/p), tais como aparelhos para correção ou apoio para pessoas com lesões em qualquer parte do corpo e membros artificiais. avaliam o paciente e a prescrição; planejam, confeccionam e acompanham o funcionamento de órteses e próteses prestando assistência técnica; gerenciam o ateliê. O desenvolvimento do trabalho requer o uso de capacidades de comunicação do profissional junto aos profissionais da área e os pacientes.

- **Formação e Experiência**

O exercício pleno das atividades dessa ocupação requer o ensino médio completo e mais de cinco anos de experiência profissional. a(s) ocupação(ões) elencada(s) nesta família ocupacional demanda formação profissional para efeitos do cálculo do número de aprendizes a serem contratados pelos estabelecimentos, nos termos do artigo 429da consolidação das leis do trabalho - clt, exceto os casos previstos no art. 10 do decreto 5.598/2005.

- **Condições Gerais de Exercício**

- Atuam na área da saúde e serviços sociais. São empregadores, trabalham de forma individual e em equipe com a equipe médica, sem supervisão. Executam suas funções em ambiente fechado e em horário diurno. Permanecem em posições desconfortáveis durante longos períodos e são expostos a materiais tóxicos, ruído intenso, altas temperaturas e ao pó dos materiais.

- **CBO 3516-05-Técnico em segurança do trabalho**

- **Descrição Sumária**

Elaboram, participam da elaboração e implementam política de saúde e segurança no trabalho (sst); realizam auditoria, acompanhamento e avaliação na área; identificam variáveis de controle de doenças, acidentes, qualidade de vida e meio ambiente. Desenvolvem ações educativas na área de saúde e segurança no trabalho; participam de perícias e fiscalizações e integram processos de negociação. participam da adoção de tecnologias e processos de trabalho; gerenciam documentação de sst; investigam, analisam acidentes e recomendam medidas de prevenção e controle.

- **Formação e Experiência**

O exercício dessa ocupação requer formação de nível médio e curso técnico de segurança no trabalho. a(s) ocupação(ões) elencada(s) nesta família ocupacional demanda formação profissional para efeitos do cálculo do número de aprendizes a serem contratados pelos estabelecimentos, nos termos do artigo 429 da consolidação das leis do trabalho - clt, exceto os casos previstos no art. 10 do decreto 5.598/2005.

- **Condições Gerais de Exercício**

- Exercem suas funções em empresas dos mais diversos ramos de atividades. São contratados na condição de trabalhadores assalariados, com carteira assinada. Em geral, atuam de forma individual, sob supervisão permanente, em ambientes fechados, no período diurno, exercendo o trabalho de forma presencial. Algumas de suas atividades podem ser desenvolvidas sob pressão, levando-os à situação de estresse. Os profissionais podem, ainda, estar expostos à ação de materiais tóxicos, radiação, ruído intenso e altas temperaturas.

-

- **CBO 321210-Técnico florestal**

- **Descrição Sumária**

Supervisionam, execução de atividades florestais, desde a construção de viveiros flores tais e infraestrutura, produção de mudas e colheita florestal até o manejo de florestas nativas e comerciais; inventariam florestas, planejam atividades florestais; elaboram documentos técnicos. administram unidades de conservação e de produção, atuam na preservação e conservação ambiental; fiscalizam e monitoram fauna e flora; ministram treinamentos e podem participar de pesquisas.

- **Formação e Experiência**

O acesso a essas ocupações requer curso técnico florestal ou curso pós-técnico flores tal, de nível médio. Trabalham em equipe multidisciplinar, sob supervisão ocasional de engenheiros florestais e afins. O pleno exercício da atividade é atingido após experiência profissional de um a dois anos na área.

- **Condições Gerais de Exercício**

- Atuam em instituições públicas e privadas ligadas a atividades florestais, empresas de fabricação de produtos de madeira, indústrias de papel e celulose, instituições de pesquisas e desenvolvimento, reservas ecológicas e indústrias de silvicultura e exploração florestal. Técnicas avançadas de mapeamento, possibilitadas pelo sistema de posicionamento global (gps) têm facilitado e agilizado a identificação de eventos a fiscalizar, ampliando o mercado de trabalho. trabalham a céu aberto e em ambiente fechado. São assalariados, com carteira assinada. atuam sob condições favoráveis de trabalho. Em algumas atividades, podem estar sujeitos a ruídos e material tóxico.

-

- **CBO 3142-Técnicos mecânicos (ferramentas)**

- **Descrição Sumária**

Pesquisam o mercado, desenvolvem e realizam manutenção de ferramentas e dispositivos para fabricação mecânica. Providenciam recursos técnicos para a fabricação do produto final para o qual foram desenvolvidas as ferramentas. Planejam a manutenção preventiva e corretiva de equipamentos e ferramentas, bem como propõem melhoria contínua de processos de fabricação, segundo critérios de qualidade e segurança no trabalho. Podem prestar assistência técnica.

- **Formação e Experiência**

Para o acesso ao trabalho nessas ocupações requer-se curso técnico em mecânica. É desejável que o técnico tenha cursado também qualificação profissional básica, com carga horária superior a quatrocentas horas/aula. o desempenho profissional pleno geralmente ocorre após três a quatro anos de experiência em construção e manutenção de ferramentas.

- **Condições Gerais de Exercício**

- Atuam em empresas de fabricação de máquinas, equipamentos e produtos metalúrgicos, nas áreas de desenvolvimento, fabricação e manutenção de ferramentas e dispositivos de fabricação mecânica. São empregados assalariados, com carteira assinada e normalmente trabalham em rodízio de turnos e em grupos de trabalho (times), sob supervisão ocasional. Algumas das atividades exercidas por esses técnicos os expõem a ruídos e ao estresse.

-

- **CBO 3144-Técnicos mecânicos na manutenção de máquinas, sistemas e instrumentos**

- **Descrição Sumária**

Planejam a manutenção de máquinas, sistemas e instrumentos; supervisionam processos de manutenção; mantêm equipamentos, instrumentos, máquinas e sistemas em condições plenas de funcionamento e calibram instrumentos e equipamentos. Elaboram procedimentos técnicos e administrativos; propõem melhorias em máquinas, instrumentos e sistemas; aplicam técnicas de segurança e normas ambientais; prestam assessoria técnica em manutenção e realizam testes e ensaios.

- **Formação e Experiência**

O exercício profissional dessas ocupações requer curso técnico de nível médio, com ênfase em manutenção de máquinas e instrumentação. o pleno exercício das atividades ocorre após o período de um a dois anos de experiência.

- **Condições Gerais de Exercício**

- Trabalham em empresas de extração de petróleo e serviços correlatos; de fabricação de produtos têxteis, metalurgia básica, fabricação de máquinas e equipamentos, fabricação e montagem de veículos automotores, reboques e carrocerias, entre outras. São assalariados, com carteira assinada. atuam em equipe multidisciplinar (mecânica e elétrica), sob supervisão ocasional; em ambientes fechados; de forma presencial; em turnos de trabalho, com disponibilidade para atuar em horários irregulares, para atendimento a situações imprevistas e de emergência. No desenvolvimento de algumas atividades, podem permanecer em posições desconfortáveis durante períodos e, ainda, podem estar expostos a ruído intenso e trabalhar sob pressão, podendo ocasionar estresse.
-

- # CBO 3111-05-Técnico químico

- ## Descrição Sumária

Executam ensaios físico-químicos, participam do desenvolvimento de produtos e processos, da definição ou reestruturação das instalações industriais; supervisionam operação de processos químicos e operações unitárias de laboratório e de produção, operam máquinas e/ou equipamentos e instalações produtivas, em conformidade com as normas de qualidade, de boas práticas de manufatura, de biossegurança e controle do meio ambiente. Interpretam manuais, elaboram documentação técnica rotineira e de registros legais. Podem ministrar programas de ações educativas e prestar assistência técnica. Todas as atividades são desenvolvidas conforme os limites de responsabilidade técnica previstos em lei.

- ## Formação e Experiência

Para o exercício dessas ocupações requer-se formação técnica profissionalizante de nível médio em habilitações como técnico em química, técnico em celulose e papel, técnico químico em curtimento e couro e outros cursos afins, com núcleo formativo em química e registro profissional no conselho competente. a formação generalista é mesclada com um enfoque especialista no qual o técnico se especializa no processo em que atua. Requer-se raciocínio sintético e analítico com competência para intervenções rápidas e apropriadas para o seu dia-a-dia no trabalho, principalmente em situações de risco. A atuação como técnico titular demanda, pelo menos, um ano de experiência na área.

- ## Condições Gerais de Exercício

- Os técnicos químicos são empregados assalariados, com carteira assinada, que trabalham em indústrias químicas, petroquímicas, de açúcar e álcool, fármacos, alimentos, bebidas, papel e celulose, fertilizantes, tintas e vernizes, cosméticos e perfumes, materiais de construção, plásticos, refratários e cerâmicos. O trabalho é realizado em equipe e recebem supervisão ocasional. Algumas das atividades exercidas por estes profissionais podem estar sujeitas a ruídos, poeira, gases, vapores e material tóxico.

-

- ## CBO 2233-Veterinários e zootecnistas

- ### Descrição Sumária

Praticam clínica médica veterinária em todas as suas especialidades; contribuem para o bem-estar animal; podem promover saúde pública e defesa do consumidor; exercem defesa sanitária animal; desenvolvem atividades de pesquisa e extensão; atuam nas produções industrial e tecnológica e no controle de qualidade de produtos. Fomentam produção animal; atuam nas áreas comercial agropecuária, de biotecnologia e de preservação ambiental; elaboram laudos, pareceres e atestados; assessoram a elaboração de legislação pertinente.

- ### Formação e Experiência

Essas ocupações são exercidas por profissionais com escolaridade de ensino superior completo ou com formação em cursos superiores de tecnologia (tecnólogos). O exercício pleno das funções ocorre após o período de um ano de experiência profissional.

- ### Condições Gerais de Exercício

- Os profissionais dessa família ocupacional podem exercer suas funções nos setores cujas atividades referem-se à pecuária e serviços relacionados, de modo geral, atuam na condição de trabalhadores contratados, com carteira assinada, trabalham de forma individual e com supervisão ocasional. Desenvolvem as atividades em ambientes fechados e a céu aberto, em períodos diurnos, porém, com irregularidades de horários. Podem trabalhar em posições desconfortáveis durante longos períodos e, ainda, o trabalho pode ocorrer em situação de pressão provocando estresse constante. Algumas vezes, podem estar sujeitos à ação de materiais tóxicos, radiação, ruído intenso, riscos biológicos e baixas temperaturas.

-

- ## CBO 5173-Vigilantes e guardas de segurança

- ### Descrição Sumária

Vigiam dependências e áreas públicas e privadas com a finalidade de prevenir, controlar e combater delitos como porte ilícito de armas e munições e outras irregularidades; zelam pela segurança das pessoas, do patrimônio e pelo cumprimento das leis e regulamentos; recepcionam e controlam a movimentação de pessoas em áreas de acesso livre e restrito; fiscalizam pessoas, cargas e patrimônio; escoltam pessoas e mercadorias. Controlam objetos e cargas; vigiam parques e reservas florestais, combatendo inclusive focos de incêndio; vigiam presos. Comunicam-se via rádio ou telefone e prestam informações ao público e aos órgãos competentes.

- ## Formação e Experiência

O exercício das ocupações requer ensino médio completo, exceto agente de proteção de aeroporto e vigilante, que têm como requisito o ensino fundamental. Todas as ocupações requerem formação profissionalizante básica de duzentas a quatrocentas horas. Os vigilantes passam por treinamento obrigatório em escolas especializadas em segurança, onde aprendem a utilizar armas de fogo. a(s) ocupação(ões) elencada(s) nesta família ocupacional demanda formação profissional para efeitos do cálculo do número de aprendizes a serem contratados pelos estabelecimentos, nos termos do artigo 429 da consolidação das leis do trabalho - clt, exceto os casos previstos no art. 10 do decreto 5.598/2005.

- ## Condições Gerais de Exercício

- São, em geral, assalariados, com carteira assinada, que atuam em estabelecimentos diversos de defesa e segurança e de transporte terrestre, aéreo ou aquaviário. Podem trabalhar em equipe ou individualmente, com supervisão permanente, em horários diurnos, noturnos, em rodízio de turnos ou escala. Trabalham em grandes alturas, confinados ou em locais subterrâneos. estão sujeitos a risco de morte e trabalham sob pressão constante, expostos a ruídos, radiação, material tóxico, poeira, fumaça e baixas temperaturas.

Análise de Correspondência Múltipla

Tabela S1. Dimensões, autovalores e percentual de explicação da variância.

Dimensões	Autovalores	% variância	% acumulado variância
Dim.1	0,578739	79,90	79,90
Dim.2	0,105336	14,54	94,44
Dim.3	0,021879	3,02	97,46
Dim.4	0,017289	2,39	99,85
Dim.5	0,001062	0,15	100

Tabela S2. Coordenadas, medida de correlação e contribuição relativa das categorias das variáveis.

Variáveis	Dimensão 1			Dimensão 2		
	Coordenada	Cos^2	Contribuição (%)	Coordenada	Cos^2	Contribuição (%)
OC:C	0,293	0,705	10,00	-0,104	0,088	6,8
OC:NC	-0,179	0,705	6,10	0,063	0,088	4,2
O:CNRT	-0,077	0,465	1,10	0,024	0,044	0,6
O:CRT	0,117	0,465	1,70	-0,036	0,044	0,9
TN:E	0,364	0,656	15,40	0,077	0,029	3,8
TN:NE	-0,223	0,656	9,40	-0,047	0,029	2,3
ET:ADT	0,189	0,58	2,60	0,093	0,142	3,5
ET:BDT	-0,261	0,588	3,60	0,004	0	0
ET:TA	-0,141	0,458	1,20	0,087	0,173	2,5
ET:TP	0,07	0,173	0,60	-0,104	0,38	7
SEX:F	-0,112	0,59	1,80	0,061	0,177	2,9
SEX:M	0,097	0,59	1,50	-0,053	0,177	2,5
JT:<40H	-0,018	0,074	0,10	-0,031	0,229	1,3
JT:>40H	0,056	0,074	0,20	0,099	0,229	4
E:F	0,242	0,349	4,80	-0,229	0,314	23,5
E:M	0,152	0,502	2,40	0,12	0,314	8,2
E:S	-0,292	0,795	10,30	0,052	0,025	1,8
TU:D	-0,262	0,66	11,50	-0,05	0,024	2,3
TU:P	0,322	0,66	14,20	0,062	0,024	2,9
I:35-49	0,105	0,175	1,10	0,15	0,354	12,6
I:50-72	-0,053	0,175	0,60	-0,076	0,354	6,4
