# Supplementary material for: Occupational, socioeconomic factors and cancer mortality in participants of the Longitudinal Study of Adult Health (ELSA-Brazil): a multiple correspondence analysis
Source: Rev Bras Epidemiol. 2025 May 2;28:e250022. doi: 10.1590/1980-549720250022 (PMC12054984; doi:10.1590/1980-549720250022)
Supplement: Translation

# Translation: Fatores ocupacionais e socioeconômicos e mortalidade por câncer em participantes do Estudo Longitudinal de Saúde do Adulto (ELSA-Brasil): uma análise de correspondência múltipla

**DOI:** 10.1590/1980-549720250022.2

**Authors:** Débora Cristina de Almeida Mariano Bernardino, Ubirani Barros Otero, Isiyara Taverna Pimenta, Luana Giatti, Rosane Harter Griep, Maria de Jesus Mendes da Fonseca

**Affiliations:** I Fundação Oswaldo Cruz, Escola Nacional de Saúde Pública – Rio de Janeiro (RJ), Brasil.; II Instituto Nacional de Câncer – Rio de Janeiro (RJ), Brasil.; III Universidade Federal de Minas Gerais – Belo Horizonte (MG), Brasil.; IV Fundação Oswaldo Cruz, Laboratório de Educação em Saúde e Meio Ambiente – Rio de Janeiro (RJ), Brasil.

**Keywords:** Câncer ocupacional, Exposição ocupacional, Condições de trabalho, Fatores socioeconômicos, Saúde do trabalhador, Análise multivariada

## Abstract

**Objetivo:**

Explorar as relações conjuntas entre a mortalidade por câncer, fatores ocupacionais e características socioeconômicas entre servidores públicos brasileiros.

**Métodos:**

Trata-se de um estudo seccional com dados de 116 trabalhadores ativos da linha de base do Estudo Longitudinal de Saúde do Adulto (ELSA-Brasil) (2008–2010), que evoluíram a óbito por neoplasias malignas em um período de seguimento de 10 anos. Utilizou-se a análise de correspondência múltipla como forma de interpretar graficamente a associação entre ocupação, estresse no trabalho, jornada de trabalho, regime de trabalho e fatores socioeconômicos com a mortalidade por câncer.

**Resultados:**

A associação entre categorias de variáveis formou quatro grupos e possibilitou identificar dois grandes perfis distintos de trabalhadores. O primeiro foi caracterizado pelo sexo feminino, idade entre 50 e 72 anos, jornada de trabalho até 40 horas semanais, sem exposição ao trabalho noturno, regime de trabalho diarista, baixo desgaste no trabalho, nível educacional superior ou pós-graduação, trabalho ativo, ocupações não carcinogênicas e óbito por câncer não relacionado ao trabalho. O segundo perfil foi caracterizado pelo sexo masculino, nível educacional fundamental e médio, idade entre 35 e 49 anos, trabalho passivo, alto desgaste no trabalho, regime de trabalho plantonista, exposição ao trabalho noturno, ocupações carcinogênicas e óbito por câncer relacionado ao trabalho.

**Conclusão:**

O óbito por câncer relacionado ao trabalho foi associado a piores condições socioeconômicas e circunstâncias ocupacionais desfavoráveis à saúde do trabalhador.

## INTRODUÇÃO

O câncer relacionado ao trabalho (CRT) é a segunda principal causa de morte entre trabalhadores mundialmente. Em 2019, 2,9 milhões de mortes relacionadas ao trabalho ocorreram, sendo 29% causadas por neoplasias malignas^1,2^. No Brasil, a taxa de mortalidade por CRT teve tendência de aumento em mais da metade das unidades da federação (1990–2019)^3^. No Estudo Longitudinal de Saúde do Adulto (ELSA-Brasil), um dos maiores estudos epidemiológicos da América Latina, o câncer emergiu como a principal causa de óbito entre os trabalhadores participantes, ao longo de 10 anos de observação (2008–2018)^4^. Apesar da natureza multifatorial do câncer e da ausência de compreensão de todos os fatores envolvidos, o trabalho tem se destacado como um elemento de relevante contribuição para esse cenário.

Nesse sentido, a literatura aponta para um conjunto de tipologias de câncer que possui relação já estabelecida com agentes químicos, físicos ou biológicos aos quais o trabalhador é exposto em seu ambiente de trabalho^5^. Estudo recente utilizou dados das monografias da Agência Internacional para Pesquisa sobre Câncer (IARC) e listou 47 agentes do grupo 1, dos quais se podem citar o amianto, a radiação solar e o benzeno, associados aos cânceres hematológicos, de pulmão, bexiga, fígado, estômago, entre outros^6^. Há, portanto, suficientes evidências de que a exposição ocupacional a agentes carcinogênicos predispõe os indivíduos a neoplasias malignas e mortes geradas pela doença^6,7^.

Em contrapartida, pouco se sabe sobre outras circunstâncias de exposição intrínsecas ao processo de trabalho que também podem contribuir para o adoecimento e morte por câncer. Pesquisas sugerem que o estresse no contexto ocupacional^8^, longas jornadas de trabalho^9^, trabalho em turnos e noturno^10^ podem representar risco aumentado para certos tipos de câncer, como pulmão^8^ e mama^9,10^, contribuindo para o óbito^11^, contudo a literatura ainda é incipiente sobre o assunto e por vezes controversa, sobretudo ao tratar da associação entre tais exposições e mortalidade por câncer.

Até o presente momento, não se tem conhecimento de estudos nacionais que se dediquem a explorar as relações conjuntas de fatores ocupacionais e mortalidade por câncer, no contexto brasileiro. Portanto, para além das exposições já reconhecidamente carcinogênicas, e buscando contribuir para a elucidação das relações entre fatores ocupacionais e câncer, o objetivo deste estudo foi explorar as relações conjuntas entre a mortalidade por câncer, fatores ocupacionais e características socioeconômicas entre servidores públicos brasileiros que foram a óbito em um período de 10 anos de seguimento na coorte de trabalhadores (ELSA-Brasil).

## MÉTODOS

### Delineamento do estudo

Trata-se de uma análise seccional com dados do ELSA-Brasil, que é uma coorte multicêntrica que investiga fatores determinantes de doenças crônicas não transmissíveis e suas incidências. A linha de base (2008–2009) incluiu 15.105 servidores públicos, com idades entre 35 e 74 anos, de seis instituições de ensino superior nas regiões Nordeste, Sul e Sudeste do Brasil. A metodologia do estudo envolve o acompanhamento dos desfechos incidentes por meio de visitas a cada quatro anos e entrevistas anuais de seguimento, que monitoram eventos por telefone. Quando um evento é identificado, inicia-se uma investigação, acessando prontuários médicos para informações complementares. Dados sobre óbitos são coletados no setor de recursos humanos das instituições e no Sistema de Informações sobre Mortalidade. Os aspectos metodológicos do ELSA-Brasil estão detalhados em estudos anteriores^12,13,14^. Este estudo utilizou informações da linha de base da coorte (2008–2010) e dos óbitos que ocorreram até 31 de dezembro de 2018.

### Participantes

Foram incluídos os trabalhadores ativos da linha de base (2008–2010) do ELSA-Brasil que faleceram por câncer até 2018. Participantes aposentados foram excluídos por falta de informações sobre o ambiente psicossocial do trabalho, assim como aqueles com dados faltantes em outras variáveis.

### Variáveis

As variáveis utilizadas foram coletadas por meio de questionários aplicados nas entrevistas do ELSA-Brasil e categorizadas conforme descrito a seguir:

- Óbitos por CRT: os óbitos por câncer na coorte foram classificados como relacionados ao trabalho ou não relacionados ao trabalho, com base no grau de carcinogenicidade dos agentes ocupacionais definidos pela IARC^5^. Para a classificação como óbito por CRT, o tipo de câncer registrado deveria estar associado a pelo menos dois agentes cancerígenos (grupo 1), com os objetivos de trazer maior precisão à classificação, tornando a população de estudo mais heterogênea, e identificar diferentes grupos na análise de correspondência múltipla. Tipologias que não atenderam a esse critério foram classificadas como óbito por câncer não relacionados ao trabalho (Quadro 1);
- Ocupação: no ELSA-Brasil, as ocupações dos participantes foram definidas conforme a Classificação Brasileira de Ocupações (CBO), que possui quatro níveis hierárquicos: grandes grupos (GG), subgrupos principais (SGP), subgrupos (SG) e grupos de base (GB), sendo este último o mais específico e analisado neste estudo. O nível GB inclui título, descrição da ocupação, atividades principais e condições de trabalho, como exposições a agentes químicos, físicos e biológicos^15^. Com base na descrição das atividades e condições de trabalho extraídas da CBO, as ocupações foram avaliadas quanto à possível exposição a agentes carcinogênicos e categorizadas em dois grupos: carcinogênicas (exposição a agentes carcinogênicos do grupo 1 ou 2A da IARC)^5^ e não carcinogênicas (sem evidências de exposição a tais agentes em seus ambientes de trabalho) (Quadro 2);
- Regime de trabalho: foi categorizado em diarista, abrangendo trabalhadores que exercem suas atividades laborais de segunda a sexta-feira, em horários fixos, em período exclusivamente diurno e que durante toda a sua vida laboral nunca trabalharam por plantão; e plantonista, abrangendo trabalhadores que seguem escalas de trabalho que incluem fins de semana, feriados e horários tanto diurnos quanto noturnos, nos dias atuais ou anteriormente;
- Jornada de trabalho: subdivida em até 40 horas semanais e acima de 40 horas semanais;
- Exposição ao trabalho noturno: definida por meio de duas categorias distintas, trabalho noturno (sim), referindo-se aos indivíduos que realizam ou já realizaram em algum momento de sua vida profissional atividades laborais no período da noite (entre 22 e 5 h), conforme determinado pela legislação brasileira^16^; e trabalho noturno (não), englobando aqueles que nunca tiveram a experiência de trabalhar durante a noite;
- Estresse no trabalho: foi mensurado por meio da escala sueca Job Demand-Control-Support Model^17^, validada para uso no Brasil^18^, aplicada por pesquisadores treinados. A escala considera duas dimensões do ambiente psicossocial do trabalho: demanda psicológica, determinada por itens relacionados à carga de trabalho, ao ritmo das atividades e à dificuldade de realizá-las; e controle do trabalho, determinado por itens relativos ao uso de habilidades intelectuais e o grau de autonomia para tomada de decisões. Os escores obtidos, com variação de 5 a 20 pontos para cada dimensão, foram dicotomizados com base em suas medianas (15 pontos para demandas psicológicas e 17 para controle no trabalho). Isso culminou nos quatro quadrantes propostos por Karasek^19^: baixo desgaste no trabalho (baixa demanda/alto controle); trabalho ativo (alta demanda/alto controle); trabalho passivo (baixa demanda/baixo controle); e alto desgaste no trabalho (alta demanda/baixo controle);
- Idade: categorizada em dois grupos, 35–49 e 50–72 anos, considerando o aumento da mortalidade por câncer dos 50 anos em diante^20^;
- Sexo: categorizado em masculino e feminino;
- Escolaridade: classificada em três categorias: até ensino fundamental completo; ensino médio completo e ensino superior ou mais.

**Quadro 1:** Classificação das causas de morte por câncer na população de Estudo Longitudinal de Saúde do Adulto (ELSA-Brasil).

Tipologia de óbitos por câncer (ELSA-Brasil)	Agentes previstos no grupo 1 da Agência Internacional de Pesquisa em Câncer (IARC) que podem estar presentes no ambiente de trabalho	Classificação dos óbitos
**Pulmão**	Processo de Acheson; produção de alumínio; arsênico; asbestos; berílio; biclorometil éter, clorometil-metil-éter; cádmio; cromo (VI); gaseificação de carvão; piche de alcatrão de carvão; produção de coque; escape de motor a <italic>diesel</italic>; mineração subterrânea de hematita; fundição de ferro e aço; quimioterápicos combinados, incluindo agentes alquilantescompostos de níquel; poluição ao ar livre; exposição ocupacional como pintor; plutônio; radônio-222 e seus produtos de decomposição; indústria de fabricação de borracha; poeira de sílica, sílica cristalina na forma de quartzo ou cristobolita; fuligem; tabagismo passivo (tabaco); fumos de soldagem; radiação X e gama	O:CRT
**Mama**	radiação X gama	O:CNRT
**Cólon e reto**	radiação X gama	O:CNRT
**Fígado**	Aflatoxinas; vírus da hepatite B e C (infecção crônica); plutônio; tório-232; cloreto de vinila	O:CRT
**Estômago**	Radiação X e gama; indústria de fabricação de borracha	O:CRT
**Bexiga**	Produção de alumínio; 4-aminobifenila; arsênico; auramina; benzidina; ciclofosfamida; bombeiro; produção magenta; 2-naftilamina; pintor; fabricação de borracha; orto-toluidina; radiação ionizante	O:CRT
**Leucemia**	Benzeno; formaldeído; quimioterapias combinadas; fósforo-32; tório-232; radiação X e Gama; 1,3-butadieno indústria de fabricação de borracha	O:CRT
**Rim**	Tricloroetileno; radiação X gama	O:CRT
**Cérebro e sistema nervoso central**	radiação X gama	O:CNRT
**Colo uterino e ovário**	Asbestos; infecção [vírus da imunodeficiência adquirida (HIV) tipo 1]; papilomavírus humano tipos 6, 18, 31, 33, 35, 39, 45, 51, 52, 56, 58 e 59	O:CRT
**Mieloma**	1,3-Butadieno; pentaclorofenol	O:CRT
**Linfoma**	Vírus Epstein-Barr; HIV tipo 1 e hepatite C; azatioprina; ciclosporina; lindano; pentaclorofenol	O:CRT
**Aerodigestivo alto próstata/pâncreas outros cânceres**	-	O:CNRT

O:CRT: óbito por câncer relacionado ao trabalho, com base nas tipologias de câncer associadas a pelo menos dois agentes listados no Grupo 1 da IARC; O:CNRT: óbito por câncer não relacionado ao trabalho.

Fonte: Adaptado de IARC^5^.

**Quadro 2:** Classificação da variável ocupação na população de Estudo Longitudinal de Saúde do Adulto (ELSA-Brasil)*.

Ocupação	n	Descrição das atividades de trabalho segundo a Classificação Brasileira de Ocupações	Agente ou circunstâncias de exposição pertinentes ao exercício da ocupação	Classificação
Administradores	1	Planejam, organizam, controlam e assessoram as organizações	-	OC:NC
Contadores e afins	2	Gerenciam custos; administram o departamento pessoal	-	OC:NC
Escriturários em geral	13	Realizam serviços de apoio nas áreas de recursos humanos, administração e finanças	-	OC:NC
Farmacêuticos	1	Trabalham no desenvolvimento, produção, dispensação, controle, armazenamento, distribuição e transporte de medicamentos, imunobiológicos, domissanitários	Ciclofosfamida (1); clorambucil (1); MOPP (quimioterápicos) (1); hidrazina (2A); trabalho noturno (2A)	OC:C
Operadores de telefonia	1	Operam equipamentos, atendem, transferem, cadastram e completam chamadas telefônicas	-	OC:NC
Professores do ensino superior	18	Ensinam, realizam pesquisas científicas, orientam alunos	-	OC:NC
Profissionais da informação	6	Disponibilizam informação, gerenciam unidades como bibliotecas, centros de documentação	-	OC:NC
Profissionais de administração econômico-financeira	1	Preparam e consolidam informações gerenciais e econômico-financeiras	-	OC:NC
Profissionais de estatística	1	Desenham amostras; analisam e processam dados; constroem instrumentos de coleta de dados; criam banco de dados	-	OC:NC
Recepcionistas	5	Recepcionam e prestam serviços de apoio	-	OC:NC
Supervisores administrativos	24	Supervisionam rotinas administrativas, chefiando escriturários, auxiliares administrativos, secretários de expediente, operadores de máquina de escritório	-	OC:NC
Supervisores das artes gráficas	1	Administram o processo de produção gráfica com respeito a custos, viabilidade de execução, fluxo de tarefas	-	OC:NC
Trabalhadores nos serviços de coleta de resíduos, de limpeza e conservação de áreas públicas	34	Coletam resíduos sólidos de serviços de saúde, varrem, acondicionando o lixo, conservam as áreas públicas lavando-as e pintando. Atividades realizadas em recintos fechados ou a céu aberto. Trabalho em turno e noturno, exposição a ruído e poluição de veículos	Amianto (1); poeiras de sílica cristalina (1); radiação solar (1); benzeno (1); fumos de *diesel* (2A);	OC:C
Técnicos em enfermagem e auxiliares de enfermagem	2	Atividades técnicas de enfermagem em hospitais e clínicas. Administram medicamentos, atuam em instrumentação cirúrgica, podem ser expostos à contaminação biológica, material tóxico e à radiação. Trabalham sob pressão, elevando a situação de estresse	MOPP (quimioterápicos) (1); vírus (hepatites B e C; Epstein-Barr; imunodeficiência humana tipo 1; linfotrópico humano de células T tipo I (1); óxido de etileno (1); radiação ionizante (1);Trabalho noturno (2A);	OC:C
Técnicos em biblioteconomia	1	Trabalham em bibliotecas, escolas, institutos de pesquisa e estatística	-	OC:NC
Técnicos em controle ambiental, utilidades e tratamento de efluentes	1	Preservam a qualidade ambiental. Trabalham em laboratórios, em atividades de campo, e ambientes fechados, a céu aberto. Horários diurnos e noturnos. Expostos a ruídos, material tóxico, radiação.	Benzeno (1); cádmio (1); radiação solar (1); trabalho noturno; chumbo (2A)	OC:C
Técnicos em equipamentos médicos e odontológicos	3	Confeccionam e reparam próteses dentárias humanas, animais e artísticas. Podem estar sujeitos a exposições de material tóxico, radiação, pressão de trabalho que podem levar ao estresse	Radiação ionizante (1); níquel e seus compostos (1)	OC:C
Técnicos químicos	1	Operam processos químicos e operações unitárias de laboratório e de produção. Podem estar sujeitos a ruídos, poeira, gases, vapores e material tóxico	Cloreto de vinila (1); cádmio e seus compostos (1); benzeno (1); ácido sulfúrico (1); tricloroetileno (1)	OC:C

*Classificação da variável ocupação de trabalhadores ativos, na população de estudo ELSA-Brasil, segundo exposições ocupacionais, a pelo menos um agente químico, físico ou biológico, ou circunstância de exposição com potencial de causar câncer (grupo 1 e 2A da Agência Internacional de Pesquisa em Câncer), pertinentes ao exercício da ocupação; OC:C: ocupação carcinogênica; OC:NC: ocupação não carcinogênica. Descrição das atividades adaptado de CBO^15^.

### Análise estatística

Utilizamos a análise de correspondência múltipla (ACM) para explorar as relações conjuntas entre a mortalidade por câncer e fatores ocupacionais, incluindo as características socioeconômicas da população de estudo. As variáveis foram descritas por meio de frequências absolutas e relativas. A ACM possibilitou a representação gráfica do perfil de comportamento do grupo de variáveis qualitativas analisadas.

A análise de correspondência é uma técnica não inferencial aplicada a dados categóricos destinada a compor um padrão de associação entre as variáveis do estudo, por meio da medida de distância χ^2^ entre as categorias que compõem o conjunto de variáveis. Cada categoria possuiu uma posição espacial no gráfico da análise de correspondência, também denominado de mapa perceptual, e a proximidade desses pontos indica o grau de similaridade entre eles. Isso significa que categorias que estão próximas no espaço têm padrões semelhantes de associação entre si. Por sua vez, categorias que estão distantes no mapa têm padrões de associação diferentes^21^.

Essa técnica estatística apresenta uma medida de inércia, que indica a porcentagem de variabilidade explicada por dimensão. O número de dimensões selecionadas neste estudo se baseou no declínio das inércias ajustadas, ou seja, nos autovalores. O eixo x do gráfico de dispersão representa a variabilidade dos dados explicada pela primeira dimensão, enquanto o eixo y, a variabilidade dos dados explicada pela segunda dimensão. Os pontos representam cada uma das categorias de variáveis, enquanto as linhas delimitam os grupos associados a essas categorias^21^.

As análises foram realizadas no software R^16^, versão 4.3.2, biblioteca “ca”.

### Questões éticas

O ELSA-Brasil foi aprovado pelos Comitês de Ética em Pesquisa (CEP) de cada instituição envolvida e pelo Conselho Nacional de Ética em Pesquisa em 2006. O presente estudo foi aprovado pelo CEP da Escola Nacional de Saúde Pública (número de aprovação: 66407322.3.0000.5240).

## RESULTADOS

Dos 15.105 participantes do ELSA-Brasil, 12.096 eram trabalhadores ativos e 3.009 aposentados. Entre os ativos, 118 foram a óbito por câncer durante o período de seguimento. Entre os participantes elegíveis, dois foram excluídos por ausência de informação sobre a variável ocupação. Dessa forma, o tamanho da população de estudo foi 116.

Dos 116 óbitos, 46,6% ocorreram entre mulheres (n=54) e 53,4% entre homens (n=62). A idade variou de 35 a 72 anos (média=53,8; desvio padrão=7,8; mediana=54), 39,5% dos participantes têm nível superior ou mais (n=46), e 60,3% dos óbitos foram por câncer não relacionado ao trabalho (n=70) (Tabela 1).

**Tabela 1:** Distribuição das características socioeconômicas e ocupacionais segundo mortalidade por câncer relacionadas ou não ao trabalho, na população de Estudo Longitudinal de Saúde do Adulto (ELSA-Brasil), 2008–2010, 2018.

Variáveis e categorias	Total de óbitos por câncer (116)			
	Óbito por câncer relacionado ao trabalho n=46 (39,7%)		Óbito por câncer não relacionado ao trabalho n=70 (60,3%)	
	n	%	n	%
**Sexo**				
Masculino	28	45,2	34	54,8
Feminino	18	33,3	36	66,7
**Faixa etária (anos)**				
35–49	19	48,8	20	51,2
50–72	27	35,0	50	65,0
**Escolaridade**				
Fundamental ou menos	15	48,3	16	51,7
Médio	16	41,0	23	59,0
Superior ou mais	15	32,6	31	47,4
**Ocupação**				
Carcinogênica	24	54,6	20	45,4
Não carcinogênica	22	30,6	50	69,4
**Regime de trabalho**				
Diarista	25	39,0	39	61,0
Plantonista	21	40,3	31	59,7
**Jornada de trabalho (horas semanais)**				
Até 40	31	35,2	57	64,8
Acima de 40	15	53,6	13	46,4
**Trabalho noturno**				
Sim	19	43,2	25	56,8
Não	27	37,5	45	62,5
**Estresse no trabalho**				
Alto desgaste	12	42,9	16	57,1
Baixo desgaste	8	40,0	12	60,0
Trabalho ativo	6	26,0	17	74,0
Trabalho passivo	20	44,4	25	55,6

Fonte: (ELSA-Brasil). Linha de base (2008–2010) e base de adjudicação de óbitos (2018).

Na ACM, as duas primeiras dimensões concentraram a maior proporção da variabilidade explicada (68,4%). No mapa perceptual bidimensional é possível visualizar dois grandes perfis distintos e quatro grupos subscritos em quadrantes (Figura 1).

**Figura 1:** Mapa perceptual (*plot* bidimensional) da análise de correspondência múltipla entre participantes do Estudo Longitudinal de Saúde do Adulto (ELSA-Brasil) que foram a óbito por câncer 2008–2010, 2018.

OC:C: ocupação carcinogênica; OC:NC: ocupação não carcinogênica; TN:E: exposição ao trabalho noturno; TN:NE: não exposição ao trabalho noturno; ET:ADT: alto desgaste no trabalho; ET:BDT: baixo desgaste no trabalho; ET:TP: trabalho passivo; ET:TA: trabalho ativo; TU:D: turno diarista; TU:P: turno plantonista; JT:≤40H: jornada de trabalho até 40 horas semanais; JT:>40H: jornada de trabalho acima de 40 horas semanais; SEX:M: sexo masculino; SEX:F: sexo feminino; E:S: ensino superior ou mais; E:M: ensino médio completo; E:F: ensino fundamental ou menos; I:35-49: idade entre 35 e 49 anos; I:50-72: idade entre 50 e 72 anos; O:CRT: óbito por câncer relacionado ao trabalho; O:CNRT: óbito por câncer não relacionado ao trabalho.

O perfil localizado à esquerda do gráfico reúne características socioeconômicas e ocupacionais, como alto grau de escolaridade, idade mais avançada, sexo feminino, não exposição ao trabalho noturno, regime de trabalho diarista, jornada de trabalho limitada a 40 horas semanais, baixo desgaste no trabalho, ocupações não carcinogênicas, trabalho ativo e óbito por câncer não relacionado ao trabalho.

Já o perfil localizado à direita do gráfico apresenta características expressamente opostas: baixo grau de escolaridade, idade mais jovem, sexo masculino, exposição ao trabalho noturno, regime de plantão, jornada de trabalho que excede 40 horas semanais, alto desgaste no trabalho, ocupação carcinogênica, trabalho passivo e óbito por CRT.

A respeito dos grupos de variáveis com similaridades, observados em quadrantes no gráfico, pode-se dizer: o primeiro grupo formado (quadrante superior esquerdo) é caracterizado por idade entre 50 e 72 anos, jornada de trabalho até 40 horas semanais, não exposição ao trabalho noturno e regime de trabalho diarista.

O segundo grupo (quadrante inferior esquerdo) é caracterizado pelo sexo feminino, nível educacional superior ou pós-graduação, trabalho ativo, ocupações classificadas como não carcinogênicas, baixo desgaste no trabalho, e óbito por câncer não relacionado ao trabalho.

Já o terceiro grupo (quadrante superior direito) foi formado pelo sexo masculino, ensino fundamental, trabalho passivo, ocupações carcinogênicas e óbito por CRT.

No último grupo (quadrante inferior direito) podemos observar idade entre 35 e 49 anos, ensino médio, alto desgaste no trabalho, atividade laboral em regime de plantão e exposição ao trabalho noturno.

## DISCUSSÃO

Neste estudo, por meio da ACM, foram analisadas as relações entre fatores ocupacionais e socioeconômicos e câncer. As piores condições de trabalho (trabalho passivo e ocupações carcinogênicas), baixo nível educacional (ensino fundamental) e sexo masculino foram associados ao óbito por CRT. Em contrapartida, as melhores condições de trabalho, altos níveis de escolaridade e sexo feminino foram associados com o óbito por tipologias de câncer não relacionadas ao trabalho.

Alguns autores relataram a predominância de homens com ensino fundamental ou médio entre os casos de CRT^22,23^. Nossos resultados mostraram diferenças entre os sexos, sugerindo que a maior prevalência de homens em atividades laborais de maior risco de exposição a carcinógenos contribui para o perfil de óbitos por CRT. No estudo de Nogueira et al.^24^, com dados da Pesquisa Nacional de Saúde (2019), 49% dos homens estavam expostos a pelo menos um agente cancerígeno do grupo 1 e 31,9% ao grupo 2 da IARC, enquanto entre as mulheres esses percentuais foram 16,9 e 16,5%, respectivamente. Segundo Silva et al.^25^, os homens, em comparação com as mulheres, procuram menos os serviços de saúde. Por conseguinte, o diagnóstico e tratamento de doenças como o câncer ocorrem tardiamente, piorando o prognóstico.

No aspecto educacional, embora o ELSA-Brasil seja constituído de servidores públicos federais, 70% dos integrantes da coorte na linha de base tinham até o ensino médio. Destes, 50% desempenhavam funções de apoio, exigindo apenas ensino fundamental incompleto e 50% funções de nível médio^13^. De acordo com Silva et al.^26^, profissionais menos qualificados enfrentam maiores riscos de exposição a substâncias como benzeno, amianto, metais pesados e outros agentes cancerígenos. Tais condições são verificadas em maior intensidade em países em desenvolvimento^27^, como o Brasil, e puderam ser observadas em nosso estudo, ao analisarmos as condições gerais de exercício e exposições pertinentes a ocupações presentes na coorte (Quadro 2).

Nesse sentido, destacamos as ocupações relacionadas aos serviços de coleta de resíduos, limpeza e conservação de áreas públicas exercidas por trabalhadores ativos participantes do ELSA-Brasil. Por causa das atividades de trabalho, esses profissionais podem estar expostos a radiação solar, poeiras minerais, como sílica e amianto, e substâncias químicas, como o benzeno (detalhamento disponível em Material Suplementar).

Corroborando com nossos achados, recente estudo sobre prevalência de carcinógenos ocupacionais na população brasileira, com dados da Pesquisa Nacional de Saúde, revelou que 64,4% dos coletores de lixo e trabalhadores similares do sexo masculino estão expostos a pelo menos um carcinógeno do grupo 1, e 29%, a pelo menos um carcinógeno do grupo 2, com destaque para as seguintes exposições: radiação solar (58,2%), poeiras minerais (14,8%) e agentes químicos (16%)^24^. Nossos achados convergem com essa pesquisa e com a de Bovio et al.^28^; ambas reforçam a estabelecida associação entre ocupação e exposição a agentes cancerígenos, propiciando excesso de morte por câncer entre trabalhadores.

A análise conjunta das variáveis estudadas revelou não apenas a influência da exposição a agentes cancerígenos, mas também de fatores socioeconômicos e do ambiente psicossocial no trabalho sobre as mortes por CRT, aspecto ainda pouco explorado no Brasil. Pesquisadores discutem o papel do estresse psicossocial sobre efeitos adversos à saúde, ocorrência de doenças e morte^29,30^, entretanto sua participação no desenvolvimento e prognóstico de tumores malignos, sobretudo no contexto do trabalho, ainda é controversa.

Nesse sentido, alguns estudos internacionais apontam para associação positiva entre o estresse psicossocial no trabalho e o desenvolvimento de tumores malignos^31^ e metástase^32^, enquanto outros não identificam essa associação^33,34,35^.

Apesar de não haver consenso entre os estudos atuais, nossa interpretação baseia-se na plausibilidade biológica de que o estresse psicossocial no trabalho favorece a ativação do câncer primário e a progressão acelerada do tumor. Esse processo envolve a ativação do eixo hipotálamo-hipófise-adrenal e do sistema nervoso, liberando hormônios como cortisol e norepinefrina, que alteram o microambiente tumoral, promovem inflamação, aumentam a expressão de oncogenes e suprimem a resposta imunológica^36^. Dessa forma, é possível supor que o estímulo persistente de estressores psicossociais, como baixo poder de decisão e baixa demanda, pertinentes ao trabalho passivo, representam uma condição de estresse crônico relacionado ao trabalho. Apesar de esses estressores não serem capazes de, isoladamente, causar o câncer, associados a outros fatores ocupacionais aqui mencionados, podem contribuir para o óbito por CRT. Cabe esclarecer que o trabalho passivo é caracterizado pela baixa demanda e falta de controle das tarefas, limitando habilidades intelectuais, gerando desânimo, frustração e desinteresse pelo trabalho.

Outra dimensão do estresse em âmbito ocupacional aqui investigado foi o alto desgaste, representado por altas demandas psicológicas e baixo controle para tomada de decisão no trabalho^18^. Em um estudo de coorte sueco, o baixo controle e as elevadas exigências laborais foram levemente associados ao aumento do risco de câncer de mama entre mulheres^37^. Nossos resultados apontaram que o alto desgaste no trabalho se associou a outras categorias de variáveis ocupacionais: regime de trabalho plantonista e exposição ao trabalho noturno. Tais fatores ocupacionais, *a priori* não classificados no grupo 1 como reconhecidamente cancerígenos para humanos, de acordo com a literatura científica atual, podem contribuir para uma piora no estado de saúde^38,39^, por alterar os hábitos de vida saudáveis^40^, afetar o sistema imunológico^41^, desregular o ritmo circadiano^42^, propiciando um ambiente fisiológico favorável para a iniciação de células cancerígenas, assim como o desenvolvimento de outros agravos à saúde, de ordens física e mental^43^.

A hipótese do presente estudo foi que as variáveis ocupacionais alto desgaste, exposição ao trabalho noturno e regime de trabalho plantonista, por terem padrão de similaridade, podem contribuir para a construção de um perfil de pessoas com maior propensão ao adoecimento por câncer. Destacamos que a não associação das variáveis ocupacionais mencionadas com o óbito por CRT em nossa investigação possivelmente se deva ao fato de as tipologias de câncer associadas a essas exposições (mama, próstata e cólon-reto) não terem sido classificadas neste estudo como óbitos por CRT, conforme critérios estabelecidos para a classificação dessa variável (mais detalhes, consultar Material Suplementar).

Um aspecto curioso em nosso estudo se refere à carga horária de trabalho. A categoria jornada de trabalho acima de 40 horas semanais se associou a sexo feminino e trabalho ativo (com altas demandas e alto poder de decisão); ocupações não carcinogênicas; óbito por câncer não relacionado ao trabalho; e alta escolaridade, contrapondo nossas hipóteses iniciais de que longas jornadas de trabalho estivessem associadas a piores condições laborais, propensão ao desenvolvimento e morte por câncer.

Embora não seja possível descartar por completo essa possibilidade, atualmente, poucos estudos têm relatado associações entre longas jornadas de trabalho e câncer. O que se tem observado na literatura é a associação dessa variável com outros desfechos negativos em saúde, como doenças cardíacas e obesidade^44,45^, mas não há achados conclusivos sobre a incidência ou mortalidade por neoplasias malignas^9,46^. Diante disso, sugerimos que a ausência de associação entre jornadas de trabalho superiores a 40 horas semanais e óbitos por CRT em nosso estudo possa em parte refletir esses achados verificados na literatura.

Esse é o primeiro estudo a avaliar dados de mortalidade por câncer em trabalhadores da coorte ELSA-Brasil. Entre as vantagens do método utilizado, podemos mencionar a possibilidade de uma análise exploratória robusta, com abordagem flexível e visualmente intuitiva. Ela nos permitiu descrever de forma clara o perfil dos trabalhadores participantes do estudo e as complexas relações existentes entre fatores ocupacionais e socioeconômicos e a mortalidade por câncer, superando as restrições de métodos estatísticos tradicionais com rigorosos pressupostos de normalidade dos dados e linearidade das relações.

Em contrapartida, reconhecemos que este estudo tem limitações, pois se trata de uma investigação com a utilização de ferramentas não inferenciais e uma amostra relativamente pequena, pois a intenção era explorar apenas os dados dos participantes ativos que foram a óbito por câncer. Além disso, o ELSA-Brasil não é um estudo específico para câncer, e a classificação de óbitos por CRT apresenta desafios. Apesar disso, adotamos um critério de maior especificidade para essa classificação, no intuito de torná-la mais precisa. Ainda em relação ao óbito, por ser um evento complexo, não se pode garantir que sua causa seja estritamente relacionada ao câncer primário. Ressalta-se que o desenho de estudo não permite a avaliação de risco.

Nossos resultados revelaram que exposições como trabalho passivo, ocupações carcinogênicas e baixa escolaridade, em conjunto, se associam à mortalidade por CRT, o que pode significar que piores condições socioeconômicas, ocupações com maior possibilidade de exposição a agentes carcinogênicos no ambiente de trabalho e circunstâncias ocupacionais desfavoráveis à saúde apresentam padrão de associação semelhante, formando um perfil característico de pior prognóstico da doença. Diante disso, fazem-se necessárias a implementação de políticas e ações de saúde do trabalhador capazes de contribuir para a melhoria das condições laborais, com foco na qualificação e valorização do profissional, e a adoção de estratégias de redução efetiva dos riscos de exposição a fatores ocupacionais mencionados, a fim de promover um ambiente de trabalho mais saudável e seguro, prevenindo o adoecimento e morte por CRT.

## Data Availability

O estudo é resultado da tese de doutorado do programa de pós-graduação da Escola Nacional de Saúde Pública, subárea de Epidemiologia em Saúde Pública, aprovada pelo Comitê de Ética em Pesquisa, sob o número: 66407322.3.0000.5240, realizado com financiamento próprio. O ELSA-Brasil foi financiado pelo Ministério da Saúde (Departamento de Ciência e Tecnologia) e pelo Ministério de Ciência, Tecnologia e Inovação (Financiadora de Estudos e Projetos e Conselho Nacional de Desenvolvimento Científico e Tecnológico, números de bolsas: 01 06 0010.00 RS, 01 06 0212.00 BA, 01 06 0300.00 ES, 01 06 0278.00 MG, 01 06 0115.00 SP e 01 06 0071.00 RJ).
