# Supplementary material for: ERRATUM
Source: Rev Bras Epidemiol. 2025 Jun 27;28:e20250022erratum. doi: 10.1590/1980-549720250022erratum (PMC12204234; doi:10.1590/1980-549720250022erratum)
Supplement: Translation

# Translation: ERRATA

**DOI:** 10.1590/1980-549720250022.2erratum

No artigo "*Fatores ocupacionais e socioeconômicos e mortalidade por câncer em participantes do Estudo Longitudinal de Saúde do Adulto (ELSA-Brasil): uma análise de correspondência múltipla*", DOI: https://doi.org/10.1590/1980-549720250022.2, publicado no periódico Rev Bras Epidemiol 2025; 28: e250022.

## Título:

**Onde se lia:**

Occupational, socioeconomic factors and cancer mortality in participants of the Longitudinal Study of Adult Health (ELSA-Brazil): a multiple correspondence analysis

**Leia-se:**

Occupational, socioeconomic factors and cancer mortality in participants of the Longitudinal Study of Adult Health (ELSA-Brasil): a multiple correspondence analysis

## Abstract:

**Onde se lia:**

This is a cross-sectional study with data from 116 active workers at the baseline of the Longitudinal Study of Adult Health (ELSA-Brazil) (2008–2010), who died of malignant neoplasms over a 10-year follow-up period.

**Leia-se:**

This is a cross-sectional study with data from 116 active workers at the baseline of the Longitudinal Study of Adult Health (ELSA-Brasil) (2008–2010), who died of malignant neoplasms over a 10-year follow-up period.
